# Rapid and user-friendly open-source CRISPR/Cas9 system for single- or multi-site editing of tomato genome

**DOI:** 10.1038/s41438-018-0082-6

**Published:** 2019-01-01

**Authors:** Nan Hu, Zhiqiang Xian, Ning Li, Yudong Liu, Wei Huang, Fang Yan, Deding Su, Jingxuan Chen, Zhengguo Li

**Affiliations:** School of Life Sciences, Chongqing University, Chongqing, 405200 People’s Republic of China

**Keywords:** Genetic engineering, Molecular engineering in plants

## Abstract

CRISPR/Cas9-induced genome editing is a powerful tool for studying gene function in a variety of organisms, including plants. Using multi-sgRNAs to target one or more genes is helpful to improve the efficacy of gene editing and facilitate multi-gene editing. Here, we describe a CRISPR/Cas9 system which can be conveniently developed as a CRISPR kit. SgRNA expression cassettes can be rapidly generated by one-step PCR using our CRISPR kit. In our kit, there are two binary vectors pHNCas9 and pHNCas9HT. The binary vector pHNCas9 was constructed to allow to assemble up to eight sgRNA expression cassettes by one-step Golden Gate cloning. Another binary vector pHNCas9HT can be used to generate a large number of single target constructs by directly transforming ligation reactions products into *A. tumefaciens* without several procedures, such as PCR and plasmid extraction. The two binary vectors are designed according to the principles of standard BioBrick assembly to be used as an open-source tool. For example, we used BioBrick as a visual T-DNA tag. We also developed a primer design aid to complement the system. With this primer design aid, researchers can rapidly obtain primers and GC content, and sgRNA sequence of target site. Our CRISPR/Cas9 system can perform single- and multi-site editing and multiple gene editing to produce various types of mutations in tomato. This rapid and user-friendly CRISPR/Cas9 system for tomato can be potentially used for mutagenesis of important crop species for genetic improvement and is suitable for research into the function of genes.

## Genetic engineering: Quick and easy CRISPR in tomato

Researchers in China have created a toolkit to easily mutate genes in tomato using the CRISPR gene modification technology. Zhengguo Li of Chongqing University developed the system in response to the lack of an open, standardized, reliable gene editing tool in tomato. The toolkit is designed according to an open-source, modular standard known as Biobrick. It consists of several molecular building blocks which can be used to target up to eight genes for editing or deletion. The kit also includes software to help researchers determine the DNA sequences necessary to copy genes of interest and prepare them for the CRISPR reactions. Li’s team tested the toolkit by using it to successfully simultaneously mutate multiple genes involved in tomato ripening. The new toolkit will enable researchers to rapidly and easily manipulate genes in this important crop.

## Introduction

Reverse genetics is a fundamental way of studying gene function in plants, where a gene of interest is knocked out or its expression is attenuated to deduce the normal function of the gene from the observed phenotypic effects^1^. Classical reverse genetic strategies to target genes of interest include antisense inhibition, virus-induced gene silencing, and post-transcriptional gene silencing (PTGS) based on double-stranded RNA, either driven by small interfering RNA or artificial micro RNAs^1–4^. A drawback of PTGS is its instability from tissue to tissue or generation to generation, as the small RNAs can also induce methylation of the DNA inserted in transcriptional units of the genome^5^. Naturally occurring or induced mutations or T-DNA insertions can be informative about gene function research in model species. For example, the vast pools of *Arabidopsis thaliana* T-DNA mutants have been generated and screened to identify the mutations in genes of interest when the gene sequences and T-DNA are known. However, this approach is not applicable in most plant species^6^. Map-based cloning can be complex and difficult when genetic mutations are induced by ethyl methanesulfonate or fast neutrons^7^. Several genome editing systems are currently being used to circumvent these problems in a variety of organisms, including non-model plants. Over the past decade, two protein-based DNA targeting systems, zinc-finger nucleases (ZFNs), and transcription activator-like effector nucleases (TALENs) have been utilized to perform site-directed changes in the genome. The RNA-guided genome editing system CRISPR/Cas9 system (type II clustered regularly interspaced short palindromic repeats/associated protein system) has recently revolutionized genetic research^8^. These bacterial nucleases can recognize a specific DNA sequence and cleave it at the target site to generate double-strand breaks (DSBs). Two primary repair mechanisms, namely, homologous-directed repair (HDR) and error-prone nonhomologous end-joining (NHEJ), are available repairing for DSBs^9,10^. When a DSB is repaired by the NHEJ pathway, various mistakes, such as small insertions and deletions, are often created in or near the target locus. Therefore, if NHEJ occurs in an open reading frame or regulatory motif, then the mutations may induce frameshifts or disrupt a regulatory motif. The HDR pathway faithfully inserts a donor DNA segment with a strongly homologous sequence in the targeted site and can be exploited to incorporate a mutation of interest.

The versatility and efficiency of genome editing of CRISPR/Cas9 technology^11,12^ render it suitable for crop breeding research^13–15^. The CRISPR/Cas9 system has been deployed in a variety of important plant species^6^ and is simpler, more efficient, and more flexible than ZFNs and TALENs^8^. The CRISPR/Cas9 system has three components: Cas9 protein and two short RNAs named CRISPR RNA (crRNA) and trans-activating CRISPR RNA (tracrRNA). CrRNA and tracrRNA have been shown to form an integrated RNA structure defined as guide RNA (gRNA), which contains 20 specific nucleotide sequences that bind to the target DNA^16,17^. The protospacer adjacent motif, NGG, is located at 3ʹ downstream of the target sequence and is necessary for the CRISPR/Cas9 system to recognize and cleave the target site to generate DSBs^18^.

In animals, single-guide (sg) RNAs and *Cas9* mRNA can be co-injected into a one-cell embryo^13,19^. However, it is difficult to conduct this method in plant duo to the surrounding cell wall. Instead, an Agrobacterium-mediated gene transformation method has been found to be suitable for delivering a T-DNA containing sgRNA–Cas9 sequences into plant cells^20^. One limitation of early CRISPR/Cas9 systems in plants is that they usually recognize only one or two target sites. However, it has been shown that targeting multiple sgRNAs to the same gene can considerably increase the editing efficiency^10,21^. Combining multiple sgRNAs can also extend the usefulness of Cas9 as a mutagen by increasing the efficiency of deleting large segments of the genome^22^. Occasionally locating genome editing at different sites simultaneously is also desirable. Several multi-site CRISPR/Cas9 systems have been developed to achieve this phenomenon in plants^6,21,23–27^, for example, using Golden Gate, Gibson Assembly, or compatible enzymes for assembly of multiplex gRNA cassettes. Although these methods have been demonstrated, a variety of shortcomings still exist in such methods. Some methods can only assemble a few sgRNA expression cassettes^23,25^, while others require several steps of subcloning^24,26,27^. The common shortcoming of these systems is the lack of standardization to support open-source adoption and development of tools.

Tomato is an important horticultural crop. A CRISPR/Cas9 system can be a direct and efficient tool to generate new varieties without introducing foreign genes into the plant genome. These varieties can be used as non-transgenic germplasm resources, which might be acceptable if they are considered as non-transgenic crops^28^. Herein we introduced an open-source, developable, and modular system for plant genome engineering to facilitate gene function research in tomato and potentially in other crops. With the application of standardized BioBrick technology, our system can be easily upgraded and adjusted to accommodate novel expression elements^29,30^. BioBrick technology is a synthetic biology technology based on the same-tail restriction enzyme. It allows BioBrick modules to be continuously assembled without damaging the BioBrick sites. A similar multipurpose open-source system was recently published by Cermak et al.^31^. They used Golden Gate cloning to assemble different functional modules for different applications. Differently from Cermak and colleagues, we used standardized BioBrick technology to achieve the similar purpose. We have developed a *Cas9* system containing two binary vectors pHNCas9 and pHNCas9HT. pHNCas9HT can be used for the construction of sgRNA expression cassettes without PCR and direct transformation of *Agrobacterium tumefaciens*. Multiple sgRNA expression cassettes can be assembled into the pHNCas9 in one pot by Golden Gate ligation. Golden Gate ligation uses the type II restriction endonucleases, such as *Esp3* I and *Bsa* I, to design and generate non-palindromic sticky ends of sequences, which can avoid self-ligation and incompatible end ligation. Thus, this method is suitable for linking multiple DNA fragments in a single reaction in a designed order. Furthermore, our system can be easily developed into a rapid and user-friendly open-source CRISPR/Cas9 kit for single- or multi-site editing.

We aimed to illustrate the potential use of a BioBrick by designing a visual T-DNA marker based on the overexpression of *AtMYB75/PAP1* (driven by 35S promoter)*. AtMYB75-*overexpressed lines exhibit purple stems, leaves, and flowers due to the abnormal accumulation of anthocyanins in these tissues^32^. Using the genome editing system containing a visual marker, transformants can be easily screened by the plant color, which can indicate whether they possess the T-DNA containing CRISPR/Cas9 and the visual BioBrick insert in T0 and offspring. According to Mendelian genetics, heterozygous transgenic lines of the offspring undergo genetic separation. Therefore, we can screen for non-transgenic mutants (no T-DNA insertion and *AtMYB75* expression element) based on whether the plants indicate a purple color. We developed a primer design aid that contains macros and Visual Basic function for our CRISPR/Cas9 system using Microsoft Excel to further facilitate the uptake and use of the tomato genome editing system. This gadget (Excel S1) can help researchers design primers for numerous gene-specific target site and provide sgRNA sequences containing target sites for secondary structure prediction of sgRNAs. We targeted single sites in *SlEIN2, SlERFE1*, and *SlARF2B* gene; three sites in *SlGRAS8* gene; and two sites in SlACS2 and SlACS4 genes in tomato to test the tomato genome editing ability of our system.

The members of the large *GRAS* transcription factor family are crucial for plant development and defense. However, information on this gene family is limited in tomato. As reported, the expression of *SlGRAS8* (Solyc02g085600.1) was significantly higher in immature green fruit than in other tomato tissues^33^, suggesting that the SlGRAS8 gene may play a role in fruit development. *SlEIN2* (Solyc09g007870.2)*, SlERFE1* (Solyc09g075420.2), and *SlARF2B* (Solyc12g042070.1) were reported to be closely related to the ethylene signaling pathway^34–36^. These genes participate in the regulation of fruit maturation. *SlACS2* (Solyc01g095080.2) and *SlACS4* (Solyc05g050010.2) played a key role at the ripening stage of tomato fruit^37,38^ and may be important target genes for tomato CRISPR breeding. Herein, we demonstrated the effectiveness of our CRISPR/Cas9 system by targeting *SlGRAS8*, *SlEIN2*, *SlERF.E1*, *SlARF2B*, *SlACS2*, and *SlACS4*.

## Materials and methods

### Vector construction

**Fig. 1:**
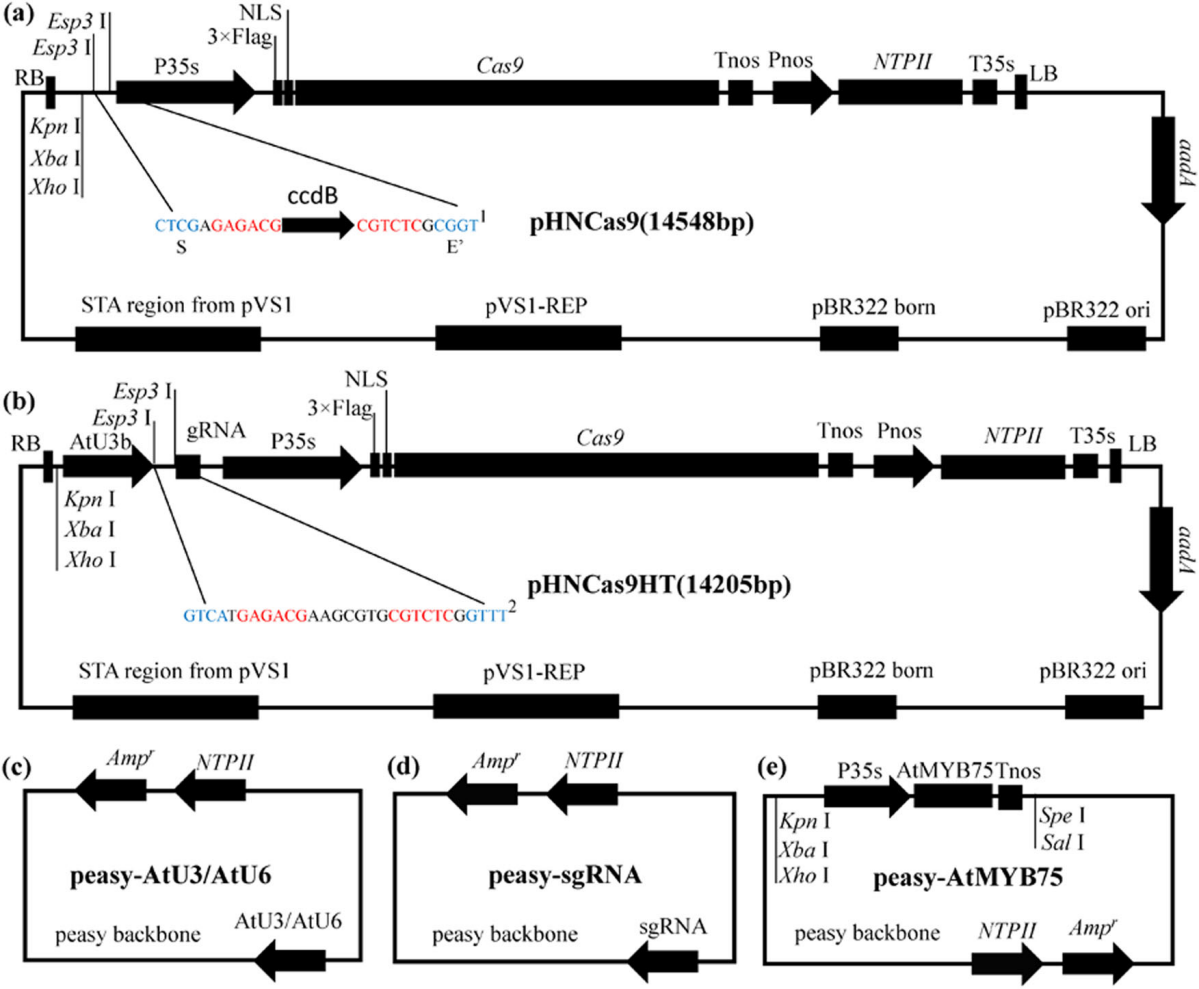
A CRISPR/Cas9 system for tomato plants. **a** Structure of the pHNCas9 binary vector is based on the pCAMBIA (Cambia, Canberra, Australia) backbone. NPT II encodes neomycin phosphotransferase II. NLS, nuclear localization sequence; ^1and 2^: *Esp3* I restriction sites for cloning are shown in red*, Esp3* I cutting sites (Golden Gate site) are shown in blue; S, Golden Gate Site S was used to link the TS′ site of the sgRNA expression cassettes to the pHNCas9 binary vector. E′: Golden Gate Site E′ was used to link the TE site of the sgRNA expression cassettes to the pHNCas9 binary vector. The ccdB: toxic ccdB gene (a negative selectable marker). **b** Structures of the pHNCas9HT binary vector based on the pHNCas9. pHNCas9HT already contains a sgRNA expression cassette driven by AtU3b. We designed two *Esp3* I restriction sites between AtU3d and sgRNA. **c** Overall structures of pEASY-AtU3b, pEASY-AtU3d, pEASY-AtU6-1, pEASY-AtU6-26, and pEASY-AtU6-29. **d** Structure of the pEASY-sgRNA vectors. **e** Structure of the pEASY-OE*AtMYB75* vectors

The pCAMBIA vectors are widely used for Agrobacterium-mediated transformation of a range of plant species^39,40^. In our system, the backbone of the binary vector was based on pCAMBIA (Cambia, Canberra, Australia) with a kanamycin-resistance gene driven by the NOS promoter. A *Cas9* gene^41^ (optimized in tobacco by Professor Qingyou Xia of Southwest University in Chongqing) was placed after 35S promoter. The sequence of *Cas9* is provided in Text S1. We designed and introduced BioBrick-compatible sites (*Kpn* I, *Xba* I, *Xho* I) and two Golden Gate-compatible *Esp3* I restriction sites between the 35S promoter and the right border T-DNA repeat for assembly of sgRNA expression cassette. The ccdB gene is located between two *Esp3* I sites. The binary vector was named pHNCas9 (Fig. 1a). In addition, another binary vector, pHNCas9HT (Fig. 1b), was developed based on pHNCas9. The difference between pHNCas9HT and pHNCas9 is that the former already contains a sgRNA expression cassette driven by AtU3b (Text S4). The two *Esp3* I sites are located between AtU3d and sgRNA sequences, and BioBrick site (*Kpn* I, *Xba* I, and *Xho* I) is in front of the AtU3b (Fig. 1b).

We constructed five different Pol III-dependent promoter module vectors named pEASY-AtUx to ligate multiple sgRNA expression cassettes into a single binary vector for multiplex genome editing, where x indicates the promoter used. The Pol III-dependent promoters were AtU3b, AtU3d, AtU6-1, AtU6-29, and AtU6-26, which have all been widely used in the dicotyledonous plant CRISPR system^42^ (Fig. 1c). A sgRNA module vector called pEASY-sgRNA was also created (Fig. 1d). The sequences of AtUx and sgRNA came from reports of Ma et al.^21^ and Junping Gao et al.^41^ and are provided in Text S2. Using these vectors, obtaining the AtU3/AtU6 promoter and sgRNA, which can then be used to amplify the sgRNA expression cassettes by one-step PCR, is simple. Using Golden Gate cloning strategy, multiple sgRNA expression cassettes can then be readily assembled^21,43^.

An *AtMYB75/PAP1* overexpression function module (OE*AtMYB75* BioBrick) was developed based on the standard assembly of BioBrick technology to enrich the functionality of this system. In this module, *AtMYB75*/*PAP1* was driven by 35S promoter, and an *Spe* I site originally existing in *AtMYB75*/*PAP1* was disrupted by PCR. BioBrick-compatible sites *Kpn* I, *Xba* I, and *Xho* I were in front of the 35S promoter and *Spe* I and *Sal* I were behind the NOS terminator. *Xba* I and *Spe* I are compatible restriction sites similar to *Xho* I and *Sal* I. This BioBrick was cloned into the pEASY-cloning vector (Transgene, China) called pEASY-OE*AtMYB75* (Fig. 1e). The sequence of the BioBrick is provided in Text S5.

### Make your own CRISPR kit

**Fig. 2:**
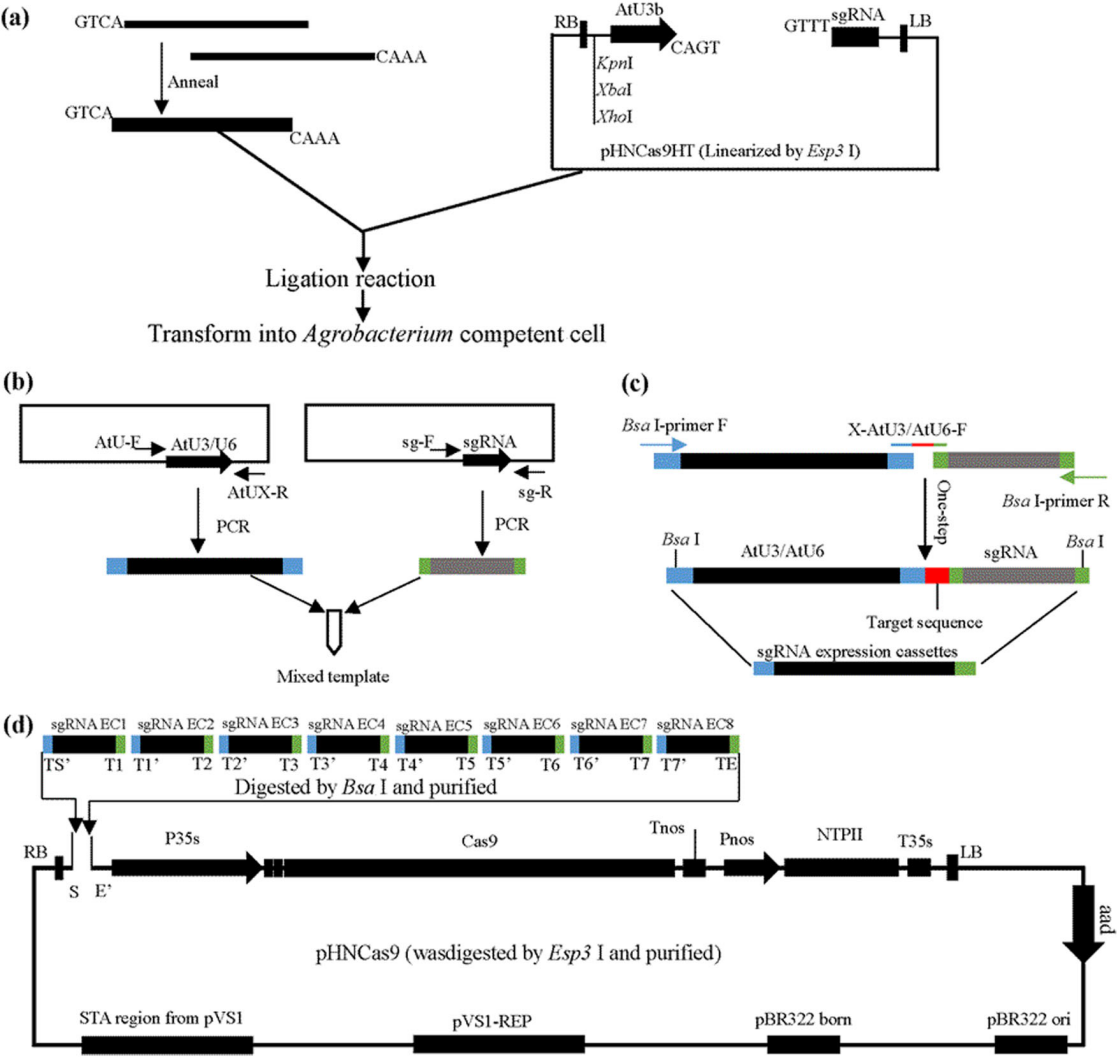
Overview of single target site or multiple sgRNA expression cassette assembly. **a** Cloning of single target site in the pHNCas9HT and transformation in *Agrobacterium* competent cells. pHNCas9HT were digested by *Esp3* I and purified. **b** Preparation of mixture of templates for AtU3b-sgRNA, AtU3d-sgRNA, AtU6-1-sgRNA, AtU6-26-sgRNA, and AtU6-29-sgRNA (all at 1 ng/µl). AtU-F is a universal primer for the five AtU promoters. AtUX designates specific primers for AtU3b-R, AtU3d-R, AtU6-1-R, AtU6-26-R, and AtU6-29-R, respectively. **c** The sgRNA expression cassettes with target site were generated by one-step PCR in one pot. **d** Assemble multiple sgRNA expression cassettes in pHNCas9. The pHNCas9 was digested by *Esp3* I and purified

First, AtU3b, AtU3d, AtU6-1, AtU6-29, and AtU6-26 were amplified in 50 µl with 0.2 µM each of AtU-F and the corresponding AtUx-R primer using TransStart FastPfu Fly DNA Polymerase (Transgene, China) for 50 cycles (Fig. 2b). sgRNA was amplified with sg-F and sg-R primers (Fig. 2b). AtU-F, AtUx-R, sg-F, and sg-R primer sequences are provided in Table S1. PCR products were purified using the gel Pure kit (Magen, China). The purified PCR products, 1 ng/µl of the promoter and 1 ng/µl of the sgRNA fragment, were mixed to form templates named AtU3b-sgRNA, AtU3d-sgRNA, AtU6-1-sgRNA, AtU6-29-sgRNA, and AtU6-26-sgRNA (Fig. 2b). The pHNCas9 plasmid (2 µg) was digested in 20 µl reaction with 1 µl of FastDigest *Esp3* I (Thermo, USA) and purified using a gel Pure kit (Magen, China). The linearized pHNCas9 was configured as a 25 ng/ul stock solution named Cas9VL. pHNCas9HT was fully digested by FastDigest *Esp3* I (Thermo, USA) and purified using a gel Pure kit (Magen, China). The linearized pHNCas9HT was configured as a 25 ng/ul stock solution named Cas9VLHT. *Bsa* I-site primers (Table S2) used for one-step PCR of sgRNA expression cassettes were also included. We recommend that *Esp3* I be used once because it is less stable. Therefore, a complete CRISPR kit includes AtUX-sgRNA mix template, Cas9VL, Cas9VLHT, and *Bsa* I-site primers (usage was provided in Table S3). The CRISPR kit is sufficient for several hundred times clone of sgRNA. The preceding components mentioned in CRISPR kit are listed in Table S4.

### Selection of target sequences

Target sites were selected using the CRISPR-P 2.0 design tool (http://cbi.hzau.edu.cn/CRISPR2/)^44^. We think that the target site should be located at the 5′ end of the gene’s ORF (open reading frame) to inactivate the entire gene. The transcription initiation sites of the sgRNAs with target sequences (target-sgRNAs) differ according to which the promoter is driving the expression. Adenine is the first nucleotide transcribed from AtU3 promoters and guanine is the first from AtU6 promoters^45,46^. Target site sequences in the genome are generally selected to be 5′-AN (19) NGG for AtU3 promoters and 5′-GN (19) NGG for AtU6 promoters^21^. Those are regular sites. In contrast, the irregular targets are that the base at the 5′ end of the target site is not identical to the base of the transcription start site of the AtU3/AtU6 promoter it uses.

### Cloning of single CRISPR target site in pHNCas9HT and transformation in competent Agrobacterium cells

Primer design for pHNCas9HT was determined using our primer design aid (Excel S1). A pair of primers for this protocol include X-Cas9-F and X-Cas9-R in this gadget. Reaction for primer annealing is as follows: X-Cas9-F (10 µM) 5 µl, X-Cas9-R (10 µM) 5 µl, 10X T4 DNA Ligase Buffer 2 µl (Thermo, USA), and nuclease-free water 8 µl. For precise annealing, the primers were incubated in a water bath for 5 min at 95 °C to 99 °C and allowed to cool down to room temperature naturally. The following reaction was established for ligation of target site DNA and Cas9VLHT. Cas9VLHT DNA 100 ng, right amount of target site DNA (the molar ratio of target site DNA to vector is5-20:1), 10X T4 DNA Ligase Buffer 1 µl, T4 DNA Ligase 0.5 µl (2.5 U), add nuclease-free water up to 10 µl, and incubate in water or metal bath at 22 °C for 60 min. Transforming ligation reactions directly into *A. tumefaciens* presents the following steps. All ligation mixture was added to 100 ul of fresh (first freeze–thaw) *A. tumefaciens* competent cells (GV3101, Weidi, China) and placed in an ice bath for 30 min and then in liquid nitrogen for 5 min. The cells were rapidly placed in 37 °C water bath for 5 min and then in ice bath for 5 min. A total of 500–1000 ul antibiotic-free LB medium was then added to the cells. Next, the cells were cultured in 28 °C at 200 rpm for 5–6 h, centrifuged at 5000 rpm at room temperature for 5 min, plated on a suitable antibiotic LB medium plate (contains 25 µg/ml rifampicin and 50 µg/ml kanamycin), and incubated in 28 °C for 48 h. The illustration of the preceding process mentioned is shown in Fig. 2a.

### Generation of sgRNA expression cassettes with target sequences for multi-site editing

In the current study, AtU3b-sgRNA, AtU3d-sgRNA, AtU6-26-sgRNA, and AtU6-29-sgRNA mixed templates were used in one-step PCR. Gene-specific primer design for pHNCas9 was adopted using our primer design aid (Excel S1). The gene-specific primer for this protocol was named gene name-AtUX-F in this gadget. In Table S3, we specify the details for using the BsaI site primer. SgRNA expression cassettes were amplified in 50 µl with 0.01 µM gene-specific primer, 0.2 µM each of the corresponding *Bsa* I-site primer, and 1 µl of corresponding mixed template using TransStart FastPfu Fly DNA Polymerase (Transgene, China) for 45–50 cycles (Fig. 2c). The PCR products were purified using a gel Pure kit (Magen, China). The presumed sequences of sgRNA expression cassettes produced by one-step PCR are provided in Text S3.

### Cloning of multiple sgRNA expression cassettes in the CRISPR/Cas9 binary vectors

Multiple sgRNA expression cassettes mixtures (1−2 µg) in an equimolar ratio were digested in a 50-μl reaction with 1 µl of *Bsa* I-HF (NEB, USA). Digested products were purified using a PCR Pure kit (Magen, China).

Ligation reactions (10 μl) were set up with 1 × T4 DNA ligase buffer, 5 U of T4 DNA ligase (Thermo, USA), 50 ng of Cas9VL, and 20 molar ratios of each digested sgRNA expression cassette (mixture). Reactions were incubated at 22 °C for 1 h. For two to four sgRNA expression cassettes, 10 µl of the ligation mixture was transformed into 50 µl of chemically competent transT1 *E. coli* (Transgene, China). In cases with five or more sgRNA expression cassettes, 100 µl of chemically competent transT1 *E. coli* are required. A schematic diagram of the assembly of multiple sgRNA expression cassettes is shown in Fig. 2d.

We prepared eight pairs of *Bsa* I-site primers; therefore, eight different sgRNA expression cassettes can be assembled by one-step Golden Gate assembly that is compatible with the BioBrick Standard Assembly. Biobricks of four sgRNA expression cassettes can be amplified using Bb-F and Bb-R primers from four ligation products of sgRNA expression cassettes and assembled into Biobricks site of pHNCas9. This strategy is suitable for ligating more than eight sgRNA expression cassettes into pHNCas9. Bb-F and Bb-R primer are provided in Table S2.

### Cloning of BioBrick OE*AtMYB75* in the CRISPR/Cas9 binary vectors

PEASY–*AtMYB75* was digested with *Kpn* I and *Spe* I restriction enzyme (Thermo, USA). BioBrick OE*AtMYB75* fragments were separated by agarose gel electrophoresis and purified using gel Pure kit (Magen, China). pHNCas9 was digested by *Kpn* I and *Xba* I restriction enzyme (Thermo, USA) using PCR pure kit. Ligation reactions (10 μl) were set up with 1 × T4 DNA ligase buffer, 1 U of T4 DNA ligase (Thermo, USA), 50 ng of pHNCas9, and five molar ratios of BioBrick OE*AtMYB75*. Reactions were incubated at 22 °C for 1 h. A total of 5 µl of the ligation mixture was transformed into 25 µl of chemically competent transT1 *E. coli* (Transgene, China). The new CRISPR vector containing BioBrick OE*AtMYB75* was named pHNCas9:OE*AtMYB75* (Fig. 7a).

### Plant transformation and growth conditions

Micro-Tom tomato plants were grown at 28 °C under long-day conditions (16 h light/8 h dark). The CRISPR/Cas9 constructs were introduced into chemically competent *A. tumefaciens* strain GV3101. Transformation of Micro-Tom tomato was performed as previously described^47^.

### Mutation detection

Genomic DNA from transgenic tomato leaves was extracted using a genome extraction kit (CWBIO, China) to detect mutated sequences. Amplifications of genomic sequence containing the target site were conducted by PCR using the primer pairs provided in Table S6. The PCR products were purified directly using a gel Pure kit (Magen, China). The purified PCR product is directly used for sequencing for T0 plant. Purified PCR products were cloned into the pEASY-Blunt vector (Transgene, China) to observe different types of mutations in T1 plant. Then, monoclonal sequencing was performed using the M13F primer by the Tsingke Biotechnologies Company and analyzed manually.

### Off-target mutation detection

Genomic DNA was extracted from transgenic tomato leaves using a genome extraction kit (CWBIO, China). PCR amplifications were conducted using primer pairs off-1F and off-1R, off-2F and off-2R, off-3F and off-3R, off-4F, and off-4R for *SlGRAS8* CRISPR lines (Table S7). The PCR products were purified by PCR Pure kit (Magen, China). The PCR products were used for the T7 endonuclease I (T7EI, Takara, Japan) assay and monoclonal sequencing analysis. Monoclonal sequencing analysis is briefly described as follows: PCR products were cloned into the pEASY-Blunt vector (Transgene, China). Plasmids from 10 single colonies for each sample were sequenced using the off-1F, off-2F, off-3F, and off-4F. Sequencing was performed by the Tsingke Biotechnologies Company and analyzed manually. The potential off-target mutated sites and their off-target scores were estimated with the CRISPR-P 2.0 design tool (http://cbi.hzau.edu.cn/CRISPR2/).

### Reverse transcription quantitative PCR analysis

Total RNA was extracted from tomato leaves using a HiPure HP Plant RNA Mini Kit (Magen, China). Reverse transcription was performed using a PrimeScript RT reagent kit with gDNA Eraser (TaKaRa, Japan), and quantitative PCRs were conducted with target-specific primers (Table S8) using SYBR® Premix Ex TaqTM II (TaKaRa, Japan).

## Results

### Development of primer design aid

We used the “SUBSTITUTE”, “REVERSE”, “CONCATENATE”, “IF”, and “OR” functions to design a primer design aid in Microsoft Excel software (Excel S1) to facilitate the application of this system. We set password protection for the relevant code, that is, “hunan198504” to prevent the tool from being edited incorrectly. The software is divided into two parts for use with pHNCas9 or pHNCas9HT. The desired primer sequence and GC content, and sgRNA sequence containing a target site can be obtained by inputting a gene name and target sequence and other necessary information in the specified cells of this software. In addition, this tool can automatically judge whether the target site is a regular target or an irregular target based on the input information to perform the corresponding processing. Several examples are provided in the software to help researchers rapidly grasp its use.

### Cloning of single CRISPR target site in pHNCas9HT and transformation in competent Agrobacterium cells

A total of 15 genes (Figure S5) were selected to demonstrate the usage of pHNCas9HT (a single sgRNA was used for each of the 15 genes tested). The target site primers (Table S9) were designed with our primer design aid. A pair of primers were annealed and ligated with Cas9VLHT (Fig. 2a). The ligation mixture was directly transformed into *Agrobacterium* competent cells (GV3101, Weidi, China). Colony PCR detection was performed using primer pair pHN-JC-F (Table S1) and gene-specific X-Cas9-R (Table S9). The characteristic DNA band of a positive colony was 529 bp. We performed 150 colony PCRs for identification (Figure S5). The result shows that positive *Agrobacterium* colonies reached 98% (148/150). Three positive *Agrobacterium* colonies were sequenced for each gene, and all the results are correct (Figure S5). This result shows that this strategy is suitable for the construction of large-scale single-target CRISPR without several procedures, such as PCR and plasmid extraction. This strategy is also fast, easy, and cheap.

### Assembly of multiple sgRNA expression cassettes in the CRISPR/Cas9 binary vectors in one pot

We tested two to eight sgRNA expression cassettes assembled into pHNCas9 in one pot to verify pHNCas9′s ability to accept multiple sgRNAs. A total of eight genes (Table S10) were selected in this experiment. The target site primers (Table S10) were designed with our primer design aid. Following the rules for using the *Bsa* I site primer (Table S3), we amplified sgRNA expression cassettes for two to eight assembly experiments by one-step PCR (Table S11). We set six time gradients of 0, 1, 3, 6, 9, 12, and 24 h without Cas9VL to investigate the optimal ligation time. We performed agarose gel electrophoresis on the ligation products. The results show that the brightness of the target DNA band was stabilized after 1 h of the ligation reaction (Figure S7). This result implies that 1 h is sufficient to complete the assembly of multiple sgRNA expression cassettes. Therefore, the optimal ligation reaction time is believed to be 1 h. We then verified the feasibility of assembling two to eight sgRNA cassettes into Cas9VL within 1 h. We counted the number of colonies that can be obtained when two to eight sgRNA cassettes were assembled into Cas9VL (Figure S8). We also performed colony PCR validation on the colonies obtained in this experiment (23 monoclonal, each plate was randomly selected). The results show that the ligation reaction can be performed for 1 h to obtain sufficient positive clones (Figure S8). We randomly selected three positive clones for sequencing on each plate. Sequencing results (Text S8) are consistent with the predicted sequence. Primers used for colony PCR and sequencing are provided in Table S1.

### Single CRISPR target site editing in tomato using pHNCas9HT

We selected *SlEIN2, SlARF2B*, and *SlERFE1* related to the ethylene signaling pathway to test the effectiveness of pHNCas9HT in genome editing. We separately designed one target site for these genes (Table 1). The target site primers (Table S9) were designed with our primer design aid. According to the preceding method, pHNCas9HT:*SlEin2*, pHNCas9HT:*SlERFE1*, and pHNCas9HT:*SlARF2B*, which target *SlEin2, SlERFE1*, and *SlARF2B*, respectively, were obtained, and were transformed into Micro-Tom tomato. A total of 11 independent T0 lines were obtained for pHNCas9HT:*SlEin2*. Seven independent T0 lines were obtained for pHNCas9HT:*SlERFE1* and five independent T0 lines were gained for pHNCas9HT:*SlARF2B*. We detected genome editing of these T0 plants by direct sequencing of PCR products. Primers used to amplify the genome containing the corresponding target sites are provided in Table S6. Therefore, we identified three T0 lines (L3, L5, and L8), three T0 lines (L1, L4, and L7), and two T0 lines (L2 and L5) edited in the target site in *SlEIN2*, *SlERFE1*, and *SlARF2B* CRISPR lines, respectively. Sequencing result of mutant lines is shown in Figures S6b–d.

**Table 1:** Target sites of *SlEIN2*, *SlERFE1*, *SlARF2B*, *SlGRAS8*, and *SlACS2&4*

Gene name	Target site sequence	GC content	Editing efficiency	secondary structure of sgRNA
*SlEIN2* target	AGCCCGATTTGGGTTTGATT**TGG** regular site	45%	3/11	Fig. 5d
*SlERFE1* target	AAACGAGCTCGACCCTCTAC**AGG** regular site	55%	3/7	Fig. 5e
*SlARF2B* target	CTTGTGACAGTGCCATGTGA**AGG** irregular target	50%	2/5	Fig. 5f
*SlGRAS8* target 1	TGAAGTAAAAGGCAGCTCGT**TGG** irregular target	45%	182/182	Fig. 5a
*SlGRAS8* target 2	ACTGAGTCAGCGATTCGCTG**TGG** regular site	55%	182/182	Fig. 5b
*SlGRAS8* target 3	CAAATTAGAACCGTTGGTAG**CGG** irregular target	40%	126/182	Fig. 5c
SlACS2&4 target 1	GCTTTCCACCCATCAAAATA**TGG** irregular target	40%	0/9	Fig. 5g
SlACS2&4 target 2	GCCAACTTTCAAGATTATCA**TGG** irregular target	35%	6/9	Fig. 5h

### Multi-site editing in tomato using pHNCas9

Here we designed three target sites for SlGRAS8 (Table 1). The target site primers (Table S5) were designed with our primer design aid. AtU3b, AtU3d, and AtU6-29 promoters were used for the three target sites. The sgRNA expression cassettes were amplified by one-step PCR in one pot using our CRISPR kit. We prepared pHNCas9:SlGRAS8 construct using Golden Gate cloning strategies, and tomatoes were transformed with this construct.

We obtained five T0 lines for this construct. We analyzed T0 plants by direct sequencing of PCR products containing the targeted sites. Primer pairs *SlGRAS8*-1F and *SlGRAS8*-3R for PCR amplification and sequencing are provided in Table S6.

We identified three mutant lines (L1, L2, and L3) by PCR product sequencing (Figure S6a). Monoclonal sequencing was performed in 11 T1 plants from L1, L2, and L3 to investigate the type of mutation in tomato. Overall, 182 valid sequences were obtained. All 182 sequences (100%) had at least two mutations (Text S6). The mutation rate of targets 1 and 2 is 100% in 182 sequences (Table 1). Target 3 has a low mutation rate of 69% in 182 sequences (Table 1).

**Fig. 3:**
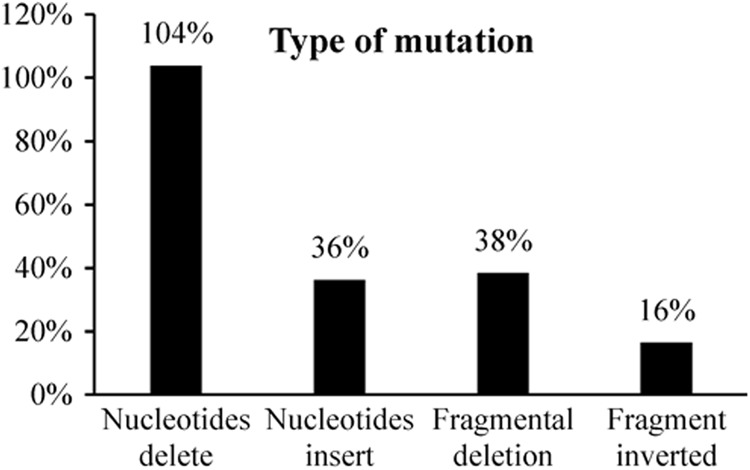
Type of mutations analysis in the *SlGRAS8* gene The proportions of each of four mutation types induced at target sites in the 182 sequences

We observed four types of targeted mutations: insertion of one nucleotide (A or T), deletion of a few nucleotides, deletion of a fragment between two sites, and inversions of a fragment between two sites. The proportions of each type of mutation at the three sites were calculated among the 182 known sequences (Fig. 3). The results show that a wide variety of mutation types are generated in tomato when contiguous multiple target sites are concatenated in pHNCas9.

The off-target mutation event rarely occurs when using CRISPR/Cas9 in higher plants and may be avoided by careful design of sgRNAs^10^. Nevertheless, by sequencing genomic PCR products and T7EI assay, we tested four potential off-target sites for the SlGRAS8 target, which had off-target score greater than 0.2 in T1 plant. All the PCR products of off-target sites showed negative T7EI digestion pattern (Figure S1). In addition, monoclonal sequencing results (10 clones from each sample and one sequencing result) show that none of the four potential off-target sites had undergone off-target editing event (Figure S2). We tested off-target sites1 with the highest off-target score (0.468) by T7EI assay and monoclonal sequencing in T4 plant to determine whether off-target events can accumulate in the offspring. The results show that no accumulation of the potential off-target event is observed in T4 plants (Figures S3 and S4).

**Fig. 4:**
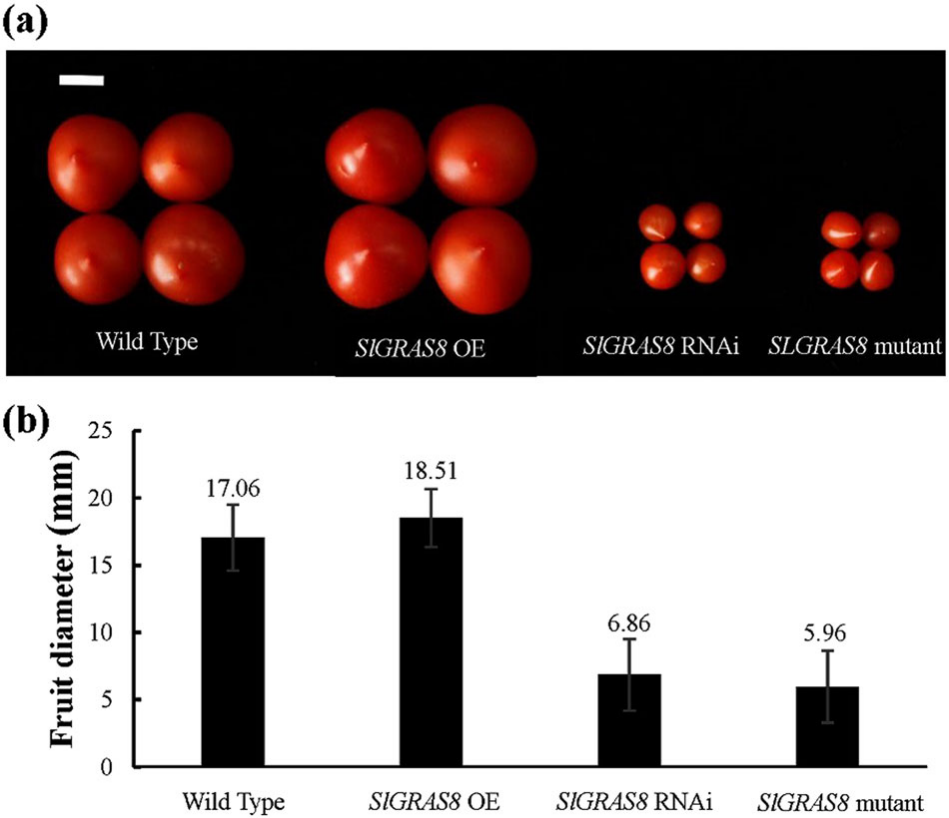
Partial phenotypes of fruit from genome-edited *SlGRAS8* plants. **a** Fruit from wild-type tomato and from plants in which *SlGRAS8* is overexpressed, silenced with RNAi, and genome edited. The phenotype of the mutant lines is consistent with that of the RNAi lines. Their fruit size is significantly smaller than the wild-type plant and *SlGRAS8* overexpression lines. **b** Fruit diameter statistics. *SlGRAS8* mutant and RNAi line’s fruit diameter are significantly smaller than wild-type plant and *SlGRAS8* overexpression lines.The length of the white scale bar is 1 cm

We observed the offspring of three independent mutants from T1 to T4 generation (Fig. 4 and Figure S9). The results show that the phenotype of the mutant is stable from the T1 to the T4 generation and consistent with our previously obtained RNAi Lines (unpublished). RNAi and mutant plants with disrupted *SlGRAS8* had smaller fruit than wild-type controls and *SlGRAS8* overexpression plant (unpublished) (Fig. 4). We sequenced their homozygous offspring’s genotypes. The results show that their genotypes are consistent (Figure S9). The above results indicate that the target site mutations can be stably inherited in the offspring.

The above results indicate that our CRISPR/Cas9 system can perform multiplex editing in tomato. Offspring has abundant mutation types and can stably inherit. Our system also has a low rate of off-target in tomato.

### Multi-gene editing in tomato using pHNCas9

We have demonstrated that single- and multi-site genome editing could be achieved using our CRISPR/Cas9 system. In this section, we selected two genes as the target genes of our system, *SlACS2* and *SlACS4*, which are both involved in ethylene synthesis during the ripening of fruit. Two target sites (Table 1) in the conserved region were also chosen due to the high homology between the two genes. Therefore, these target sites both target the two genes. The target site primers (Table S5) were designed with our primer design aid. AtU3b and AtU3d promoters were used for the two target sites. The sgRNA expression cassettes were amplified by one-step PCR in one pot using our CRISPR kit. We prepared pHNCas9:SlACS2&4 construct by adopting the Golden Gate cloning strategies and then transformed tomato with this construct.

We obtained nine T0 lines for these constructs. We performed direct PCR sequencing for nine T0 lines. Primer pairs (ACS2-F and ACS2-R) and (ACS4-F and ACS4-R) for PCR amplification and sequencing are provided in Table S6. Sequencing chromatograms are shown in Figures S6e–f. The results show that the *SlACS2* gene was mutated in L1, L2, L4, L6, L7, and L9, and the *SlACS4* gene was mutated in L2, L4, L6, and L9. However, only mutations in SlACS2&4 target 2 were detected. This result implies that the mutation rate caused by the SlACS2&4 target 1 is very low. The preceding findings proved that our CRISPR/Cas9 system can edit multiple genes in tomato.

### Secondary structure of sgRNA affects the mutation efficiency of target sites

In our study, we designed eight target sites (Table 1) for six genes, and our results show that their targeting efficiency is different. We attempted to find the factor that affects the sgRNA targeting efficiency from our data. First, GC content of sgRNA is reported to be important for the efficiency of CRISPR/Cas9 system^48^, and 97% of target site, which have been experimentally validated in plants, have a GC content between 30% and 80%^49^. The GC content of the eight target sites we used is between 35% and 55%.

**Fig. 5:**
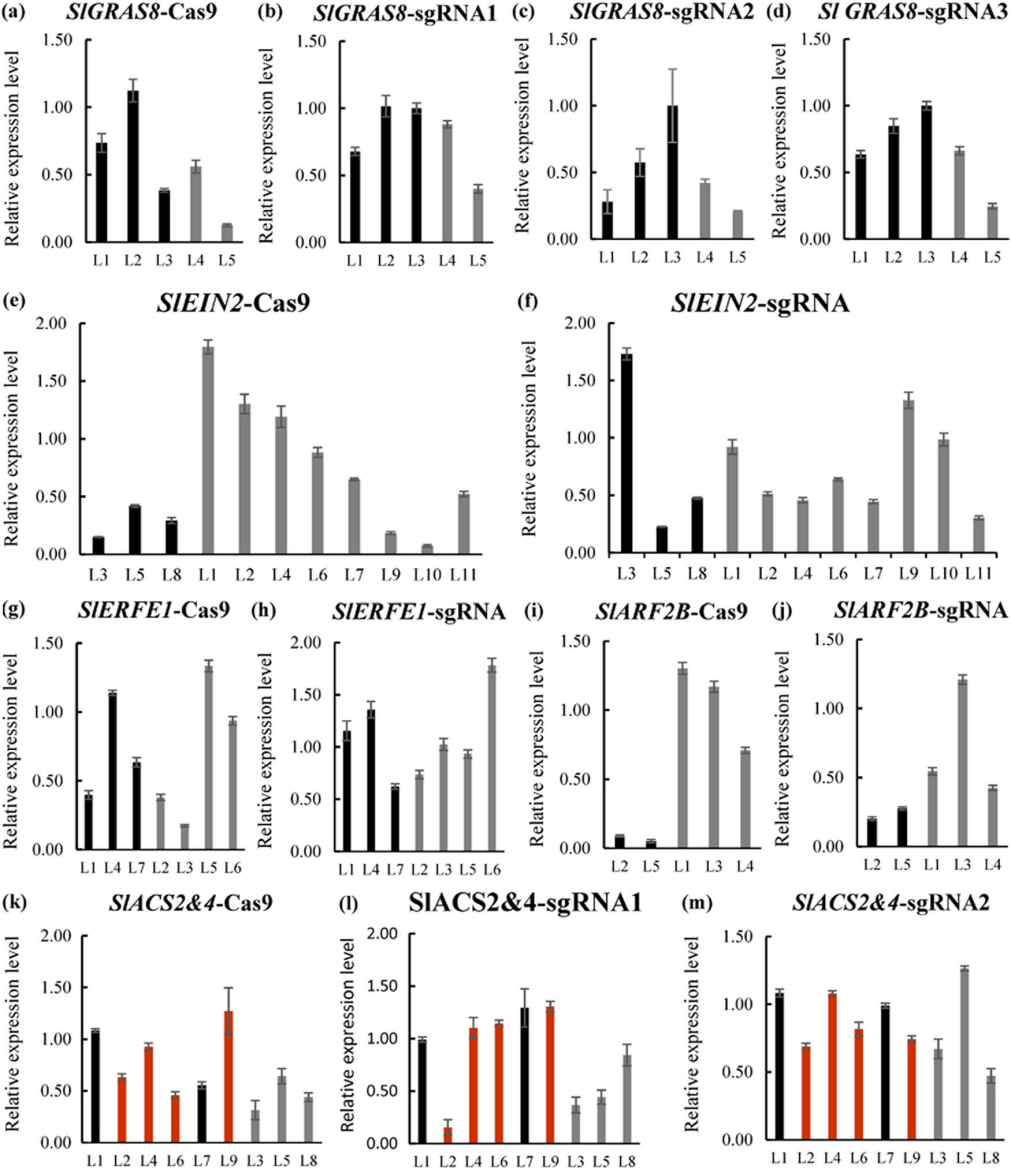
Relative expression level of Cas9 RNA and sgRNA in tomato. Expression of transgenic transcripts relative to endogenous *SlActin* transcripts analyzed by quantitative PCR. Relative expression levels are shown for **a** *Cas9* driven by 35S promoter in *SlGRAS8* CRISPR lines, **b** *SlGRAS8*-sgRNA1 driven by the AtU3b promoter. **c** *SlGRAS8*-sgRNA2 driven by the AtU3d promoter. **d** *SlGRAS8*-sgRNA3 driven by AtU6-29 promoter. **e** *Cas9* driven by 35S promoter in of *SlEIN2* CRISPR lines. **f** *SlEIN2*-sgRNA driven by the AtU3b promoter. **g** *Cas9* driven by 35S promoter in of *SlERF.E1* CRISPR lines. **h** *SlERF.E1*-sgRNA driven by the AtU3b promoter. **i** *Cas9* driven by 35S promoter in of *SlARF2B* CRISPR lines. **j** *SlARF2B*-sgRNA driven by the AtU3b promoter. **k** *Cas9* driven by 35S promoter in of *SlACS2&4* CRISPR lines. **l** *SlACS2&4*-sgRNA1 driven by the AtU3b promoter. **m** *SlACS2&4*-sgRNA2 driven by the AtU3b promoter. Gray columns correspond to lines that have not detected mutations. Black columns correspond to lines that with mutations. Red columns correspond lines with double gene mutation

We used reverse transcription fluorescence-quantitative PCR (RT–qPCR) to measure the expression level of *Cas9* and sgRNAs in all 37 lines obtained from the five constructs (Fig. 5). The q-PCR primers involved in this study are provided in Table S8. Gray columns correspond to lines that have no detected mutations. The corresponding lines in the black columns indicate the detected mutation. Double gene mutation was detected in the corresponding lines of the red columns. The results show that the expression levels of *Cas9* driven by the 35S promoter and sgRNA differed greatly among different lines (Fig. 5). Moreover, there is no positive correlation between mutation efficiency and the expression level of Cas9 and sgRNA in tomato. Hence, we speculate that the expression level of target sgRNA and Cas9 might not be the limiting factor for genome editing in tomato.

**Fig. 6:**
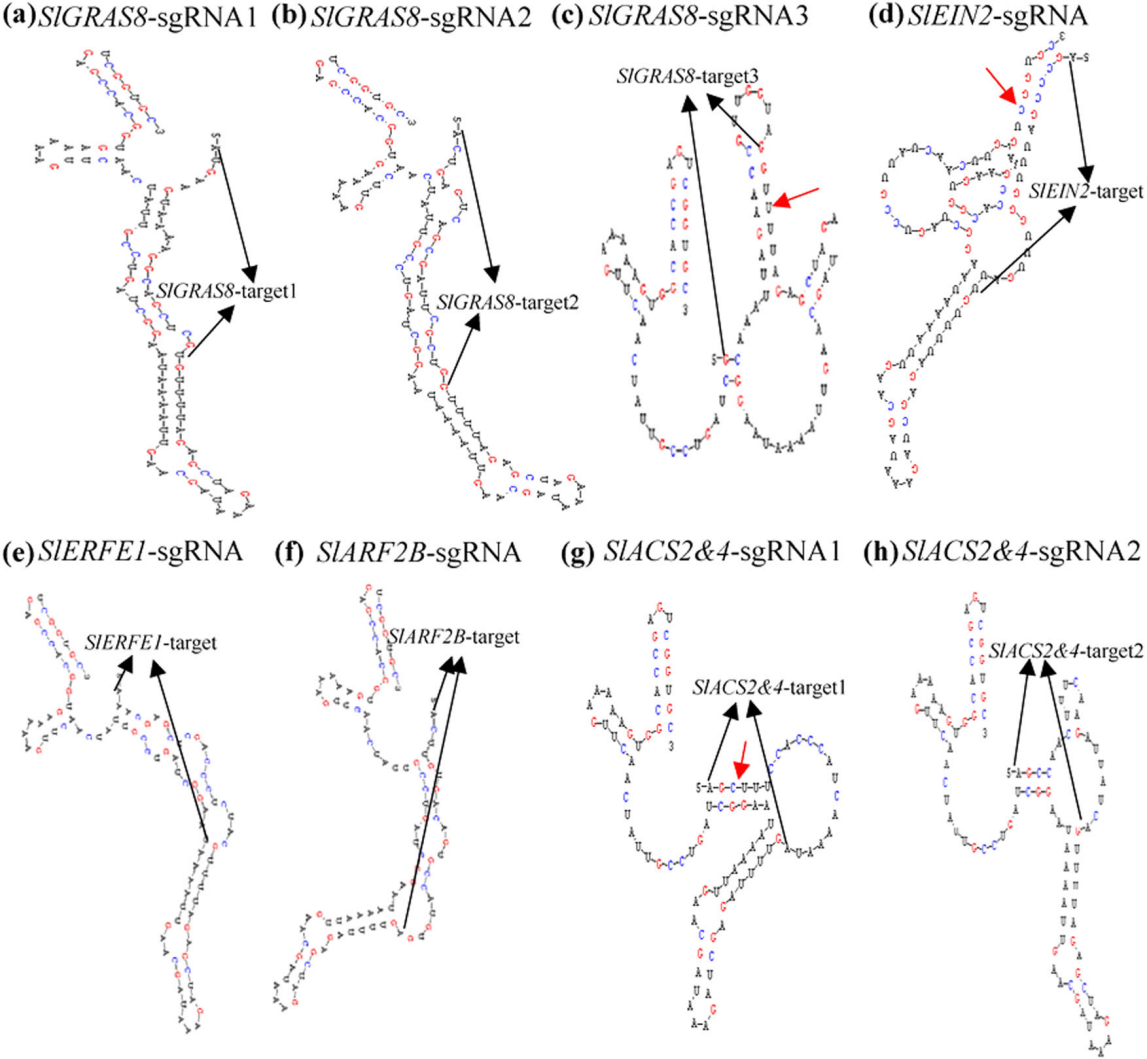
Secondary structures of all sgRNA transcripts The secondary structures were analyzed using DNAMAN soft. The sequence between the black arrows is the target site. Target pairing with sgRNA sequence 6 or more bp continuouly complementary is indicated by red arrows. The sgRNA sequences generated by our primer design aid. **a** The secondary structures of *SlGRAS8*-sgRNA1. **b** The secondary structures of *SlGRAS8*-sgRNA2. **c** The secondary structures of *SlGRAS8*-sgRNA3. **d** The secondary structures of *SlEIN2*-sgRNA. **e** The secondary structures of *SlERFE1*-sgRNA. **f** The secondary structures of *SlARF2B*-sgRNA. **g** The secondary structures of *SlACS2&4*-sgRNA1. **h** The secondary structures of *SlACS2&4*-sgRNA2

Studies suggested that the secondary structure of sgRNA may interfere with the editing efficiency^49,50^. If target sequences with six or more bp continuous pairing to the sgRNA, then editing efficiency will be considerably reduced in rice^21^. We predicted the secondary structure of the eight sgRNAs containing target sites using DNAMAN software in this study (Fig. 6). These sgRNA sequences are provided by our primer design aid. The results show that six or more bp continuously complementary between the target site with sgRNA backbones exist in the secondary structure of the three sgRNAs (Figs 6c–g) with lower mutation (Table 1) rate. Our results are consistent with Ma et al.^21^. The secondary structures of sgRNAs (containing target sites) are critical for Cas9/sgRNA effectiveness. Therefore, selection of target sequences should avoid those with pairing to the sgRNA by more than continuous 6 bp. Notably, the provided primer design aid can automatically present sgRNA sequences containing target sites. This sequence can be directly used for the prediction of secondary structure of sgRNAs.

### Preliminary functional verification of OE*AtMYB75* BioBrick

**Fig. 7:**
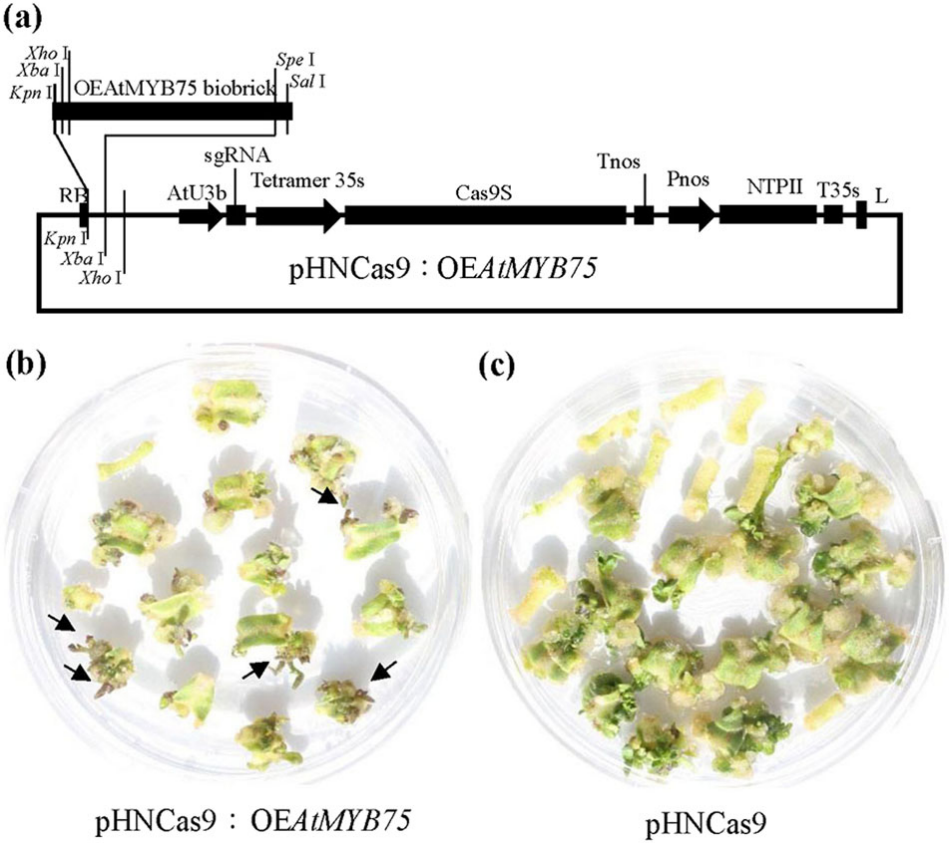
Function of OE*AtMYB75* BioBrick **a** Assembly of OEAtMYB75 BioBrick with pHNCas9. **b** The pHNCas9:OEAtMYB75 transformants are light purple when transferred to regeneration medium for 30 days. **c** The pHNCas9 transformants are green when transferred to regeneration medium for 30 days

The pHNCas9:OEAtMYB75 and pHNCas9 were transferred into Micro-Tom tomato through an Agrobacterium-mediated transformation method. The result shows that the pHNCas9:OE*AtMYB75* transformant is light purple (Fig. 7b) and pHNCas9 is green (Fig. 7c). This finding shows that the visual tag functions and potential can be used for positive or negative selection of the T-DNA insert.

## Discussion

We have developed an efficient, user-friendly, potentially high-throughput, open-source CRISPR/Cas9 system optimized for targeting multiple genes and genomic sites in tomato. Our system provides two binary vectors pHNCas9 and pHNCas9HT, five different Pol III-dependent promoters and sgRNA in pEASY clone vector, eight pairs *Bsa* I-site primer, and a gene-specific primer design aid. Our system is suitable for the preparation of a CRISPR kit. pHNCas9HT is suitable for rapid and low-cost generation of large-scale constructs for single-site editing without a considerable amount of work. The pHNCas9 was constructed to allow the assembly of up to eight sgRNA expression cassettes following the principles of one-pot Golden Gate cloning. Researchers can quickly design specific primers for pHNCas9HT or pHNCas9 by our primer design aid. Enriching the functionality of our CRISPR/Cas9 system is easy due to its compatibility. For example, the binary vector can accept additional multiple sgRNA expression cassette groups (up to four sgRNA expression cassettes can be contained), which can be created into a BioBrick by amplification using primers Bb-F and Bb-R.

We show that our system can perform single- and multi-site editing and multi-gene editing in tomato. However, significant differences in mutation efficiency exist between different sgRNAs (containing target site) and different lines. This may be related to the following factors. First GC content of target site is reported to be important for the editing efficiency^48^, and 97% of target site which have been experimentally validated in plants have a GC content between 30 and 80%^49^. Second, the effect of regular site and irregular site for genome editing efficiency was weak as reported in previous reports. In our work, 8 target sites we selected included regular sites and irregular sites. Our results indicate that the sites with a higher mutation rate (*SlGRAS8* target 1, *SlGRAS8* target 2, *SlACS2&4* target 2) contain the regular target and the irregular target. This implies that regular site and irregular site have less impact on editing efficiency. This conclusion is consistent with previous reports^21^. Third, it has been reported that the expression levels of Cas9 and sgRNA have an effect on editing efficiency^21^. We tested the expression levels of Cas9 and sgRNA in 37 lines. Our results suggest that there is no regular relationship between mutation efficiency and the expression level of Cas9 and sgRNA in tomato. However, we do not deny that a significant increase in the expression levels of Cas9 and sgRNA can potentially increase the editing efficiency of the CRISPR system in plants. This may depend on the new highly efficient promoters. At last, we found that the secondary structure of sgRNA had a greater influence on editing efficiency. In particular, the editing efficiencies of *SlGRAS8*-target3 and *slACS2&4*-target1 was significantly lower than other target sites in the same construct. This implies that the secondary structure of the sgRNA (containing the target site) is important for editing efficiency. This conclusion is consistent with the previous reports^21,42,49^. In addition, the secondary structure, which cannot be directly observed from the sequence of the target site, may be more easily overlooked by researchers. So we recommend that researchers should select several candidate target sites from the website (http://cbi.hzau.edu.cn/CRISPR2/) and predict secondary structure of candidate sgRNA using the sgRNA sequence from our primer design aid. The target site selected in this way may be more effective. Therefore, we propose performing a secondary structure prediction using the sgRNA sequence given by our primer design aid (Excel S1). Therefore, it is desirable to select the target site with about 30–80% GC content and continuously pairing with sgRNA backbone less than 6 bases.

PCR is presently used to identify positive T0 plant and the subsequent elimination of T1 plants containing transgenes. PCR is time consuming and laborious, especially when many plants in breeding populations must be screened. Hai-Ping Lu et al. (2017)^51^ down-regulated *CYP81A6* gene in rice through a CRISPR system containing *CYP81A6* RNAi expression cassettes to solve the problem in rice breeding. Rice in which *CYP81A6* is down-regulated is susceptible to bentazon. Researchers can use bentazon to screen for transgenic rice. However, the above scheme may be not apply to other crops. In fact for faster and high-throughput CRISPR methods for plant genome editing, the bottleneck is plant transformation and regeneration. To solve this problem, we think it is important to screen out explants that may regenerate positive plants from a large number of explants as soon as possible. So we proposed a visual T-DNA tag BioBrick in tomato. In our visual tag, *AtMYB75*/*PAP1* was driven by 35S promoter. Our preliminary results show that purple bud appear on explants in about 30 days using pHNCas9:OE*AtMYB75* and explant only need to be transferred one time during regeneration. Hence, the development of a visual CRISPR system is conducive to solve the bottleneck of high-throughput CRISPR in plant. In addition, researchers can directly select purple transgenic or green transgene-free plants in the offspring.

## Electronic supplementary material

Supporting Information

Excel S1
