# Supplementary material for: Rapid and user-friendly open-source CRISPR/Cas9 system for single- or multi-site editing of tomato genome
Source: Hortic Res. 2019 Jan 1;6:7. doi: 10.1038/s41438-018-0082-6 (PMC6312546; doi:10.1038/s41438-018-0082-6)
Supplement: Supplementary file 1 — Supporting Information [file 41438_2018_82_MOESM1_ESM.docx]

Supplementary information

**R****apid and user-friendly** **open-source CRISPR/Cas9 system for** **single- or multi-site editing of tomato genome**

| Contents |  |
| --- | --- |
| Text S1 | Sequence of *Cas9* |
| Text S2 | Sequences of the sgRNA and AtU3s/AtU6s in the plasmid |
| Text S3 | Presumed sequence of sgRNA expression cassette produced by one-step PCR |
| Text S4 | [Partial](javascript:void(0);) sequences of the pHNCas9HT |
| Text S5 | Partial sequences of peasy-OE*AtMYB75* |
| Text S6 | 182 valid sequences were obtained by monoclonal sequencing |
| TextS7 | Monoclonal sequencing for assembly of multiple sgRNA expression cassettes |
| Figure S1 | Off-target detection by T7EI assay in T1 plant |
| Figure S2 | Off-target detection by sequencing in T1 plant |
| Figure S3 | Off-target detection by T7EI assay in T4 plant |
| Figure S4 | Off-target detection by sequencing in T4 plant |
| Figure S5 | Transforming ligation reactions directly into *A. tumefaciens* |
| Figure S6 | Mutation efficiency analysis by direct sequencing of PCR products |
| Figure S7 | Assembly of Multiple sgRNA expression cassette |
| Figure S8 | Colony PCR for assembly of multiple sgRNA expression cassette |
| Figure S9 | Partial phenotypes and gene sequences of T4 generation *SlGRAS8* mutants |
| Table S1 | Primers used for PCR of AtU3s/AtU6s and sgRNA |
| Table S2 | Bsa I-site Primers used for one-step PCR of sgRNA expression cassettes |
| Table S3 | Rules of using Bsa I site primer |
| Table S4 | Components of CRISPR kit |
| Table S5 | Primers used for introduction of target sequences into sgRNA expression cassettes by one-step PCR |
| Table S6 | Primer for amplifing and sequencing of genomic fragment that contain editing sites |
| Table S7 | Primers for amplification of potential off target sites |
| Table S8 | Primers used for expression analysis |
| Table S9 | Primers for transforming ligation reactions product directly into A. tumefaciens |
| Table S10 | Primers for multiple sgRNA expression cassette assembly |
| Table S11 | The sgRNA expression cassettes for multiple assembly in one-pot |

## Text S1. Sequence of *Cas9*.

**Sequence of Cas9S**

The nucleotides encoding of the nuclear localization signals are shown in blue

1 ATGGCCCCAA AGAAAAAGAG AAAGGTTGAT TACAAAGACC ACGACGGAGA CTACAAAGAC

61 CACGACATTG ATTATAAAGA TGATGATGAT AAAGGAACGA TGGACAAAAA GTATAGCATC

121 GGTCTGGATA TTGGAACTAA CTCCGTCGGC TGGGCTGTAA TCACCGACGA ATACAAGGTC

181 CCGTCAAAAA AGTTCAAGGT ATTGGGTAAC ACAGATCGTC ACTCTATCAA AAAGAATCTC

241 ATTGGAGCTC TGTTGTTCGA CAGCGGCGAA ACAGCTGAGG CCACTAGACT GAAGCGCACC

301 GCCAGACGCC GTTACACGAG GAGAAAGAAC AGAATCTGCT ACTTGCAAGA AATATTCTCA

361 AACGAGATGG CCAAAGTGGA CGATTCGTTC TTTCATAGGT TAGAAGAGAG TTTCCTTGTT

421 GAAGAGGATA AAAAGCACGA AAGACATCCG ATATTTGGAA ACATCGTGGA CGAAGTTGCT

481 TATCACGAGA AGTACCCCAC GATCTATCAT CTGCGTAAAA AGTTGGTGGA CTCGACAGAT

541 AAGGCCGACC TCAGGTTAAT ATACCTTGCA CTGGCGCACA TGATCAAATT CAGAGGCCAT

601 TTTCTGATTG AAGGTGACCT GAACCCTGAC AATAGTGATG TGGACAAACT CTTCATTCAA

661 TTAGTTCAGA CCTACAATCA ACTGTTTGAA GAGAACCCTA TCAACGCTTC AGGAGTTGAC

721 GCTAAGGCCA TCCTTAGTGC GAGACTGAGC AAATCCCGCC GTCTTGAAAA CTTAATCGCA

781 CAGTTGCCTG GAGAGAAAAA GAACGGTTTG TTCGGAAATC TCATTGCGTT GTCACTCGGA

841 CTCACGCCAA ACTTCAAGTC TAACTTCGAT TTGGCAGAAG ACGCGAAACT GCAACTGAGC

901 AAAGACACAT ATGACGATGA CCTCGATAAC CTCTTAGCTC AGATCGGCGA TCAATACGCC

961 GACTTGTTCC TCGCTGCCAA AAATCTGTCG GACGCTATAC TTCTGAGTGA TATCTTGCGC

1021 GTCAACACAG AAATTACTAA GGCTCCTCTG TCGGCCAGTA TGATAAAACG CTATGACGAA

1081 CACCATCAGG ATTTGACATT GCTCAAAGCC CTCGTGCGTC AACAGCTCCC AGAAAAGTAC

1141 AAGGAGATTT TCTTTGATCA GTCCAAGAAT GGCTACGCAG GTTATATAGA CGGTGGAGCG

1201 TCGCAAGAAG AGTTCTACAA GTTCATCAAG CCAATATTAG AAAAGATGGA CGGCACGGAA

1261 GAGTTACTTG TTAAGCTGAA TCGTGAGGAC CTGTTGCGTA AACAGAGGAC ATTCGATAAC

1321 GGATCAATTC CGCACCAAAT ACATCTTGGC GAACTGCACG CTATCCTCAG GAGACAAGAG

1381 GACTTCTACC CCTTTTTAAA GGATAACCGT GAAAAGATCG AGAAAATCCT GACTTTCAGG

1441 ATTCCTTACT ATGTCGGCCC ACTGGCTCGT GGTAATAGCA GGTTTGCCTG GATGACCAGG

1501 AAGTCCGAAG AGACAATTAC TCCGTGGAAC TTCGAAGAGG TGGTTGATAA AGGAGCATCA

1561 GCGCAGTCTT TCATAGAACG CATGACAAAT TTTGACAAGA ACTTACCGAA TGAGAAGGTC

1621 CTTCCCAAAC ACTCACTCCT CTACGAATAC TTCACAGTAT ACAACGAGCT CACTAAAGTC

1681 AAGTACGTAA CCGAGGGTAT GCGCAAACCC GCTTTCCTGT CTGGAGAGCA GAAAAAGGCC

1741 ATCGTGGACC TTCTGTTCAA GACAAACCGT AAGGTCACTG TAAAGCAACT CAAGGAAGAC

1801 TACTTCAAAA AGATAGAGTG TTTCGATTCA GTGGAAATCT CTGGCGTTGA GGACAGATTT

1861 AACGCTTCCT TGGGTACTTA CCACGATTTG CTCAAGATCA TTAAAGATAA GGACTTCCTC

1921 GACAACGAAG AGAACGAAGA TATCTTAGAG GACATAGTTC TCACCCTTAC GCTGTTTGAA

1981 GATAGAGAGA TGATTGAAGA GCGCCTGAAG ACTTATGCTC ATTTGTTCGA TGACAAAGTC

2041 ATGAAGCAAC TGAAACGCCG TAGGTACACC GGCTGGGGTA GATTATCGCG CAAACTTATT

2101 AATGGTATAA GGGACAAGCA GTCGGGAAAA ACGATATTGG ACTTTCTCAA GAGTGATGGT

2161 TTCGCCAACA GAAATTTTAT GCAACTCATA CACGATGACA GCTTAACATT CAAGGAAGAT

2221 ATCCAAAAAG CACAGGTGTC GGGACAGGGC GACAGTTTGC ACGAACATAT TGCTAACCTC

2281 GCCGGCTCCC CGGCGATAAA AAAGGGTATC CTTCAGACTG TGAAAGTCGT AGATGAACTG

2341 GTGAAGGTTA TGGGTCGTCA TAAACCCGAG AACATAGTTA TCGAAATGGC TAGGGAGAAT

2401 CAAACAACTC AGAAGGGACA GAAAAACTCA AGAGAACGCA TGAAGCGCAT TGAAGAGGGT

2461 ATCAAAGAGC TTGGCAGTCA AATCCTGAAG GAACACCCTG TCGAGAACAC GCAACTTCAG

2521 AACGAAAAAT TGTACCTCTA CTATCTGCAG AATGGTAGAG ATATGTACGT AGACCAAGAA

2581 TTGGATATTA ACCGCCTCTC AGATTACGAC GTGGATCATA TAGTTCCGCA GTCATTCTTG

2641 AAGGATGACT CTATCGACAA CAAAGTCCTC ACAAGATCAG ACAAGAACCG CGGAAAATCA

2701 GATAATGTAC CCTCTGAAGA GGTGGTTAAA AAGATGAAAA ACTACTGGAG ACAGTTACTT

2761 AACGCTAAGT TGATCACGCA AAGAAAGTTC GATAACCTCA CAAAGGCTGA ACGCGGCGGT

2821 TTAAGCGAGC TTGACAAGGC CGGTTTCATA AAACGTCAGT TAGTCGAAAC CAGGCAAATT

2881 ACGAAACACG TAGCCCAAAT ATTGGATTCC CGCATGAACA CTAAATACGA TGAAAATGAC

2941 AAGCTCATCC GTGAGGTCAA AGTAATTACC CTGAAAAGCA AGTTGGTGTC CGACTTCAGA

3001 AAGGATTTCC AGTTCTACAA AGTTCGCGAA ATCAACAACT ACCACCATGC ACATGACGCT

3061 TACCTGAACG CAGTCGTAGG CACTGCGTTA ATTAAAAAGT ACCCTAAACT GGAATCTGAG

3121 TTCGTGTACG GTGACTATAA AGTGTACGAT GTTAGAAAGA TGATCGCTAA AAGCGAACAG

3181 GAGATTGGAA AGGCTACCGC CAAGTATTTC TTTTACTCCA ACATCATGAA TTTCTTTAAG

3241 ACCGAAATCA CGTTAGCAAA TGGCGAGATA CGTAAAAGGC CACTTATCGA AACAAACGGA

3301 GAAACTGGCG AGATAGTGTG GGACAAGGGT AGAGATTTTG CCACTGTCCG CAAAGTACTG

3361 TCGATGCCGC AAGTGAATAT CGTTAAAAAG ACCGAAGTTC AAACGGGAGG CTTCAGCAAA

3421 GAGTCCATCC TGCCCAAGCG TAACAGTGAT AAATTGATAG CTAGGAAAAA GGACTGGGAT

3481 CCTAAAAAGT ATGGTGGATT CGACAGCCCA ACTGTCGCAT ACTCCGTATT GGTGGTTGCG

3541 AAAGTCGAAA AAGGAAAGAG CAAAAAGCTC AAGTCCGTAA AAGAGCTGTT GGGCATTACC

3601 ATAATGGAAA GATCATCTTT CGAGAAGAAT CCTATCGATT TTCTGGAAGC CAAGGGATAT

3661 AAAGAGGTCA AAAAGGACCT CATAATCAAG TTACCAAAAT ACAGTCTGTT CGAATTGGAG

3721 AACGGCAGAA AACGCATGCT TGCATCAGCG GGTGAACTGC AAAAGGGAAA TGAGTTAGCA

3781 CTTCCTTCTA AATACGTCAA CTTCCTGTAT TTGGCGTCAC ACTACGAAAA ACTGAAGGGC

3841 TCTCCAGAAG ATAACGAGCA AAAGCAGTTA TTTGTGGAAC AGCACAAACA TTACCTTGAC

3901 GAAATTATAG AGCAAATCTC GGAGTTCAGT AAGAGAGTGA TTTTGGCTGA CGCCAATCTT

3961 GATAAAGTTC TGTCTGCTTA CAACAAGCAC CGTGATAAAC CGATTAGGGA ACAGGCCGAG

4021 AACATCATAC ATCTCTTCAC ACTCACTAAC CTTGGTGCAC CCGCAGCGTT CAAATATTTT

4081 GACACCACGA TAGATCGTAA GAGGTACACC AGCACGAAAG AAGTTTTGGA CGCGACACTC

4141 ATCCATCAAT CAATCACGGG CCTGTACGAG ACAAGAATCG ACCTGTCCCA GCTCGGTGGC

4201 GACTAG

## Text S2. Sequences of the sgRNA and AtU3s/AtU6s in the plasmid

AtU3/AtU6 promoter colored in green, Transcription initiation site written in **bold**, sgRNA colored in purple.

Sequences of AtU3b in the plasmid

CCTTTGGAATCGGCAGCAAAGGATTTACTTTAAATTTTTTCTTATGCAGCCTGTGATGGATAACTGAATCAAACAAATGGCGTCTGGGTTTAAGAAGATCTGTTTTGGCTATGTTGGACGAAACAAGTGAACTTTTAGGATCAACTTCAGTTTATATATGGAGCTTATATCGAGCAATAAGATAAGTGGGCTTTTTATGTAATTTAATGGGCTATCGTCCATAGATTCACTAATACCCATGCCCAGTACCCATGTATGCGTTTCATATAAGCTCCTAATTTCTCCCACATCGCTCAAATCTAAACAAATCTTGTTGTATATATAACACTGAGGGAGCAACATTGGTC**A**

Sequences of AtU3d in the plasmid

CCTTTGGAATCGGCAGCAAAGGAATAAGCTTATGATTTCTTTTTTCTTACGAATTTTGCGTCCCACATCGGTAAGCGAGTGAAGAAATAACTGCTTTATATATGGCTACAAAGCACCATTGGTC**A**

Sequences of AtU6-1 in the plasmid

CCCTTTGGAATCGGCAGCAAAGGAGAAATCTCAAAATTCCGGCAGAACAATTTTGAATCTCGATCCGTAGAAACGAGACGGTCATTGTTTTAGTTCCACCACGATTATATTTGAAATTTACGTGAGTGTGAGTGAGACTTGCATAAGAAAATAAAATCTTTAGTTGGGAAAAAATTCAATAATATAAATGGGCTTGAGAAGGAAGCGAGGGATAGGCCTTTTTCTAAAATAGGCCCATTTAAGCTATTAACAATCTTCAAAAGTACCACAGCGCTTAGGTAAAGAAAGCAGCTGAGTTTATATATGGTTAGAGACGAAGTAGTGATT**G**

Sequences of AtU6-26 in the plasmid

CCCTTTGGAATCGGCAGCAAAGGAAGCTTTCGTTTTCTTCTTTTTAACTTTCCATTCGGAGTTTTTGTATCTTGTTTCATAGTTTGTCCCAGGATTAGAATGATTAGGCATCGAACCTTCAAGAATTTGATTGAATAAAACATCTTCATTCTTAAGATATGAAGATAATCTTCAAAAGGCCCCTGGGAATCTGAAAGAAGAGAAGCAGGCCCATTTATATGGGAAAGAACAATAGTATTTCTTATATAGGCCCATTTAAGTTGAAAACAATCTTCAAAAGTCCCACATCGCTTAGATAAGAAAACGAAGCTGAGTTTATATACAGCTAGAGTCGAAGTAGTGATT**G**

Sequences of AtU6-29 in the plasmid

CCTTTGGAATCGGCAGCAAAGGAAAATATCAGAGATCTCTTACAGTTAGTTTCGTTCTTAATCCAAACTACTGCAGCCTGACAGACAAATGAGGATGCAAACAATTTTAAAGTTTATCTAACGCTAGCTGTTTTGTTTCTTCTCTCTGGTGCACCAACGACGGCGTTTTCTCAATCATAAAGAGGCTTGTTTTACTTAAGGCCAATAATGTTGATGGATCGAAAGAAGAGGGCTTTTAATAAACGAGCCCGTTTAAGCTGTAAACGATGTCAAAAACATCCCACATCGTTCAGTTGAAAATAGTAGCTCTGTTTATATATTGGTAGAGTCGACTAAGAGATT**G**

Sequences of sgRNA in the plasmid

GTTTTAGAGCTAGAAATAGCAAGTTAAAATAAGGCTAGTCCGTTATCAACTTGAAAAAGTGGCACCGAGTCGGTGCTTTTTTTCAAGAGCTTGGAGTGGATGGAAG

## Text S3. Presumed sequence of sgRNA expression cassette produced by one-step PCR.

*BsaI* site colored in red, *BsaI* restriction overhangs colored in blue, AtU3/AtU6 promoter colored in green, Transcription initiation site written in **bold**, target sequence written in **underlined**, sgRNA colored in purple.

**Presumed sequence of AtU3b-sgRNA** **expression cassette**

CGTGGGTCTCTNNNNCCTTTggaatcggcagcaaaggatttactttaaattttttcttatgcagcctgtgatggataactgaatcaaacaaatggcgtctgggtttaagaagatctgttttggctatgttggacgaaacaagtgaacttttaggatcaacttcagtttatatatggagcttatatcgagcaataagataagtgggctttttatgtaatttaatgggctatcgtccatagattcactaatacccatgcccagtacccatgtatgcgtttcatataagctcctaatttctcccacatcgctcaaatctaaacaaatcttgttgtatatataacactgagggagcaacattggtc**A(19-20bp target sequenc)**GTTTTAGAGCTAGAAATAGCAAGTTAAAATAAGGCTAGTCCGTTATCAACTTGAAAAAGTGGCACCGAGTCGGTGCTTTTTTTCAAGAGCTTGGAGTGGATGGAANNNNAGAGACCCACG

**Presumed sequence of AtU3d-sgRNA** **expression cassette**

CGTGGGTCTCTNNNNCCTTTGGAATCGGCAGCAAAGGAATAAGCTTATGATTTCTTTTTTCTTACGAATTTTGCGTCCCACATCGGTAAGCGAGTGAAGAAATAACTGCTTTATATATGGCTACAAAGCACCATTGGTC**A**(**19-20bp target sequenc)**GTTTTAGAGCTAGAAATAGCAAGTTAAAATAAGGCTAGTCCGTTATCAACTTGAAAAAGTGGCACCGAGTCGGTGCTTTTTTTCAAGAGCTTGGAGTGGATGGAANNNNAGAGACCCACG

**Presumed sequence of AtU6-1-sgRNA** **expression cassette**

CGTGGGTCTCTNNNNCCTTTGGAATCGGCAGCAAAGGAGAAATCTCAAAATTCCGGCAGAACAATTTTGAATCTCGATCCGTAGAAACGAGACGGTCATTGTTTTAGTTCCACCACGATTATATTTGAAATTTACGTGAGTGTGAGTGAGACTTGCATAAGAAAATAAAATCTTTAGTTGGGAAAAAATTCAATAATATAAATGGGCTTGAGAAGGAAGCGAGGGATAGGCCTTTTTCTAAAATAGGCCCATTTAAGCTATTAACAATCTTCAAAAGTACCACAGCGCTTAGGTAAAGAAAGCAGCTGAGTTTATATATGGTTAGAGACGAAGTAGTGATT**G(19-20bp target sequenc)**GTTTTAGAGCTAGAAATAGCAAGTTAAAATAAGGCTAGTCCGTTATCAACTTGAAAAAGTGGCACCGAGTCGGTGCTTTTTTTCAAGAGCTTGGAGTGGATGGAANNNNAGAGACCCACG

**Presumed sequence of AtU6-26-sgRNA** **expression cassette**

CGTGGGTCTCTNNNNCCTTTGGAATCGGCAGCAAAGGAAGCTTTCGTTTTCTTCTTTTTAACTTTCCATTCGGAGTTTTTGTATCTTGTTTCATAGTTTGTCCCAGGATTAGAATGATTAGGCATCGAACCTTCAAGAATTTGATTGAATAAAACATCTTCATTCTTAAGATATGAAGATAATCTTCAAAAGGCCCCTGGGAATCTGAAAGAAGAGAAGCAGGCCCATTTATATGGGAAAGAACAATAGTATTTCTTATATAGGCCCATTTAAGTTGAAAACAATCTTCAAAAGTCCCACATCGCTTAGATAAGAAAACGAAGCTGAGTTTATATACAGCTAGAGTCGAAGTAGTGATT**G****(19-20bp target sequenc)**GTTTTAGAGCTAGAAATAGCAAGTTAAAATAAGGCTAGTCCGTTATCAACTTGAAAAAGTGGCACCGAGTCGGTGCTTTTTTTCAAGAGCTTGGAGTGGATGGAANNNNAGAGACCCACG

**Presumed sequence of AtU6-29-sgRNA expression cassette**

CGTGGGTCTCTNNNNCTTTGGAATCGGCAGCAAAGGAAAATATCAGAGATCTCTTACAGTTAGTTTCGTTCTTAATCCAAACTACTGCAGCCTGACAGACAAATGAGGATGCAAACAATTTTAAAGTTTATCTAACGCTAGCTGTTTTGTTTCTTCTCTCTGGTGCACCAACGACGGCGTTTTCTCAATCATAAAGAGGCTTGTTTTACTTAAGGCCAATAATGTTGATGGATCGAAAGAAGAGGGCTTTTAATAAACGAGCCCGTTTAAGCTGTAAACGATGTCAAAAACATCCCACATCGTTCAGTTGAAAATAGTAGCTCTGTTTATATATTGGTAGAGTCGACTAAGAGATT**G(19-20bp target sequenc)**GTTTTAGAGCTAGAAATAGCAAGTTAAAATAAGGCTAGTCCGTTATCAACTTGAAAAAGTGGCACCGAGTCGGTGCTTTTTTTCAAGAGCTTGGAGTGGATGGAANNNNAGAGACCCACG

## Text S4. [Partial](javascript:void(0);) sequences of the pHNCas9HT

1. **[Partial](javascript:void(0);) sequences of the pHNCa9HT**

AtU3/AtU6 promoter colored in green, sgRNA colored in purple, *Esp3* I site colored in red, *BsaI* restriction overhangs were writen in highlight.

aagctt**TGGTACC**CCG**TCTAGA**GG**CTCGAG**TAGCTCCTTTGGAATCGGCAGCAAAGGATTTACTTTAAATTTTTTCTTATGCAGCCTGTGATGGATAACTGAATCAAACAAATGGCGTCTGGGTTTAAGAAGATCTGTTTTGGCTATGTTGGACGAAACAAGTGAACTTTTAGGATCAACTTCAGTTTATATATGGAGCTTATATCGAGCAATAAGATAAGTGGGCTTTTTATGTAATTTAATGGGCTATCGTCCATAGATTCACTAATACCCATGCCCAGTACCCATGTATGCGTTTCATATAAGCTCCTAATTTCTCCCACATCGCTCAAATCTAAACAAATCTTGTTGTATATATAACACTGAGGGAGCAACATTGGTC**A**TGAGACGAAGCGTGCGTCTCGGTTTTAGAGCTAGAAATAGCAAGTTAAAATAAGGCTAGTCCGTTATCAACTTGAAAAAGTGGCACCGAGTCGGTGCTTTTTTT

## Text S5. [Partial](javascript:void(0);) sequences of peasy-OE*AtMYB75*

**GGTACC**, **TCTAGA**, and **CTCGAG** are *Kpn* I, *Xba* I and *Xho* I restriction sites. **GTCGAC** and **ACTAGT** are *Sal* I and *Spe* I restriction sites. The 35s promoter sequence was written on the underline. Nos terminator sequence was written by *underline*. *AtMYB75/PAP1* sequence was written by red.

**GGTACC**CCG**TCTAGA**GG**CTCGAG**CtgcattaatgaatcggccaacgcgcggggagaggcggtttgcgtattggctagagcagcttgccaacatggtggagcacgacactctcgtctactccaagaatatcaaagatacagtctcagaagaccaaagggctattgagacttttcaacaaagggtaatatcgggaaacctcctcggattccattgcccagctatctgtcacttcatcaaaaggacagtagaaaaggaaggtggcacctacaaatgccatcattgcgataaaggaaaggctatcgttcaagatgcctctgccgacagtggtcccaaagatggacccccacccacgaggagcatcgtggaaaaagaagacgttccaaccacgtcttcaaagcaagtggattgatgtgataacatggtggagcacgacactctcgtctactccaagaatatcaaagatacagtctcagaagaccaaagggctattgagacttttcaacaaagggtaatatcgggaaacctcctcggattccattgcccagctatctgtcacttcatcaaaaggacagtagaaaaggaaggtggcacctacaaatgccatcattgcgataaaggaaaggctatcgttcaagatgcctctgccgacagtggtcccaaagatggacccccacccacgaggagcatcgtggaaaaagaagacgttccaaccacgtcttcaaagcaagtggattgatgtgatatctccactgacgtaagggatgacgcacaatcccactatccttcgcaagaccttcctctatataaggaagttcatttcatttggagaggacacgctgaaatcaccagtctctctctacaaatctatctctGGATCCatggagggttcgtccaaagggctgcgaaaaggtgcttggactactgaagaagatagtctcttgagacagtgcattaataagtatggagaaggcaaatggcaccaagttcctgtaagagctgggctaaaccggtgcaggaaaagttgtagattaagatggttgaactatttgaagccaagtatcaagagaggaaaacttagctctgatgaagtcgatcttcttcttcgccttcataggcttctagggaataggtggtctttaattgctggaagattacctggtcggaccgcaaatgacgtcaagaattactggaacactcatctgagtaagaaacatgaaccgtgttgtaagataaagatgaaaaagagagacattacgcccattcctacaacaccggcactaaaaaacaatgtttataagcctcgacctcgatccttcacagttaacaacgactgcaaccatctcaatgccccaccaaaagttgacgttaatcctccatgccttggacttaacatcaataatgtttgtgacaatagtatcatatacaacaaagataagaagaaagaccaACTTGTgaataatttgattgatggagataatatgtggttagagaaattcctagaggaaagccaagaggtagatattttggttcctgaagcgacgacaacagaaaagggggacaccttggcttttgacgttgatcaactttggagtcttttcgatggagagactgtgaaatttgattagtgtttcgaacatttgtttgcgACTAGAttccccgatcgttcaaacatttggcaataaagtttcttaagattgaatcctgttgccggtcttgcgatgattatcatataatttctgttgaattacgttaagcatgtaataattaacatgtaatgcatgacgttatttatgagatgggtttttatgattagagtcccgcaattatacatttaatacgcgatagaaaacaaaatatagcgcgcaaactaggataaattatcgcgcgcggtgtcatctatgttactagatcggg**ACTAGT**GCTTGTTCGGTC**GTCGAC**

## Text S6. 182 valid sequences were obtained by monoclonal sequencing

Target site colored in black, target site flanking sequence colored in red, wild-type genome sequence in the first row, the sequence between the target sites is replaced by “NNNN”, unedited target sequence written in underlined, the missing sequence is represented by a space, Inserted sequence in **bold,** Inverted sequence colored in green.

U3b-*SlGRAS8*-site1 U3d-*SlGRAS8*-site2 U6-29-*SlGRAS8*-site3

**WT** GCTTGAAGTGCTTCCTTGAAGTAAAAGGCAGCTCGTTGGAGAGGTTTNNNNATTATGGAACTGAGTCAGCGATTCGCTGTGGGTGAGAGGNNNNTCGTAGCACAAATTAGAACCGTTGGTAGCGGCTGGAAAAGTAAAGC

**L1-1**  (heterozygous)

- - 1. GCTTGAAGTGCTTCCTTGAAGTAAA CGTTGGAGAGGTTTNNNNATTATGGAACTGAGTCAGCGATTCC TGTGGGTGAGAGGNNNNTCGTAGCACAAATTAGAACCGTTGGTAGCGGCTGGAAAAGTAAAGC
    2. GCTTGAAGTGCTTCCTTGAAGTAAA CGTTGGAGAGGTTTNNNNATTATGGAACTGAGTCAGCGATTCC TGTGGGTGAGAGGNNNNTCGTAGCACAAATTAGAACCGTTGGTAGCGGCTGGAAAAGTAAAGC
    3. GCTTGAAGTGCTTCCTTGAAGTAAA CGTTGGAGAGGTTTNNNNATTATGGAACTGAGTCAGCGATTCC TGTGGGTGAGAGGNNNNTCGTAGCACAAATTAGAACCGTTGGTAGCGGCTGGAAAAGTAAAGC
    4. GCTTGAAGTGCTTCCTTGAAGTAAA CGTTGGAGAGGTTTNNNNATTATGGAACTGAGTCAGCGATTCC TGTGGGTGAGAGGNNNNTCGTAGCACAAATTAGAACCGTTGGTAGCGGCTGGAAAAGTAAAGC
    5. GCTTGAAGTGCTTCCTTGAAGTAAAAGGCAGCT TAGCGGCTGGAAAAGTAAAGC
    6. GCTTGAAGTGCTTCCTTGAAGTAAAAGGCAGCT TAGCGGCTGGAAAAGTAAAGC
    7. GCTTGAAGTGCTTCCTTGAAGTAAAAGGCAGCT TAGCGGCTGGAAAAGTAAAGC
    8. GCTTGAAGTGCTTCCTTGAAGTAAAAGGCAGCT TAGCGGCTGGAAAAGTAAAGC
    9. GCTTGAAGTGCTTCCTTGAAGTAAAAGGCAGCT TAGCGGCTGGAAAAGTAAAGC
    10. GCTTGAAGTGCTTCCTTGAAGTAAAAGGCAGCT TAGCGGCTGGAAAAGTAAAGC
    11. GCTTGAAGTGCTTCCTTGAAGTAAAAGGCAGCT TAGCGGCTGGAAAAGTAAAGC
    12. GCTTGAAGTGCTTCCTTGAAGTAAAAGGCAGCT TAGCGGCTGGAAAAGTAAAGC
    13. GCTTGAAGTGCTTCCTTGAAGTAAAAGGCAGCT TAGCGGCTGGAAAAGTAAAGC
    14. GCTTGAAGTGCTTCCTTGAAGTAAAAGGCAGCT TAGCGGCTGGAAAAGTAAAGC
    15. GCTTGAAGTGCTTCCTTGAAGTAAAAGGCAGCT TAGCGGCTGGAAAAGTAAAGC
    16. GCTTGAAGTGCTTCCTTGAAGTAAAAGGCAGCT TAGCGGCTGGAAAAGTAAAGC
    17. GCTTGAAGTGCTTCCTTGAAGTAAAAGGCAGCT TAGCGGCTGGAAAAGTAAAGC
    18. GCTTGAAGTGCTTCCTTGAAGTAAAAGGCAGCT TAGCGGCTGGAAAAGTAAAGC
    19. GCTTGAAGTGCTTCCTTGAAGTAAAAGGCAGCT TAGCGGCTGGAAAAGTAAAGC

U3b-*SlGRAS8*-site1 U3d-*SlGRAS8*-site2 U6-29-*SlGRAS8*-site3

**WT** GCTTGAAGTGCTTCCTTGAAGTAAAAGGCAGCTCGTTGGAGAGGTTTNNNNATTATGGAACTGAGTCAGCGATTCGCTGTGGGTGAGAGGNNNNTCGTAGCACAAATTAGAACCGTTGGTAGCGGCTGGAAAAGTAAAGC

**L1-2** (heterozygous)

1. GCTTGAAGTGCTTCCTTGAAGTAAAAGGCAGCTC TGTGGGTGAGAGGNNNNTCGTAGCACAAATTAGAACCGTT TAGCGGCTGGAAAAGTAAAGC
2. GCTTGAAGTGCTTCCTTGAAGTAAAAGGCAGCTC TGTGGGTGAGAGGNNNNTCGTAGCACAAATTAGAACCGTT TAGCGGCTGGAAAAGTAAAGC
3. GCTTGAAGTGCTTCCTTGAAGTAAAAGGCAGCTC TGTGGGTGAGAGGNNNNTCGTAGCACAAATTAGAACCGTT TAGCGGCTGGAAAAGTAAAGC
4. GCTTGAAGTGCTTCCTTGAAGTAAAAGGCAGCTC TGTGGGTGAGAGGNNNNTCGTAGCACAAATTAGAACCGTT TAGCGGCTGGAAAAGTAAAGC
5. GCTTGAAGTGCTTCCTTGAAGTAAAAGGCAGCTC TGTGGGTGAGAGGNNNNTCGTAGCACAAATTAGAACCGTT TAGCGGCTGGAAAAGTAAAGC
6. GCTTGAAGTGCTTCCTTGAAGTAAAAGGCAGCTC TGTGGGTGAGAGGNNNNTCGTAGCACAAATTAGAACCGTT TAGCGGCTGGAAAAGTAAAGC
7. GCTTGAAGTGCTTCCTTGAAGTAAAAGGCAGCTC TGTGGGTGAGAGGNNNNTCGTAGCACAAATTAGAACCGTT TAGCGGCTGGAAAAGTAAAGC
8. GCTTGAAGTGCTTCCTTGAAGTAAAAGGCAGCTC TGTGGGTGAGAGGNNNNTCGTAGCACAAATTAGAACC TAGCGGCTGGAAAAGTAAAGC
9. GCTTGAAGTGCTTCCTTGAAGTAAAAGGCAGCTC TGTGGGTGAGAGGNNNNTCGTAGCACAAATTAGAACC TAGCGGCTGGAAAAGTAAAGC
10. GCTTGAAGTGCTTCCTTGAAGTAAAAGGCAGCTC TGTGGGTGAGAGGNNNNTCGTAGCACAAATTAGAACC TAGCGGCTGGAAAAGTAAAGC
11. GCTTGAAGTGCTTCCTTGAAGTAAAAGGCAGCTC TGGGGTGAGAGGNNNN TCGTAGCACAAATTAGAACC TAGCGGCTGGAAAAGTAAAGC
12. GCTTGAAGTGCTTCCTTGAAGTAAAAGGCAGCTC TGTGGGTGAGAGGNNNNTCGTAGCACAAATTAGAACC TAGCGGCTGGAAAAGTAAAGC
13. GCTTGAAGTGCTTCCTTGAAGTAAAAGGCAGCTC TGTGGGTGAGAGGNNNNTCGTAGCACAAATTAGAACC TAGCGGCTGGAAAAGTAAAGC
14. GCTTGAAGTGCTTCCTTGAAGTAAAAGGCAGCTC TGTGGGTGAGAGGNNNNTCGTAGCACAAATTAGAACC TAGCGGCTGGAAAAGTAAAGC
15. GCTTGAAGTGCTTCCTTGAAGTAAAAGGCAGCTC TGTGGGTGAGAGGNNNNTCGTAGCACAAATTAGAACC TAGCGGCTGGAAAAGTAAAGC
16. GCTTGAAGTGCTTCCTTGAAGTAAAAGGCAGCTC TGTGGGTGAGAGGNNNNTCGTAGCACAAATTAGAACC TAGCGGCTGGAAAAGTAAAGC
17. GCTTGAAGTGCTTCCTTGAAGTAAAAGGCAGCTC TGTGGGTGAGAGGNNNNTCGTAGCACAAATTAGAACC TAGCGGCTGGAAAAGTAAAGC
18. GCTTGAAGTGCTTCCTTGAAGTAAAAGGCAGCTC TGTGGGTGAGAGGNNNNTCGTAGCACAAATTAGAACC TAGCGGCTGGAAAAGTAAAGC

U3b-*SlGRAS8*-site1 U3d-*SlGRAS8*-site2 U6-29-*SlGRAS8*-site3

**WT** GCTTGAAGTGCTTCCTTGAAGTAAAAGGCAGCTCGTTGGAGAGGTTTNNNNATTATGGAACTGAGTCAGCGATTCGCTGTGGGTGAGAGGNNNNTCGTAGCACAAATTAGAACCGTTGGTAGCGGCTGGAAAAGTAAAGC

**L1-3** (heterozygous)

- - 1. GCTTGAAGTGCTTCCTTGAAGTAAAAGGCA CGTTGGAGAGGTTTNNNNATTATGGAACTGAGTCAGCGATTCGT AGCGGCTGGAAAAGTAAAGC
    2. GCTTGAAGTGCTTCCTTGAAGTAAAAGGCA CGTTGGAGAGGTTTNNNNATTATGGAACTGAGTCAGCGATTCGT AGCGGCTGGAAAAGTAAAGC
    3. GCTTGAAGTGCTTCCTTGAAGTAAAAGGCA CGTTGGAGAGGTTTNNNNATTATGGAACTGAGTCAGCGATTCGT AGCGGCTGGAAAAGTAAAGC
    4. GCTTGAAGTGCTTCCTTGAAGTAAAAGGCA CGTTGGAGAGGTTTNNNNATTATGGAACTGAGTCAGCGATTCGT AGCGGCTGGAAAAGTAAAGC
    5. GCTTGAAGTGCTTCCTTGAAGTAAAAGGCA CGTTGGAGAGGTTTNNNNATTATGGAACTGAGTCAGCGATTCGT AGCGGCTGGAAAAGTAAAGC
    6. GCTTGAAGTGCTTCCTTGAAGTAAAAGGCA CGTTGGAGAGGTTTNNNNATTATGGAACTGAGTCAGCGATTCGT AGCGGCTGGAAAAGTAAAGC
    7. GCTTGAAGTGCTTCCTTGAAGTAAAAGGCA CGTTGG­AGAGGTTTNNNNATTATGGAACTGAGTCAGCGATTCGT AGCGGCTGGAAAAGTAAAGC
    8. GCTTGAAGTGCTTCCTTGAAGTAAAAGGCA CGTTGGAGAGGTTTNNNNATTATGGAACTGAGTCAGCGATTCGT AGCGGCTGGAAAAGTAAAGC
    9. GCTTGAAGTGCTTCCTTGAAGTAAAAGGCA CGTTGGAGAGGTTTNNNNATTATGGAACTGAGTCAGCGATTCGT AGCGGCTGGAAAAGTAAAGC
    10. GCTTGAAGTGCTTCCTTGAAGTAAAAGGCA CGTTGG­AGAGGTTTNNNNATTATGGAACTGAGTCAGCGATTCGT AGCGGCTGGAAAAGTAAAGC
    11. GCTTGAAGTGCTTCCTTGAAGTAAAAGGCA CGTTGGAGAGGTTTNNNNATTATGGAACTGAGTCAGCGATTCGT AGCGGCTGGAAAAGTAAAGC
    12. GCTTGAAGTGCTTCCTTGAAGTAAAAGGCAGCTCCAACGGTTCTAATTTGTGCTACGAACCTAAGTCAGTGCTCGAGCTCCGTCGTAGCCCAAGTCCTATAGTTGATAAGCAGATTATAACAACGAATCCTGATTTGTCTGCTCTTTGTGGTGGTGAAGATCCTCTTCAATTAGGAGATCATGTTCTGAGTAACTTTGAAGATTGGGATTCTTTGATGAGAGAACTTGGCTTGCACGACGATTCTGCTTCGCTTTCAAAAACGAATCCTCTCACCCACAG TCGCTGACTCAGTTCCATAATCTCTCCGAGTTTTCAGCTGAATCGAATCAGTTTCCTAGTCCTGATTTTTCTTTCTCAGACACTAATTTCCCTCAGCAGTTTCCGACGGTGAATCAGGCAAGTTTCATCAACGCCCTTGATCTCTCCGGGGATATCCACCAGAATTGGAGCGTAGGATTTGATTATGTGGATGAACTCATTCGTTTTGCTGAGTGTTTCGAAACAAACGCTTTCCAACTCGCACATGTGATACTGGCACGCCTCAATCAACGGCTCAGATCCGCAGCAGGAAAACCTCTCCAACGTAGCGGCTGGAAAAGTAAAGC
    13. GCTTGAAGTGCTTCCTTGAAGTAAAAGGCAGCTCCAACGGTTCTAATTTGTGCTACGAACCTAAGTCAGTGCTCGAGCTCCGTCGTAGCCCAAGTCCTATAGTTGATAAGCAGATTATAACAACGAATCCTGATTTGTCTGCTCTTTGTGGTGGTGAAGATCCTCTTCAATTAGGAGATCATGTTCTGAGTAACTTTGAAGATTGGGATTCTTTGATGAGAGAACTTGGCTTGCACGACGATTCTGCTTCGCTTTCAAAAACGAATCCTCTCACCCACAG TCGCTGACTCAGTTCCATAATCTCTCCGAGTTTTCAGCTGAATCGAATCAGTTTCCTAGTCCTGATTTTTCTTTCTCAGACACTAATTTCCCTCAGCAGTTTCCGACGGTGAATCAGGCAAGTTTCATCAACGCCCTTGATCTCTCCGGGGATATCCACCAGAATTGGAGCGTAGGATTTGATTATGTGGATGAACTCATTCGTTTTGCTGAGTGTTTCGAAACAAACGCTTTCCAACTCGCACATGTGATACTGGCACGCCTCAATCAACGGCTCAGATCCGCAGCAGGAAAACCTCTCCAACGTAGCGGCTGGAAAAGTAAAGC
    14. GCTTGAAGTGCTTCCTTGAAGTAAAAGGCAGCTCCAACGGTTCTAATTTGTGCTACGAACCTAAGTCAGTGCTCGAGCTCCGTCGTAGCCCAAGTCCTATAGTTGATAAGCAGATTATAACAACGAATCCTGATTTGTCTGCTCTTTGTGGTGGTGAAGATCCTCTTCAATTAGGAGATCATGTTCTGAGTAACTTTGAAGATTGGGATTCTTTGATGAGAGAACTTGGCTTGCACGACGATTCTGCTTCGCTTTCAAAAACGAATCCTCTCACCCACAG TCGCTGACTCAGTTCCATAATCTCTCCGAGTTTTCAGCTGAATCGAATCAGTTTCCTAGTCCTGATTTTTCTTTCTCAGACACTAATTTCCCTCAGCAGTTTCCGACGGTGAATCAGGCAAGTTTCATCAACGCCCTTGATCTCTCCGGGGATATCCACCAGAATTGGAGCGTAGGATTTGATTATGTGGATGAACTCATTCGTTTTGCTGAGTGTTTCGAAACAAACGCTTTCCAACTCGCACATGTGATACTGGCACGCCTCAATCAACGGCTCAGATCCGCAGCAGGAAAACCTCTCCAACGTAGCGGCTGGAAAAGTAAAGC
    15. GCTTGAAGTGCTTCCTTGAAGTAAAAGGCAGCTCCAACGGTTCTAATTTGTGCTACGAACCTAAGTCAGTGCTCGAGCTCCGTCGTAGCCCAAGTCCTATAGTTGATAAGCAGATTATAACAACGAATCCTGATTTGTCTGCTCTTTGTGGTGGTGAAGATCCTCTTCAATTAGGAGATCATGTTCTGAGTAACTTTGAAGATTGGGATTCTTTGATGAGAGAACTTGGCTTGCACGACGATTCTGCTTCGCTTTCAAAAACGAATCCTCTCACCCACAG TCGCTGACTCAGTTCCATAATCTCTCCGAGTTTTCAGCTGAATCGAATCAGTTTCCTAGTCCTGATTTTTCTTTCTCAGACACTAATTTCCCTCAGCAGTTTCCGACGGTGAATCAGGCAAGTTTCATCAACGCCCTTGATCTCTCCGGGGATATCCACCAGAATTGGAGCGTAGGATTTGATTATGTGGATGAACTCATTCGTTTTGCTGAGTGTTTCGAAACAAACGCTTTCCAACTCGCACATGTGATACTGGCACGCCTCAATCAACGGCTCAGATCCGCAGCAGGAAAACCTCTCCAACGTAGCGGCTGGAAAAGTAAAGC
    16. GCTTGAAGTGCTTCCTTGAAGTAAAAGGCAGCTCCAACGGTTCTAATTTGTGCTACGAACCTAAGTCAGTGCTCGAGCTCCGTCGTAGCCCAAGTCCTATAGTTGATAAGCAGATTATAACAACGAATCCTGATTTGTCTGCTCTTTGTGGTGGTGAAGATCCTCTTCAATTAGGAGATCATGTTCTGAGTAACTTTGAAGATTGGGATTCTTTGATGAGAGAACTTGGCTTGCACGACGATTCTGCTTCGCTTTCAAAAACGAATCCTCTCACCCACAG TCGCTGACTCAGTTCCATAATCTCTCCGAGTTTTCAGCTGAATCGAATCAGTTTCCTAGTCCTGATTTTTCTTTCTCAGACACTAATTTCCCTCAGCAGTTTCCGACGGTGAATCAGGCAAGTTTCATCAACGCCCTTGATCTCTCCGGGGATATCCACCAGAATTGGAGCGTAGGATTTGATTATGTGGATGAACTCATTCGTTTTGCTGAGTGTTTCGAAACAAACGCTTTCCAACTCGCACATGTGATACTGGCACGCCTCAATCAACGGCTCAGATCCGCAGCAGGAAAACCTCTCCAACGTAGCGGCTGGAAAAGTAAAGC

U3b-*SlGRAS8*-site1 U3d-*SlGRAS8*-site2 U6-29-*SlGRAS8*-site3

**WT** GCTTGAAGTGCTTCCTTGAAGTAAAAGGCAGCTCGTTGGAGAGGTTTNNNNATTATGGAACTGAGTCAGCGATTCGCTGTGGGTGAGAGGNNNNTCGTAGCACAAATTAGAACCGTTGGTAGCGGCTGGAAAAGTAAAGC

**L1-4** (chimeric)

1. GCTTGAAGTGCTTCCTTGAAGTAAAAGGCA TCGTTGGAGAGGTTTNNNNATTATGGAACTGAGTCAGCGATTCG**T**CTGTGGGTGAGAGGNNNNTCGTAGCACAAATTAGAACCGTTGGTAGCGGCTGGAAAAGTAAAGC
2. GCTTGAAGTGCTTCCTTGAAGTAAAAGGCA TCGTTGGAGAGGTTTNNNNATTATGGAACTGAGTCAGCGATTCG**T**CTGTGGGTGAGAGGNNNNTCGTAGCACAAATTAGAACCGTTGGTAGCGGCTGGAAAAGTAAAGC
3. GCTTGAAGTGCTTCCTTGAAGTAAAAGGCA TCGTTGGAGAGGTTTNNNNATTATGGAACTGAGTCAGCGATTCC**T**GTGGGTGAGAGGNNNNTCGTAGCACAAATTAGAACCGTTGGTAGCGGCTGGAAAAGTAAAGC
4. GCTTGAAGTGCTTCCTTGAAGTAAAAGGCA TCGTTGGAGAGGTTTNNNNATTATGGAACTGAGTCAGCGATTCG**T**CTGTGGGTGAGAGGNNNNTCGTAGCACAAATTAGAACCGTTGGTAGCGGCTGGAAAAGTAAAGC
5. GCTTGAAGTGCTTCCTTGAAGTAAAAGGCA TCGTTGGAGAGGTTTNNNNATTATGGAACTGAGTCAGCGATTCG**T**CTGTGGGTGAGAGGNNNNTCGTAGCACAAATTAGAACCGTTGGTAGCGGCTGGAAAAGTAAAGC
6. GCTTGAAGTGCTTCCTTGAAGTAAAAGGCA TCGTTGGAGAGGTTTNNNNATTATGGAACTGAGTCAGCGATTCC**T**GTGGGTGAGAGGNNNNTCGTAGCACAAATTAGAACCGTTGGTAGCGGCTGGAAAAGTAAAGCG
7. GCTTGAAGTGCTTCCTTGAAGTAAAAGGCA TCGTTGGAGAGGTTTNNNNATTATGGAACTGAGTCAGCGATTCG**T**CTGTGGGTGAGAGGNNNNTCGTAGCACAAATTAGAACCGTTGGTAGCGGCTGGAAAAGTAAAGC
8. GCTTGAAGTGCTTCCTTGAAGTAAAAGGCA TCGTTGGAGAGGTTTNNNNATTATGGAACTGAGTCAGCGATTCG**T**CTGTGGGTGAGAGGNNNNTCGTAGCACAAATTAGAACCGTTGGTAGCGGCTGGAAAAGTAAAGC
9. GCTTGAAGTGCTTCCTTGAAGTAAAAGGCA TCGTTGGAGAGGTTTNNNNATTATGGAACTGAGTCAGCGATTCG**T**CTGTGGGTGAGAGGNNNNTCGTAGCACAAATTAGAACCGTTGGTAGCGGCTGGAAAAGTAAAGC
10. GCTTGAAGTGCTTCCTTGAAGTAAAAGGCAGCTTCGTTGGAGAGGTTTNNNNATTATGGAACTGAGTCAGCGATTC CTGTGGGTGAGAGGNNNNTCGTAGCACAAATTAGAACCGTTGGTAGCGGCTGGAAAAGTAAAGC
11. GCTTGAAGTGCTTCCTTGAAGTAAAAGGCAGCTTCGTTGGAGAGGTTTNNNNATTATGGAACTGAGTCAGCGATTCG**T**CTGTGGGTGAGAGGNNNNTCGTAGCACAAATTAGAACCGTTGGTAGCGGCTGGAAAAGTAAAGC
12. GCTTGAAGTGCTTCCTTGAAGTAAAAGGCAGCTTCGTTGGAGAGGTTTNNNNATTATGGAACTGAGTCAGCGATTC CTGTGGGTGAGAGGNNNNTCGTAGCACAAATTAGAACCGTTGGTAGCGGCTGGAAAAGTAAAGC
13. GCTTGAAGTGCTTCCTTGAAGTAAAAGGCAGCTTCGTTGGAGAGGTTTNNNNATTATGGAACTGAGTCAGCGATTCG**T**CTGTGGGTGAGAGGNNNNTCGTAGCACAAATTAGAACCGTTGGTAGCGGCTGGAAAAGTAAAGC
14. GCTTGAAGTGCTTCCTTGAAGTAAAAGGCAGCTTCGTTGGAGAGGTTTNNNNATTATGGAACTGAGTCAGCGATTCG**T**CTGTGGGTGAGAGGNNNNTCGTAGCACAAATTAGAACCGTTGGTAGCGGCTGGAAAAGTAAAGC
15. GCTTGAAGTGCTTCCTTGAAGTAAAAGGCAGCTTCGTTGGAGAGGTTTNNNNATTATGGAACTGAGTCAGCGATTC CTGTGGGTGAGAGGNNNNTCGTAGCACAAATTAGAACCGTTGGTAGCGGCTGGAAAAGTAAAGC

U3b-*SlGRAS8*-site1 U3d-*SlGRAS8*-site2 U6-29-*SlGRAS8*-site3

**WT** GCTTGAAGTGCTTCCTTGAAGTAAAAGGCAGCTCGTTGGAGAGGTTTNNNNATTATGGAACTGAGTCAGCGATTCGCTGTGGGTGAGAGGNNNNTCGTAGCACAAATTAGAACCGTTGGTAGCGGCTGGAAAAGTAAAGC

**L2-1** (homozygous)

1. GCTTGAAGTGCTT TAGCGGCTGGAAAAGTAAAGC
2. GCTTGAAGTGCTT TAGCGGCTGGAAAAGTAAAGC
3. GCTTGAAGTGCTT TAGCGGCTGGAAAAGTAAAGC
4. GCTTGAAGTGCTT TAGCGGCTGGAAAAGTAAAGC
5. GCTTGAAGTGCTT TAGCGGCTGGAAAAGTAAAGC
6. GCTTGAAGTGCTT TAGCGGCTGGAAAAGTAAAGC
7. GCTTGAAGTGCTT TAGCGGCTGGAAAAGTAAAGC
8. GCTTGAAGTGCTT TAGCGGCTGGAAAAGTAAAGC
9. GCTTGAAGTGCTT TAGCGGCTGGAAAAGTAAAGC
10. GCTTGAAGTGCTT TAGCGGCTGGAAAAGTAAAGC
11. GCTTGAAGTGCTT TAGCGGCTGGAAAAGTAAAGC
12. GCTTGAAGTGCTT TAGCGGCTGGAAAAGTAAAGC
13. GCTTGAAGTGCTT TAGCGGCTGGAAAAGTAAAGC
14. GCTTGAAGTGCTT TAGCGGCTGGAAAAGTAAAGC
15. GCTTGAAGTGCTT TAGCGGCTGGAAAAGTAAAGC
16. GCTTGAAGTGCTT TAGCGGCTGGAAAAGTAAAGC
17. GCTTGAAGTGCTT TAGCGGCTGGAAAAGTAAAGC
18. GCTTGAAGTGCTT TAGCGGCTGGAAAAGTAAAGC
19. GCTTGAAGTGCTT TAGCGGCTGGAAAAGTAAAGC

U3b-*SlGRAS8*-site1 U3d-*SlGRAS8*-site2 U6-29-*SlGRAS8*-site3

**WT** GCTTGAAGTGCTTCCTTGAAGTAAAAGGCAGCTCGTTGGAGAGGTTTNNNNATTATGGAACTGAGTCAGCGATTCGCTGTGGGTGAGAGGNNNNTCGTAGCACAAATTAGAACCGTTGGTAGCGGCTGGAAAAGTAAAGC

**L2-2** (heterozygous)

- - 1. GCTTGAAGTGCTTCCTTGAAGTAAAACG TTGGAGAGGTTTNNNNATTATGGAACTGAGTCAGCGATTCG**A**CTGTGGGTGAGAGGNNNNTCGTAGCACAAATTAGAACC TAGCGGCTGGAAAAGTAAAGC
    2. GCTTGAAGTGCTTCCTTGAAGTAAAACG TTGGAGAGGTTTNNNNATTATGGAACTGAGTCAGCGATTCG**A**CTGTGGGTGAGAGGNNNNTCGTAGCACAAATTAGAACC TAGCGGCTGGAAAAGTAAAGC
    3. GCTTGAAGTGCTTCCTTGAAGTAAAACG TTGGAGAGGTTTNNNNATTATGGAACTGAGTCAGCGATTCG**A**CTGTGGGTGAGAGGNNNNTCGTAGCACAAATTAGAACC TAGCGGCTGGAAAAGTAAAGC
    4. GCTTGAAGTGCTTCCTTGAAGTAAAACG TTGGAGAGGTTTNNNNATTATGGAACTGAGTCAGCGATTCG**A**CTGTGGGTGAGAGGNNNNTCGTAGCACAAATTAGAACC TAGCGGCTGGAAAAGTAAAGC
    5. GCTTGAAGTGCTTCCTTGAAGTAAAAGGCAGCTTCGTTGGAGAGGTTTNNNNATTATGGAACTGAGTCAGCGATTCG**A**CTGTGGGTGAGAGGNNNNTCGTAGCACAAATTAGAACC TAGCGGCTGGAAAAGTAAAGC
    6. GCTTGAAGTGCTTCCTTGAAGTAAAAGGCAGCTTCGTTGGAGAGGTTTNNNNATTATGGAACTGAGTCAGCGATTCG**A**CTGTGGGTGAGAGGNNNNTCGTAGCACAAATTAGAACC TAGCGGCTGGAAAAGTAAAGC
    7. GCTTGAAGTGCTTCCTTGAAGTAAAAGGCAGCTTCGTTGGAGAGGTTTNNNNATTATGGAACTGAGTCAGCGATTCG**A**CTGTGGGTGAGAGGNNNNTCGTAGCACAAATTAGAACC TAGCGGCTGGAAAAGTAAAGC
    8. GCTTGAAGTGCTTCCTTGAAGTAAAAGGCAGCTTCGTTGGAGAGGTTTNNNNATTATGGAACTGAGTCAGCGATT TAGCGGCTGGAAAAGTAAAGC
    9. GCTTGAAGTGCTTCCTTGAAGTAAAAGGCAGCTTCGTTGGAGAGGTTTNNNNATTATGGAACTGAGTCAGCGATT TAGCGGCTGGAAAAGTAAAGC
    10. GCTTGAAGTGCTTCCTTGAAGTAAAAGGCAGCTTCGTTGGAGAGGTTTNNNNATTATGGAACTGAGTCAGCGATT TAGCGGCTGGAAAAGTAAAGC
    11. GCTTGAAGTGCTTCCTTGAAGTAAAAGGCAGCTTCGTTGGAGAGGTTTNNNNATTATGGAACTGAGTCAGCGATT TAGCGGCTGGAAAAGTAAAGC
    12. GCTTGAAGTGCTTCCTTGAAGTAAAAGGCAGCTTCGTTGGAGAGGTTTNNNNATTATGGAACTGAGTCAGCGATT TAGCGGCTGGAAAAGTAAAGC
    13. GCTTGAAGTGCTTCCTTGAAGTAAAAGGCAGCTTCGTTGGAGAGGTTTNNNNATTATGGAACTGAGTCAGCGATT TAGCGGCTGGAAAAGTAAAGC
    14. GCTTGAAGTGCTTCCTTGAAGTAAAAGGCAGCTTCGTTGGAGAGGTTTNNNNATTATGGAACTGAGTCAGCGATT TAGCGGCTGGAAAAGTAAAGC

U3b-*SlGRAS8*-site1 U3d-*SlGRAS8*-site2 U6-29-*SlGRAS8*-site3

**WT** GCTTGAAGTGCTTCCTTGAAGTAAAAGGCAGCTCGTTGGAGAGGTTTNNNNATTATGGAACTGAGTCAGCGATTCGCTGTGGGTGAGAGGNNNNTCGTAGCACAAATTAGAACCGTTGGTAGCGGCTGGAAAAGTAAAGC

**L2-3**  (heterozygous)

- - 1. GCTTGAAGTGCTTCCTTGAAGTAAAAGGCAGCT GTTGGAGAGGTTTNNNNATTATGGAACTGAGTCAGCGATTCG**A**CTGTGGGTGAGAGGNNNNTCGTAGCACAAATTAGAACCGTTGGTAGCGGCTGGAAAAGTAAAGC
    2. GCTTGAAGTGCTTCCTTGAAGTAAAAGGCAGCT GTTGGAGAGGTTTNNNNATTATGGAACTGAGTCAGCGATTCG**A**CTGTGGGTGAGAGGNNNNTCGTAGCACAAATTAGAACCGTTGGTAGCGGCTGGAAAAGTAAAGC
    3. GCTTGAAGTGCTTCCTTGAAGTAAAAGGCAGCT GTTGGAGAGGTTTNNNNATTATGGAACTGAGTCAGCGATTCG**A**CTGTGGGTGAGAGGNNNNTCGTAGCACAAATTAGAACCGTTGGTAGCGGCTGGAAAAGTAAAGC
    4. GCTTGAAGTGCTTCCTTGAAGTAAAAGGCAGCT GTTGGAGAGGTTTNNNNATTATGGAACTGAGTCAGCGATTCG**A**CTGTGGGTGAGAGGNNNNTCGTAGCACAAATTAGAACCGTTGGTAGCGGCTGGAAAAGTAAAGC
    5. GCTTGAAGTGCTTCCTTGAAGTAAAAGGCAGCT GTTGGAGAGGTTTNNNNATTATGGAACTGAGTCAGCGATTCG**A**CTGTGGGTGAGAGGNNNNTCGTAGCACAAATTAGAACCGTTGGTAGCGGCTGGAAAAGTAAAGC
    6. GCTTGAAGTGCTTCCTTGAAGTAAAAGGCAGCT GTTGGAGAGGTTTNNNNATTATGGAACTGAGTCAGCGATTCG**A**CTGTGGGTGAGAGGNNNNTCGTAGCACAAATTAGAACCGTTGGTAGCGGCTGGAAAAGTAAAGC
    7. GCTTGAAGTGCTTCCTTGAAGTAAAAGGCAGCT GTTGGAGAGGTTTNNNNATTATGGAACTGAGTCAGCGATTCG**A**CTGTGGGTGAGAGGNNNNTCGTAGCACAAATTAGAACCGTTGGTAGCGGCTGGAAAAGTAAAGC
    8. GCTTGAAGTGCTTCCTTGAAGTAAAAGGCAGCT GTTGGAGAGGTTTNNNNATTATGGAACTGAGTCAGCGATTCG**A**CTGTGGGTGAGAGGNNNNTCGTAGCACAAATTAGAACCGTTG TAGCGGCTGGAAAAGTAAAGC
    9. GCTTGAAGTGCTTCCTTGAAGTAAAAGGCAGCT GTTGGAGAGGTTTNNNNATTATGGAACTGAGTCAGCGATTCG**A**CTGTGGGTGAGAGGNNNNTCGTAGCACAAATTAGAACCGTTGGTAGCGGCTGGAAAAGTAAAGC
    10. GCTTGAAGTGCTTCCTTGAAGTAAAAGGCAGCTGAATCGCTGACTCAGTTCCATAATCTCTCCGAGTTTTCAGCTGAATCGAATCAGTTTCCTAGTCCTGATTTTTCTTTCTCAGACACTAATTTCCCTCAGCAGTTTCCGACGGTGAATCAGGCAAGTTTCATCAACGCCCTTGATCTCTCCGGGGATATCCACCAGAATTGGAGCGTAGGATTTGATTATGTGGATGAACTCATTCGTTTTGCTGAGTGTTTCGAAACAAACGCTTTCCAACTCGCACATGTGATACTGGCACGCCTCAATCAACGGCTCAGATCCGCAGCAGGAAAACCTCTCCAACGCTGTGGGTGAGAGGNNNNTCGTAGCACAAATTAGAACCGTTG TAGCGGCTGGAAAAGTAAAGC
    11. GCTTGAAGTGCTTCCTTGAAGTAAAAGGCAGCTG.AATCGCTGACTCAGTTCCATAATCTCTCCGAGTTTTCAGCTGAATCGAATCAGTTTCCTAGTCCTGATTTTTCTTTCTCAGACACTAATTTCCCTCAGCAGTTTCCGACGGTGAATCAGGCAAGTTTCATCAACGCCCTTGATCTCTCCGGGGATATCCACCAGAATTGGAGCGTAGGATTTGATTATGTGGATGAACTCATTCGTTTTGCTGAGTGTTTCGAAACAAACGCTTTCCAACTCGCACATGTGATACTGGCACGCCTCAATCAACGGCTCAGATCCGCAGCAGGAAAACCTCTCCAACG.CTGTGGGTGAGAGGNNNNTCGTAGCACAAATTAGAACCGTTG TAGCGGCTGGAAAAGTAAAGC
    12. GCTTGAAGTGCTTCCTTGAAGTAAAAGGCAGCTGAATCGCTGACTCAGTTCCATAATCTCTCCGAGTTTTCAGCTGAATCGAATCAGTTTCCTAGTCCTGATTTTTCTTTCTCAGACACTAATTTCCCTCAGCAGTTTCCGACGGTGAATCAGGCAAGTTTCATCAACGCCCTTGATCTCTCCGGGGATATCCACCAGAATTGGAGCGTAGGATTTGATTATGTGGATGAACTCATTCGTTTTGCTGAGTGTTTCGAAACAAACGCTTTCCAACTCGCACATGTGATACTGGCACGCCTCAATCAACGGCTCAGATCCGCAGCAGGAAAACCTCTCCAACGCTGTGGGTGAGAGGNNNNTCGTAGCACAAATTAGAACCGTTG TAGCGGCTGGAAAAGTAAAGC
    13. GCTTGAAGTGCTTCCTTGAAGTAAAAGGCAGCTGAATCGCTGACTCAGTTCCATAATCTCTCCGAGTTTTCAGCTGAATCGAATCAGTTTCCTAGTCCTGATTTTTCTTTCTCAGACACTAATTTCCCTCAGCAGTTTCCGACGGTGAATCAGGCAAGTTTCATCAACGCCCTTGATCTCTCCGGGGATATCCACCAGAATTGGAGCGTAGGATTTGATTATGTGGATGAACTCATTCGTTTTGCTGAGTGTTTCGAAACAAACGCTTTCCAACTCGCACATGTGATACTGGCACGCCTCAATCAACGGCTCAGATCCGCAGCAGGAAAACCTCTCCAACGCTGTGGGTGAGAGGNNNNTCGTAGCACAAATTAGAACCGTTG TAGCGGCTGGAAAAGTAAAGC
    14. GCTTGAAGTGCTTCCTTGAAGTAAAAGGCAGCTGAATCGCTGACTCAGTTCCATAATCTCTCCGAGTTTTCAGCTGAATCGAATCAGTTTCCTAGTCCTGATTTTTCTTTCTCAGACACTAATTTCCCTCAGCAGTTTCCGACGGTGAATCAGGCAAGTTTCATCAACGCCCTTGATCTCTCCGGGGATATCCACCAGAATTGGAGCGTAGGATTTGATTATGTGGATGAACTCATTCGTTTTGCTGAGTGTTTCGAAACAAACGCTTTCCAACTCGCACATGTGATACTGGCACGCCTCAATCAACGGCTCAGATCCGCAGCAGGAAAACCTCTCCAACGCTGTGGGTGAGAGGNNNNTCGTAGCACAAATTAGAACCGTTG TAGCGGCTGGAAAAGTAAAGC
    15. GCTTGAAGTGCTTCCTTGAAGTAAAAGGCAGCTGAATCGCTGACTCAGTTCCATAATCTCTCCGAGTTTTCAGCTGAATCGAATCAGTTTCCTAGTCCTGATTTTTCTTTCTCAGACACTAATTTCCCTCAGCAGTTTCCGACGGTGAATCAGGCAAGTTTCATCAACGCCCTTGATCTCTCCGGGGATATCCACCAGAATTGGAGCGTAGGATTTGATTATGTGGATGAACTCATTCGTTTTGCTGAGTGTTTCGAAACAAACGCTTTCCAACTCGCACATGTGATACTGGCACGCCTCAATCAACGGCTCAGATCCGCAGCAGGAAAACCTCTCCAACGCTGTGGGTGAGAGGNNNNTCGTAGCACAAATTAGAACCGTTG TAGCGGCTGGAAAAGTAAAGC

U3b-*SlGRAS8*-site1 U3d-*SlGRAS8*-site2 U6-29-*SlGRAS8*-site3

**WT** GCTTGAAGTGCTTCCTTGAAGTAAAAGGCAGCTCGTTGGAGAGGTTTNNNNATTATGGAACTGAGTCAGCGATTCGCTGTGGGTGAGAGGNNNNTCGTAGCACAAATTAGAACCGTTGGTAGCGGCTGGAAAAGTAAAGC

**L2-4**  (chimeric)

1. GCTTGAAGTGCTTCCTTGAAGTAAAAGGCAGC CGTTGGAGAGGTTTNNNNATTATGGAACTGAGTCAGCGATTC CTGTGGGTGAGAGGNNNNTCGTAGCACAAATTAGAACCGTTGGTAGCGGCTGGAAAAGTAAAGC
2. GCTTGAAGTGCTTCCTTGAAGTAAAAGGCAGC CGTTGGAGAGGTTTNNNNATTATGGAACTGAGTCAGCGATTC CTGTGGGTGAGAGGNNNNTCGTAGCACAAATTAGAACCGTTGGTAGCGGCTGGAAAAGTAAAGC
3. GCTTGAAGTGCTTCCTTGAAGTAAAAGGCAGC CGTTGGAGAGGTTTNNNNATTATGGAACTGAGTCAGCGATTC CTGTGGGTGAGAGGNNNNTCGTAGCACAAATTAGAACCGTTGGTAGCGGCTGGAAAAGTAAAGC
4. GCTTGAAGTGCTTCCTTGAAGTAAAAGGCAGC CGTTGGAGAGGTTTNNNNATTATGGAACTGAGTCAGCGATTC CTGTGGGTGAGAGGNNNNTCGTAGCACAAATTAGAACCGTTGGTAGCGGCTGGAAAAGTAAAGC
5. GCTTGAAGTGCTTCCTTGAAGTAAAAGGCAGC CGTTGGAGAGGTTTNNNNATTATGGAACTGAGTCAGCGATTC CTGTGGGTGAGAGGNNNNTCGTAGCACAAATTAGAACCGTTGG**A**TAGCGGCTGGAAAAGTAAAGC
6. GCTTGAAGTGCTTCCTTGAAGTAAAAGGCAGC CGTTGGAGAGGTTTNNNNATTATGGAACTGAGTCAGCGATTC CTGTGGGTGAGAGGNNNNTCGTAGCACAAATTAGAACCGTTGGTAGCGGCTGGAAAAGTAAAGC
7. GCTTGAAGTGCTTCCTTGAAGTAAAAGGC TCGTTGGAGAGGTTTNNNNATTATGGAACTGAGTCAGCGATTC CTGTGGGTGAGAGGNNNNTCGTAGCACAAATTAGAACCGTTGGTAGCGGCTGGAAAAGTAAAGC
8. GCTTGAAGTGCTTCCTTGAAGTAAAAGGC TCGTTGGAGAGGTTTNNNNATTATGGAACTGAGTCAGCGA GAGGNNNNTCGTAGCACAAATTAGAACCGTTGG**A**TAGCGGCTGGAAAAGTAAAGC
9. GCTTGAAGTGCTTCCTTGAAGTAAAAGGCAGCCGTTGGAGAGGTTTNNNNATTATGGAACTGAGTCAGCGA GAGGNNNNTCGTAGCACAAATTAGAACCGTTGG**A**TAGCGGCTGGAAAAGTAAAGC
10. GCTTGAAGTGCTTCCTTGAAGTAAAAGGCTCGTTGGAGAGGTTTNNNNATTATGGAACTGAGTCAGCGA GAGGNNNNTCGTAGCACAAATTAGAACCGTTGG**A**TAGCGGCTGGAAAAGTAAAGC
11. GCTTGAAGTGCTTCCTTGAAGTAAAAGGCAGCCGTTGGAGAGGTTTNNNNTTATGGAACTGAGTCAGCGA GAGGNNNNTCGTAGCACAAATTAGAACCGTTGG**A**TAGCGGCTGGAAAAGTAAAGC
12. GCTTGAAGTGCTTCCTTGAAGTAAAAGGCTCGTTGGAGAGGTTTNNNNATTATGGAACTGAGTCAGCGA GAGGNNNNTCGTAGCACAAATTAGAACCGTTGG**A**TAGCGGCTGGAAAAGTAAAGC
13. GCTTGAAGTGCTTCCTTGAAGTAAAAGGCAGCCGTTGGAGAGGTTTNNNNATTATGGAACTGAGTCAGCGA GAGGNNNNTCGTAGCACAAATTAGAACCGTTGG**A**TAGCGGCTGGAAAAGTAAAGC
14. GCTTGAAGTGCTTCCTTGAAGTAAAAGGCTCGTTGGAGAGGTTTNNNNATTATGGAACTGAGTCAGCGA GAGGNNNNTCGTAGCACAAATTAGAACCGTTGGTAGCGGCTGGAAAAGTAAAGC

U3b-*SlGRAS8*-site1 U3d-*SlGRAS8*-site2 U6-29-*SlGRAS8*-site3

**WT** GCTTGAAGTGCTTCCTTGAAGTAAAAGGCAGCTCGTTGGAGAGGTTTNNNNATTATGGAACTGAGTCAGCGATTCGCTGTGGGTGAGAGGNNNNTCGTAGCACAAATTAGAACCGTTGGTAGCGGCTGGAAAAGTAAAGC

**L3-1** (heterozygous)

1. GCTTGAAGTGCTTCCTTGAAGTAAAAGGCAG TCGTTGGAGAGGTTTNNNNATTATGGAACTGAGTCAGCGATTC CTGTGGGTGAGAGGNNNNTCGTAGCACAAATTAGAACCGTTGGTAGCGGCTGGAAAAGTAAAGC
2. GCTTGAAGTGCTTCCTTGAAGTAAAAGGCAG TCGTTGGAGAGGTTTNNNNATTATGGAACTGAGTCAGCGATTCG**A**CTGTGGGTGAGAGGNNNNTCGTAGCACAAATTAGAACCGTTGGTAGCGGCTGGAAAAGTAAAGC
3. GCTTGAAGTGCTTCCTTGAAGTAAAAGGCAG TCGTTGGAGAGGTTTNNNNATTATGGAACTGAGTCAGCGATTC CTGTGGGTGAGAGGNNNNTCGTAGCACAAATTAGAACCGTTGGTAGCGGCTGGAAAAGTAAAGC
4. GCTTGAAGTGCTTCCTTGAAGTAAAAGGCAG TCGTTGGAGAGGTTTNNNNATTATGGAACTGAGTCAGCGATTCG**A**CTGTGGGTGAGAGGNNNNTCGTAGCACAAATTAGAACCGTTGGTAGCGGCTGGAAAAGTAAAGC
5. GCTTGAAGTGCTTCCTTGAAGTAAAAGGCAG TCGTTGGAGAGGTTTNNNNATTATGGAACTGAGTCAGCGATTCG**A**CTGTGGGTGAGAGGNNNNTCGTAGCACAAATTAGAACCGTTGGTAGCGGCTGGAAAAGTAAAGC
6. GCTTGAAGTGCTTCCTTGAAGTAAAAGGCAG TCGTTGGAGAGGTTTNNNNATTATGGAACTGAGTCAGCGATTCG**A**CTGTGGGTGAGAGGNNNNTCGTAGCACAAATTAGAACCGTTGGTAGCGGCTGGAAAAGTAAAGC
7. GCTTGAAGTGCTTCCTTGAAGTAAAAGGCAG TCGTTGGAGAGGTTTNNNNATTATGGAACTGAGTCAGCGATTCG**A**CTGTGGGTGAGAGGNNNNTCGTAGCACAAATTAGAACCGTTGGTAGCGGCTGGAAAAGTAAAGC
8. GCTTGAAGTGCTTCCTTGAAGTAAAAGGCAG TCGTTGGAGAGGTTTNNNNATTATGGAACTGAGTCAGCGATTCG**A**CTGTGGGTGAGAGGNNNNTCGTAGCACAAATTAGAACCGTTGGTAGCGGCTGGAAAAGTAAAGC
9. GCTTGAAGTGCTTCCTTGAAGTAAAAGGCAG TCGTTGGAGAGGTTTNNNNATTATGGAACTGAGTCAGCGATTC CTGTGGGTGAGAGGNNNNTCGTAGCACAAATTAGAACCGTTGGTAGCGGCTGGAAAAGTATAGC
10. GCTTGAAGTGCTTCCTTGAAGTAAAAGGCAG TCGTTGGAGAGGTTTNNNNATTATGGAACTGAGTCAGCGATTC CTGTGGGTGAGAGGNNNNTCGTAGCACAAATTAGAACCGTTGGTAGCGGCTGGAAAAGTAAAGC
11. GCTTGAAGTGCTTCCTTGAAGTAAAAGGCAG TCGTTGGAGAGGTTTNNNNATTATGGAACTGAGTCAGCGATTCG**A**CTGTGGGTGAGAGGNNNNTCGTAGCACAAATTAGAACCGTTGGTAGCGGCTGGAAAAGTAAAGC
12. GCTTGAAGTGCTTCCTTGAAGTAAAAGGCAGC TGTTGGAGAGGTTTNNNNATTATGGAACTGAGTCAGCGATTC CTGTGGGTGAGAGGNNNNTCGTAGCACAAATTAGAACCGTTGGTAGCGGCTGGAAAAGTAAAGC
13. GCTTGAAGTGCTTCCTTGAAGTAAAAGGCAGC TGTTGGAGAGGTTTNNNNATTATGGAACTGAGTCAGCGATTCG**A**CTGTGGGTGAGAGGNNNNTCGTAGCACAAATTAGAACCGTTGGTAGCGGCTGGAAAAGTAAAGC
14. GCTTGAAGTGCTTCCTTGAAGTAAAAGGCAGC TGTTGGAGAGGTTTNNNNATTATGGAACTGAGTCAGCGATTC CTGTGGGTGAGAGGNNNNTCGTAGCACAAATTAGAACCGTTGGTAGCGGCTGGAAAAGTAAAGC
15. GCTTGAAGTGCTTCCTTGAAGTAAAAGGCAGC TGTTGGAGAGGTTTNNNNATTATGGAACTGAGTCAGCGATTCG**A**CTGTGGGTGAGAGGNNNNTCGTAGCACAAATTAGAACCGTTGGTAGCGGCTGGAAAAGTAAAGC
16. GCTTGAAGTGCTTCCTTGAAGTAAAAGGCAGC TGTTGGAGAGGTTTNNNNATTATGGAACTGAGTCAGCGATTCG**A**CTGTGGGTGAGAGGNNNNTCGTAGCACAAATTAGAACCGTTGGTAGCGGCTGGAAAAGTAAAGC
17. GCTTGAAGTGCTTCCTTGAAGTAAAAGGCAGC TGTTGGAGAGGTTTNNNNATTATGGAACTGAGTCAGCGATTCG**A**CTGTGGGTGAGAGGNNNNTCGTAGCACAAATTAGAACCGTTGGTAGCGGCTGGAAAAGTAAAGC
18. GCTTGAAGTGCTTCCTTGAAGTAAAAGGCAGC TGTTGGAGAGGTTTNNNNATTATGGAACTGAGTCAGCGATTC CTGTGGGTGAGAGGNNNNTCGTAGCACAAATTAGAACCGTTGGTAGCGGCTGGAAAAGTAAAGC
19. GCTTGAAGTGCTTCCTTGAAGTAAAAGGCAGC TGTTGGAGAGGTTTNNNNATTATGGAACTGAGTCAGCGATTC CTGTGGGTGAGAGGNNNNTCGTAGCACAAATTAGAACCGTTGGTAGCGGCTGGAAAAGTAAAGC

U3b-*SlGRAS8*-site1 U3d-*SlGRAS8*-site2 U6-29-*SlGRAS8*-site3

**WT** GCTTGAAGTGCTTCCTTGAAGTAAAAGGCAGCTCGTTGGAGAGGTTTNNNNATTATGGAACTGAGTCAGCGATTCGCTGTGGGTGAGAGGNNNNTCGTAGCACAAATTAGAACCGTTGGTAGCGGCTGGAAAAGTAAAGC

**L3-2 (**chimeric**)**

1. GCTTGAAGTGCTTCCTTGAAGTAAAAGGCAGC CGTTGGAGAGGTTTNNNNATTATGGAACTGAGTCAGCGATTCG**A**CTGTGGGTGAGAGGNNNNTCGTAGCACAAATTAGAACCGTTGGTAGCGGCTGGAAAAGTAAAGC
2. GCTTGAAGTGCTTCCTTGAAGTAAAAGGCAGC CGTTGGAGAGGTTTNNNNATTATGGAACTGAGTCAGCGATTCG**A**CTGTGGGTGAGAGGNNNNTCGTAGCACAAATTAGAACCGTTGGTAGCGGCTGGAAAAGTAAAG
3. GCTTGAAGTGCTTCCTTGAAGTAAAAGGCA**T** CGTTGGAGAGGTTTNNNNATTATGGAACTGAGTCAGCGATTCGACTGTGGGTGAGAGGNNNNTCGTAGCACAAATTAGAACCGTTGGTAGCGGCTGGAAAAGTAAAGC
4. GCTTGAAGTGCTTCCTTGAAGTAAAAGGCAGC CGTTGGAGAGGTTTNNNNATTATGGAACTGAGTCAGCGATCTGTGGGTGAGAGGNNNNTCGTAGCACAAATTAGAACCGTTAGCGGCTGGAAAAGTAAAGC
5. GCTTGAAGTGCTTCCTTGAAGTAAAAGGCAGC CGTTGGAGAGGTTTNNNNATTATGGAACTGAGTCAGCGATCTGTGGGTGAGAGGNNNNTCGTAGCACAAATTAGAACCGTTAGCGGCTGGAAAAGTAAAGC
6. GCTTGAAGTGCTTCCTTGAAGTAAAAGGCA**T** CGTTGGAGAGGTTTNNNNATTATGGAACTGAGTCAGCGATCTGTGGGTGAGAGGNNNNTCGTAGCACAAATTAGAACCGTTAGCGGCTGGAAAAGTAAAGC
7. GCTTGAAGTGCTTCCTTGAAGTAAAAGGCAGC CGTTGGAGAGGTTTNNNNATTATGGAACTGAGTCAGCGATCTGTGGGTGAGAGGNNNNTCGTAGCACAAATTAGAACCGTTAGCGGCTGGAAAAGTAAAGC
8. GCTTGAAGTGCTTCCTTGAAGTAAAAGGCAGC CGTTGGAGAGGTTTNNNNATTATGGAACTGAGTCAGCGATTCGACTGTGGGTGAGAGGNNNNTCGTAGCACAAATTAGAACCGTTAGCGGCTGGAAAAGTAAAGC
9. GCTTGAAGTGCTTCCTTGAAGTAAAAGGCAGC CGTTGGAGAGGTTTNNNNATTATGGAACTGAGTCAGCGATCTGTGGGTGAGAGGNNNNTCGTAGCACAAATTAGAACCGTTAGCGGCTGGAAAAGTAAAGC
10. GCTTGAAGTGCTTCCTTGAAGTAAAAGGCAGC CGTTGGAGAGGTTTNNNNATTATGGAACTGAGTCAGCGATCTGTGGGTGAGAGGNNNNTCGTAGCACAAATTAGAACCGTTAGCGGCTGGAAAAGTAAAGC
11. GCTTGAAGTGCTTCCTTGAAGTAAAAGGCAGC CGTTGGAGAGGTTTNNNNATTATGGAACTGAGTCAGCGATTCGACTGTGGGTGAGAGGNNNNTCGTAGCACAAATTAGAACCGTTAGCGGCTGGAAAAGTAAAGC
12. GCTTGAAGTGCTTCCTTGAAGTAAAAGGCA**T** CGTTGGAGAGGTTTNNNNATTATGGAACTGAGTCAGCGATTCGACTGTGGGTGAGAGGNNNNTCGTAGCACAAATTAGAACCGTTAGCGGCTGGAAAAGTAAAGC
13. GCTTGAAGTGCTTCCTTGAAGTAAAAGGCA**T** CGTTGGAGAGGTTTNNNNATTATGGAACTGAGTCAGCGATCTGTGGGTGAGAGGNNNNTCGTAGCACAAATTAGAACCGTTAGCGGCTGGAAAAGTAAAGC
14. GCTTGAAGTGCTTCCTTGAAGTAAAAGGCA**T**  CGTTGGAGAGGTTTNNNNATTATGGAACTGAGTCAGCGATCTGTGGGTGAGAGGNNNNTCGTAGCACAAATTAGAACCGTTAGCGGCTGGAAAAGTAAAGC

U3b-*SlGRAS8*-site1 U3d-*SlGRAS8*-site2 U6-29-*SlGRAS8*-site3

**WT** GCTTGAAGTGCTTCCTTGAAGTAAAAGGCAGCTCGTTGGAGAGGTTTNNNNATTATGGAACTGAGTCAGCGATTCGCTGTGGGTGAGAGGNNNNTCGTAGCACAAATTAGAACCGTTGGTAGCGGCTGGAAAAGTAAAGC

**L3-3** (homozygous)

1. GCTTGAAGTGCTTCCTTGAAGTAAAAGGCAGCTCCAACGGTTCTAATTTGTGCTACGAACCTAAGTCAGTGCTCGAGCTCCGTCGTAGCCCAAGTCCTATAGTTGATAAGCAGATTATAACAACGAATCCTGATTTGTCTGCTCTTTGTGGTGGTGAAGATCCTCTTCAATTAGGAGATCATGTTCTGAGTAACTTTGAAGATTGGGATTCTTTGATGAGAGAACTTGGCTTGCACGACGATTCTGCTTCGCTTTCAAAAACGAATCCTCTCACCCACAGGAATCGCTGACTCAGTTCCATAATCTCTCCGAGTTTTCAGCTGAATCGAATCAGTTTCCTAGTCCTGATTTTTCTTTCTCAGACACTAATTTCCCTCAGCAGTTTCCGACGGTGAATCAGGCAAGTTTCATCAACGCCCTTGATCTCTCCGGGGATATCCACCAGAATTGGAGCGTAGGATTTGATTATGTGGATGAACTCATTCGTTTTGCTGAGTGTTTCGAAACAAACGCTTTCCAACTCGCACATGTGATACTGGCACGCCTCAATCAACGGCTCAGATCCGCAGCAGGAAAACCTCTCCAACGATAGCGGCTGGAAAAGTAAAGC
2. GCTTGAAGTGCTTCCTTGAAGTAAAAGGCAGCTCCAACGGTTCTAATTTGTGCTACGAACCTAAGTCAGTGCTCGAGCTCCGTCGTAGCCCAAGTCCTATAGTTGATAAGCAGATTATAACAACGAATCCTGATTTGTCTGCTCTTTGTGGTGGTGAAGATCCTCTTCAATTAGGAGATCATGTTCTGAGTAACTTTGAAGATTGGGATTCTTTGATGAGAGAACTTGGCTTGCACGACGATTCTGCTTCGCTTTCAAAAACGAATCCTCTCACCCACAGGAATCGCTGACTCAGTTCCATAATCTCTCCGAGTTTTCAGCTGAATCGAATCAGTTTCCTAGTCCTGATTTTTCTTTCTCAGACACTAATTTCCCTCAGCAGTTTCCGACGGTGAATCAGGCAAGTTTCATCAACGCCCTTGATCTCTCCGGGGATATCCACCAGAATTGGAGCGTAGGATTTGATTATGTGGATGAACTCATTCGTTTTGCTGAGTGTTTCGAAACAAACGCTTTCCAACTCGCACATGTGATACTGGCACGCCTCAATCAACGGCTCAGATCCGCAGCAGGAAAACCTCTCCAACGATAGCGGCTGGAAAAGTAAAGC
3. GCTTGAAGTGCTTCCTTGAAGTAAAAGGCAGCTCCAACGGTTCTAATTTGTGCTACGAACCTAAGTCAGTGCTCGAGCTCCGTCGTAGCCCAAGTCCTATAGTTGATAAGCAGATTATAACAACGAATCCTGATTTGTCTGCTCTTTGTGGTGGTGAAGATCCTCTTCAATTAGGAGATCATGTTCTGAGTAACTTTGAAGATTGGGATTCTTTGATGAGAGAACTTGGCTTGCACGACGATTCTGCTTCGCTTTCAAAAACGAATCCTCTCACCCACAGGAATCGCTGACTCAGTTCCATAATCTCTCCGAGTTTTCAGCTGAATCGAATCAGTTTCCTAGTCCTGATTTTTCTTTCTCAGACACTAATTTCCCTCAGCAGTTTCCGACGGTGAATCAGGCAAGTTTCATCAACGCCCTTGATCTCTCCGGGGATATCCACCAGAATTGGAGCGTAGGATTTGATTATGTGGATGAACTCATTCGTTTTGCTGAGTGTTTCGAAACAAACGCTTTCCAACTCGCACATGTGATACTGGCACGCCTCAATCAACGGCTCAGATCCGCAGCAGGAAAACCTCTCCAACGATAGCGGCTGGAAAAGTAAAGC
4. GCTTGAAGTGCTTCCTTGAAGTAAAAGGCAGCTCCAACGGTTCTAATTTGTGCTACGAACCTAAGTCAGTGCTCGAGCTCCGTCGTAGCCCAAGTCCTATAGTTGATAAGCAGATTATAACAACGAATCCTGATTTGTCTGCTCTTTGTGGTGGTGAAGATCCTCTTCAATTAGGAGATCATGTTCTGAGTAACTTTGAAGATTGGGATTCTTTGATGAGAGAACTTGGCTTGCACGACGATTCTGCTTCGCTTTCAAAAACGAATCCTCTCACCCACAGGAATCGCTGACTCAGTTCCATAATCTCTCCGAGTTTTCAGCTGAATCGAATCAGTTTCCTAGTCCTGATTTTTCTTTCTCAGACACTAATTTCCCTCAGCAGTTTCCGACGGTGAATCAGGCAAGTTTCATCAACGCCCTTGATCTCTCCGGGGATATCCACCAGAATTGGAGCGTAGGATTTGATTATGTGGATGAACTCATTCGTTTTGCTGAGTGTTTCGAAACAAACGCTTTCCAACTCGCACATGTGATACTGGCACGCCTCAATCAACGGCTCAGATCCGCAGCAGGAAAACCTCTCCAACGATAGCGGCTGGAAAAGTAAAGC
5. GCTTGAAGTGCTTCCTTGAAGTAAAAGGCAGCTCCAACGGTTCTAATTTGTGCTACGAACCTAAGTCAGTGCTCGAGCTCCGTCGTAGCCCAAGTCCTATAGTTGATAAGCAGATTATAACAACGAATCCTGATTTGTCTGCTCTTTGTGGTGGTGAAGATCCTCTTCAATTAGGAGATCATGTTCTGAGTAACTTTGAAGATTGGGATTCTTTGATGAGAGAACTTGGCTTGCACGACGATTCTGCTTCGCTTTCAAAAACGAATCCTCTCACCCACAGGAATCGCTGACTCAGTTCCATAATCTCTCCGAGTTTTCAGCTGAATCGAATCAGTTTCCTAGTCCTGATTTTTCTTTCTCAGACACTAATTTCCCTCAGCAGTTTCCGACGGTGAATCAGGCAAGTTTCATCAACGCCCTTGATCTCTCCGGGGATATCCACCAGAATTGGAGCGTAGGATTTGATTATGTGGATGAACTCATTCGTTTTGCTGAGTGTTTCGAAACAAACGCTTTCCAACTCGCACATGTGATACTGGCACGCCTCAATCAACGGCTCAGATCCGCAGCAGGAAAACCTCTCCAACGATAGCGGCTGGAAAAGTAAAGC
6. GCTTGAAGTGCTTCCTTGAAGTAAAAGGCAGCTCCAACGGTTCTAATTTGTGCTACGAACCTAAGTCAGTGCTCGAGCTCCGTCGTAGCCCAAGTCCTATAGTTGATAAGCAGATTATAACAACGAATCCTGATTTGTCTGCTCTTTGTGGTGGTGAAGATCCTCTTCAATTAGGAGATCATGTTCTGAGTAACTTTGAAGATTGGGATTCTTTGATGAGAGAACTTGGCTTGCACGACGATTCTGCTTCGCTTTCAAAAACGAATCCTCTCACCCACAGGAATCGCTGACTCAGTTCCATAATCTCTCCGAGTTTTCAGCTGAATCGAATCAGTTTCCTAGTCCTGATTTTTCTTTCTCAGACACTAATTTCCCTCAGCAGTTTCCGACGGTGAATCAGGCAAGTTTCATCAACGCCCTTGATCTCTCCGGGGATATCCACCAGAATTGGAGCGTAGGATTTGATTATGTGGATGAACTCATTCGTTTTGCTGAGTGTTTCGAAACAAACGCTTTCCAACTCGCACATGTGATACTGGCACGCCTCAATCAACGGCTCAGATCCGCAGCAGGAAAACCTCTCCAACGATAGCGGCTGGAAAAGTAAAGC
7. GCTTGAAGTGCTTCCTTGAAGTAAAAGGCAGCTCCAACGGTTCTAATTTGTGCTACGAACCTAAGTCAGTGCTCGAGCTCCGTCGTAGCCCAAGTCCTATAGTTGATAAGCAGATTATAACAACGAATCCTGATTTGTCTGCTCTTTGTGGTGGTGAAGATCCTCTTCAATTAGGAGATCATGTTCTGAGTAACTTTGAAGATTGGGATTCTTTGATGAGAGAACTTGGCTTGCACGACGATTCTGCTTCGCTTTCAAAAACGAATCCTCTCACCCACAGGAATCGCTGACTCAGTTCCATAATCTCTCCGAGTTTTCAGCTGAATCGAATCAGTTTCCTAGTCCTGATTTTTCTTTCTCAGACACTAATTTCCCTCAGCAGTTTCCGACGGTGAATCAGGCAAGTTTCATCAACGCCCTTGATCTCTCCGGGGATATCCACCAGAATTGGAGCGTAGGATTTGATTATGTGGATGAACTCATTCGTTTTGCTGAGTGTTTCGAAACAAACGCTTTCCAACTCGCACATGTGATACTGGCACGCCTCAATCAACGGCTCAGATCCGCAGCAGGAAAACCTCTCCAACGATAGCGGCTGGAAAAGTAAAGC
8. GCTTGAAGTGCTTCCTTGAAGTAAAAGGCAGCTCCAACGGTTCTAATTTGTGCTACGAACCTAAGTCAGTGCTCGAGCTCCGTCGTAGCCCAAGTCCTATAGTTGATAAGCAGATTATAACAACGAATCCTGATTTGTCTGCTCTTTGTGGTGGTGAAGATCCTCTTCAATTAGGAGATCATGTTCTGAGTAACTTTGAAGATTGGGATTCTTTGATGAGAGAACTTGGCTTGCACGACGATTCTGCTTCGCTTTCAAAAACGAATCCTCTCACCCACAGGAATCGCTGACTCAGTTCCATAATCTCTCCGAGTTTTCAGCTGAATCGAATCAGTTTCCTAGTCCTGATTTTTCTTTCTCAGACACTAATTTCCCTCAGCAGTTTCCGACGGTGAATCAGGCAAGTTTCATCAACGCCCTTGATCTCTCCGGGGATATCCACCAGAATTGGAGCGTAGGATTTGATTATGTGGATGAACTCATTCGTTTTGCTGAGTGTTTCGAAACAAACGCTTTCCAACTCGCACATGTGATACTGGCACGCCTCAATCAACGGCTCAGATCCGCAGCAGGAAAACCTCTCCAACGATAGCGGCTGGAAAAGTAAAGC
9. GCTTGAAGTGCTTCCTTGAAGTAAAAGGCAGCTCCAACGGTTCTAATTTGTGCTACGAACCTAAGTCAGTGCTCGAGCTCCGTCGTAGCCCAAGTCCTATAGTTGATAAGCAGATTATAACAACGAATCCTGATTTGTCTGCTCTTTGTGGTGGTGAAGATCCTCTTCAATTAGGAGATCATGTTCTGAGTAACTTTGAAGATTGGGATTCTTTGATGAGAGAACTTGGCTTGCACGACGATTCTGCTTCGCTTTCAAAAACGAATCCTCTCACCCACAGGAATCGCTGACTCAGTTCCATAATCTCTCCGAGTTTTCAGCTGAATCGAATCAGTTTCCTAGTCCTGATTTTTCTTTCTCAGACACTAATTTCCCTCAGCAGTTTCCGACGGTGAATCAGGCAAGTTTCATCAACGCCCTTGATCTCTCCGGGGATATCCACCAGAATTGGAGCGTAGGATTTGATTATGTGGATGAACTCATTCGTTTTGCTGAGTGTTTCGAAACAAACGCTTTCCAACTCGCACATGTGATACTGGCACGCCTCAATCAACGGCTCAGATCCGCAGCAGGAAAACCTCTCCAACGATAGCGGCTGGAAAAGTAAAGC
10. GCTTGAAGTGCTTCCTTGAAGTAAAAGGCAGCTCCAACGGTTCTAATTTGTGCTACGAACCTAAGTCAGTGCTCGAGCTCCGTCGTAGCCCAAGTCCTATAGTTGATAAGCAGATTATAACAACGAATCCTGATTTGTCTGCTCTTTGTGGTGGTGAAGATCCTCTTCAATTAGGAGATCATGTTCTGAGTAACTTTGAAGATTGGGATTCTTTGATGAGAGAACTTGGCTTGCACGACGATTCTGCTTCGCTTTCAAAAACGAATCCTCTCACCCACAGGAATCGCTGACTCAGTTCCATAATCTCTCCGAGTTTTCAGCTGAATCGAATCAGTTTCCTAGTCCTGATTTTTCTTTCTCAGACACTAATTTCCCTCAGCAGTTTCCGACGGTGAATCAGGCAAGTTTCATCAACGCCCTTGATCTCTCCGGGGATATCCACCAGAATTGGAGCGTAGGATTTGATTATGTGGATGAACTCATTCGTTTTGCTGAGTGTTTCGAAACAAACGCTTTCCAACTCGCACATGTGATACTGGCACGCCTCAATCAACGGCTCAGATCCGCAGCAGGAAAACCTCTCCAACGATAGCGGCTGGAAAAGTAAAGC
11. GCTTGAAGTGCTTCCTTGAAGTAAAAGGCAGCTCCAACGGTTCTAATTTGTGCTACGAACCTAAGTCAGTGCTCGAGCTCCGTCGTAGCCCAAGTCCTATAGTTGATAAGCAGATTATAACAACGAATCCTGATTTGTCTGCTCTTTGTGGTGGTGAAGATCCTCTTCAATTAGGAGATCATGTTCTGAGTAACTTTGAAGATTGGGATTCTTTGATGAGAGAACTTGGCTTGCACGACGATTCTGCTTCGCTTTCAAAAACGAATCCTCTCACCCACAGGAATCGCTGACTCAGTTCCATAATCTCTCCGAGTTTTCAGCTGAATCGAATCAGTTTCCTAGTCCTGATTTTTCTTTCTCAGACACTAATTTCCCTCAGCAGTTTCCGACGGTGAATCAGGCAAGTTTCATCAACGCCCTTGATCTCTCCGGGGATATCCACCAGAATTGGAGCGTAGGATTTGATTATGTGGATGAACTCATTCGTTTTGCTGAGTGTTTCGAAACAAACGCTTTCCAACTCGCACATGTGATACTGGCACGCCTCAATCAACGGCTCAGATCCGCAGCAGGAAAACCTCTCCAACGATAGCGGCTGGAAAAGTAAAGC
12. GCTTGAAGTGCTTCCTTGAAGTAAAAGGCAGCTCCAACGGTTCTAATTTGTGCTACGAACCTAAGTCAGTGCTCGAGCTCCGTCGTAGCCCAAGTCCTATAGTTGATAAGCAGATTATAACAACGAATCCTGATTTGTCTGCTCTTTGTGGTGGTGAAGATCCTCTTCAATTAGGAGATCATGTTCTGAGTAACTTTGAAGATTGGGATTCTTTGATGAGAGAACTTGGCTTGCACGACGATTCTGCTTCGCTTTCAAAAACGAATCCTCTCACCCACAGGAATCGCTGACTCAGTTCCATAATCTCTCCGAGTTTTCAGCTGAATCGAATCAGTTTCCTAGTCCTGATTTTTCTTTCTCAGACACTAATTTCCCTCAGCAGTTTCCGACGGTGAATCAGGCAAGTTTCATCAACGCCCTTGATCTCTCCGGGGATATCCACCAGAATTGGAGCGTAGGATTTGATTATGTGGATGAACTCATTCGTTTTGCTGAGTGTTTCGAAACAAACGCTTTCCAACTCGCACATGTGATACTGGCACGCCTCAATCAACGGCTCAGATCCGCAGCAGGAAAACCTCTCCAACGATAGCGGCTGGAAAAGTAAAGC
13. GCTTGAAGTGCTTCCTTGAAGTAAAAGGCAGCTCCAACGGTTCTAATTTGTGCTACGAACCTAAGTCAGTGCTCGAGCTCCGTCGTAGCCCAAGTCCTATAGTTGATAAGCAGATTATAACAACGAATCCTGATTTGTCTGCTCTTTGTGGTGGTGAAGATCCTCTTCAATTAGGAGATCATGTTCTGAGTAACTTTGAAGATTGGGATTCTTTGATGAGAGAACTTGGCTTGCACGACGATTCTGCTTCGCTTTCAAAAACGAATCCTCTCACCCACAGGAATCGCTGACTCAGTTCCATAATCTCTCCGAGTTTTCAGCTGAATCGAATCAGTTTCCTAGTCCTGATTTTTCTTTCTCAGACACTAATTTCCCTCAGCAGTTTCCGACGGTGAATCAGGCAAGTTTCATCAACGCCCTTGATCTCTCCGGGGATATCCACCAGAATTGGAGCGTAGGATTTGATTATGTGGATGAACTCATTCGTTTTGCTGAGTGTTTCGAAACAAACGCTTTCCAACTCGCACATGTGATACTGGCACGCCTCAATCAACGGCTCAGATCCGCAGCAGGAAAACCTCTCCAACGATAGCGGCTGGAAAAGTAAAGC
14. GCTTGAAGTGCTTCCTTGAAGTAAAAGGCAGCTCCAACGGTTCTAATTTGTGCTACGAACCTAAGTCAGTGCTCGAGCTCCGTCGTAGCCCAAGTCCTATAGTTGATAAGCAGATTATAACAACGAATCCTGATTTGTCTGCTCTTTGTGGTGGTGAAGATCCTCTTCAATTAGGAGATCATGTTCTGAGTAACTTTGAAGATTGGGATTCTTTGATGAGAGAACTTGGCTTGCACGACGATTCTGCTTCGCTTTCAAAAACGAATCCTCTCACCCACAGGAATCGCTGACTCAGTTCCATAATCTCTCCGAGTTTTCAGCTGAATCGAATCAGTTTCCTAGTCCTGATTTTTCTTTCTCAGACACTAATTTCCCTCAGCAGTTTCCGACGGTGAATCAGGCAAGTTTCATCAACGCCCTTGATCTCTCCGGGGATATCCACCAGAATTGGAGCGTAGGATTTGATTATGTGGATGAACTCATTCGTTTTGCTGAGTGTTTCGAAACAAACGCTTTCCAACTCGCACATGTGATACTGGCACGCCTCAATCAACGGCTCAGATCCGCAGCAGGAAAACCTCTCCAACGATAGCGGCTGGAAAAGTAAAGC
15. GCTTGAAGTGCTTCCTTGAAGTAAAAGGCAGCTCCAACGGTTCTAATTTGTGCTACGAACCTAAGTCAGTGCTCGAGCTCCGTCGTAGCCCAAGTCCTATAGTTGATAAGCAGATTATAACAACGAATCCTGATTTGTCTGCTCTTTGTGGTGGTGAAGATCCTCTTCAATTAGGAGATCATGTTCTGAGTAACTTTGAAGATTGGGATTCTTTGATGAGAGAACTTGGCTTGCACGACGATTCTGCTTCGCTTTCAAAAACGAATCCTCTCACCCACAGGAATCGCTGACTCAGTTCCATAATCTCTCCGAGTTTTCAGCTGAATCGAATCAGTTTCCTAGTCCTGATTTTTCTTTCTCAGACACTAATTTCCCTCAGCAGTTTCCGACGGTGAATCAGGCAAGTTTCATCAACGCCCTTGATCTCTCCGGGGATATCCACCAGAATTGGAGCGTAGGATTTGATTATGTGGATGAACTCATTCGTTTTGCTGAGTGTTTCGAAACAAACGCTTTCCAACTCGCACATGTGATACTGGCACGCCTCAATCAACGGCTCAGATCCGCAGCAGGAAAACCTCTCCAACGATAGCGGCTGGAAAAGTAAAGC
16. GCTTGAAGTGCTTCCTTGAAGTAAAAGGCAGCTCCAACGGTTCTAATTTGTGCTACGAACCTAAGTCAGTGCTCGAGCTCCGTCGTAGCCCAAGTCCTATAGTTGATAAGCAGATTATAACAACGAATCCTGATTTGTCTGCTCTTTGTGGTGGTGAAGATCCTCTTCAATTAGGAGATCATGTTCTGAGTAACTTTGAAGATTGGGATTCTTTGATGAGAGAACTTGGCTTGCACGACGATTCTGCTTCGCTTTCAAAAACGAATCCTCTCACCCACAGGAATCGCTGACTCAGTTCCATAATCTCTCCGAGTTTTCAGCTGAATCGAATCAGTTTCCTAGTCCTGATTTTTCTTTCTCAGACACTAATTTCCCTCAGCAGTTTCCGACGGTGAATCAGGCAAGTTTCATCAACGCCCTTGATCTCTCCGGGGATATCCACCAGAATTGGAGCGTAGGATTTGATTATGTGGATGAACTCATTCGTTTTGCTGAGTGTTTCGAAACAAACGCTTTCCAACTCGCACATGTGATACTGGCACGCCTCAATCAACGGCTCAGATCCGCAGCAGGAAAACCTCTCCAACGATAGCGGCTGGAAAAGTAAAGC
17. GCTTGAAGTGCTTCCTTGAAGTAAAAGGCAGCTCCAACGGTTCTAATTTGTGCTACGAACCTAAGTCAGTGCTCGAGCTCCGTCGTAGCCCAAGTCCTATAGTTGATAAGCAGATTATAACAACGAATCCTGATTTGTCTGCTCTTTGTGGTGGTGAAGATCCTCTTCAATTAGGAGATCATGTTCTGAGTAACTTTGAAGATTGGGATTCTTTGATGAGAGAACTTGGCTTGCACGACGATTCTGCTTCGCTTTCAAAAACGAATCCTCTCACCCACAGGAATCGCTGACTCAGTTCCATAATCTCTCCGAGTTTTCAGCTGAATCGAATCAGTTTCCTAGTCCTGATTTTTCTTTCTCAGACACTAATTTCCCTCAGCAGTTTCCGACGGTGAATCAGGCAAGTTTCATCAACGCCCTTGATCTCTCCGGGGATATCCACCAGAATTGGAGCGTAGGATTTGATTATGTGGATGAACTCATTCGTTTTGCTGAGTGTTTCGAAACAAACGCTTTCCAACTCGCACATGTGATACTGGCACGCCTCAATCAACGGCTCAGATCCGCAGCAGGAAAACCTCTCCAACGATAGCGGCTGGAAAAGTAAAGC
18. GCTTGAAGTGCTTCCTTGAAGTAAAAGGCAGCTCCAACGGTTCTAATTTGTGCTACGAACCTAAGTCAGTGCTCGAGCTCCGTCGTAGCCCAAGTCCTATAGTTGATAAGCAGATTATAACAACGAATCCTGATTTGTCTGCTCTTTGTGGTGGTGAAGATCCTCTTCAATTAGGAGATCATGTTCTGAGTAACTTTGAAGATTGGGATTCTTTGATGAGAGAACTTGGCTTGCACGACGATTCTGCTTCGCTTTCAAAAACGAATCCTCTCACCCACAGGAATCGCTGACTCAGTTCCATAATCTCTCCGAGTTTTCAGCTGAATCGAATCAGTTTCCTAGTCCTGATTTTTCTTTCTCAGACACTAATTTCCCTCAGCAGTTTCCGACGGTGAATCAGGCAAGTTTCATCAACGCCCTTGATCTCTCCGGGGATATCCACCAGAATTGGAGCGTAGGATTTGATTATGTGGATGAACTCATTCGTTTTGCTGAGTGTTTCGAAACAAACGCTTTCCAACTCGCACATGTGATACTGGCACGCCTCAATCAACGGCTCAGATCCGCAGCAGGAAAACCTCTCCAACGATAGCGGCTGGAAAAGTAAAGC
19. GCTTGAAGTGCTTCCTTGAAGTAAAAGGCAGCTCCAACGGTTCTAATTTGTGCTACGAACCTAAGTCAGTGCTCGAGCTCCGTCGTAGCCCAAGTCCTATAGTTGATAAGCAGATTATAACAACGAATCCTGATTTGTCTGCTCTTTGTGGTGGTGAAGATCCTCTTCAATTAGGAGATCATGTTCTGAGTAACTTTGAAGATTGGGATTCTTTGATGAGAGAACTTGGCTTGCACGACGATTCTGCTTCGCTTTCAAAAACGAATCCTCTCACCCACAGGAATCGCTGACTCAGTTCCATAATCTCTCCGAGTTTTCAGCTGAATCGAATCAGTTTCCTAGTCCTGATTTTTCTTTCTCAGACACTAATTTCCCTCAGCAGTTTCCGACGGTGAATCAGGCAAGTTTCATCAACGCCCTTGATCTCTCCGGGGATATCCACCAGAATTGGAGCGTAGGATTTGATTATGTGGATGAACTCATTCGTTTTGCTGAGTGTTTCGAAACAAACGCTTTCCAACTCGCACATGTGATACTGGCACGCCTCAATCAACGGCTCAGATCCGCAGCAGGAAAACCTCTCCAACGATAGCGGCTGGAAAAGTAAAGC

## Text S7. Monoclonal sequencing for assembly of multiple sgRNA expression cassettes

Target site is written in red.

**Two sgRNA expression cassettes:** AtU3b:ACS2-AtU626:ACS4

AGTTTATTCCCGAAAGGTTCTCGGGTACCCCGTCTAGAGGCTCGAGCCTTTGGAATCGGCAGCAAAGGATTTACTTTAAATTTTTTCTTATGCAGCCTGTGATGGATAACTGAATCAAACAAATGGCGTCTGGGTTTAAGAAGATCTGTTTTGGCTATGTTGGACGAAACAAGTGAACTTTTAGGATCAACTTCAGTTTATATATGGAGCTTATATCGAGCAATAAGATAAGTGGGCTTTTTATGTAATTTAATGGGCTATCGTCCATAGATTCACTAATACCCATGCCCAGTACCCATGTATGCGTTTCATATAAGCTCCTAATTTCTCCCACATCGCTCAAATCTAAACAAATCTTGTTGTATATATAACACTGAGGGAGCAACATTGGTCACACCCTCTAAAAAACCCCAAGTTTTAGAGCTAGAAATAGCAAGTTAAAATAAGGCTAGTCCGTTATCAACTTGAAAAAGTGGCACCGAGTCGGTGCTTTTTTTCAAGAGCTTGGAGTGGATGGAAAATGCCTTTGGAATCGGCAGCAAAGGAAGCTTTCGTTTTCTTCTTTTTAACTTTCCATTCGGAGTTTTTGTATCTTGTTTCATAGTTTGTCCCAGGATTAGAATGATTAGGCATCGAACCTTCAAGAATTTGATTGAATAAAACATCTTCATTCTTAAGATATGAAGATAATCTTCAAAAGGCCCCTGGGAATCTGAAAGAAGAGAAGCAGGCCCATTTATATGGGAAAGAACAATAGTATTTCTTATATAGGCCCATTTAAGTTGAAAACAATCTTCAAAAGTCCCACATCGCTTAGATAAGAAAACGAAGCTGAGTTTATATACAGCTAGAGTCGAAGTAGTGATTGCGATAACGACCCTTTCCACTGTTTTAGAGCTAGAAATAGCAAGTTAAAATAAGGCTAGTCCGTTATCAACTTGAAAAAGTGGCACCGAGTCGGTGCTTTTTTTCAAGAGCTTGGAGTGGATGGAAACTAGTGCTGTCGTCTGACATCCA

**Three sgRNA expression cassettes:** AtU3b:ACS1- AtU3d:ACS3-AtU626:ACS4

AACACTGATAGTTTAATTCCCGAATCGGCTCGGGTACCCCGTCTAGAGGCTCGAGCCTTTGGAATCGGCAGCAAAGGATTTACTTTAAATTTTTTCTTATGCAGCCTGTGATGGATAACTGAATCAAACAAATGGCGTCTGGGTTTAAGAAGATCTGTTTTGGCTATGTTGGACGAAACAAGTGAACTTTTAGGATCAACTTCAGTTTATATATGGAGCTTATATCGAGCAATAAGATAAGTGGGCTTTTTATGTAATTTAATGGGCTATCGTCCATAGATTCACTAATACCCATGCCCAGTACCCATGTATGCGTTTCATATAAGCTCCTAATTTCTCCCACATCGCTCAAATCTAAACAAATCTTGTTGTATATATAACACTGAGGGAGCAACATTGGTCAGGTCTTGCTGAAAATCAGGTGTTTTAGAGCTAGAAATAGCAAGTTAAAATAAGGCTAGTCCGTTATCAACTTGAAAAAGTGGCACCGAGTCGGTGCTTTTTTTCAAGAGCTTGGAGTGGATGGAAAATGCCTTTGGAATCGGCAGCAAAGGAATAAGCTTATGATTTCTTTTTTCTTACGAATTTTGCGTCCCACATCGGTAAGCGAGTGAAGAAATAACTGCTTTATATATGGCTACAAAGCACCATTGGTCACCTCAAATGGAGAACTGGGGGTTTTAGAGCTAGAAATAGCAAGTTAAAATAAGGCTAGTCCGTTATCAACTTGAAAAAGTGGCACCGAGTCGGTGCTTTTTTTCAAGAGCTTGGAGTGGATGGAAATAGCCTTTGGAATCGGCAGCAAAGGAAGCTTTCGTTTTCTTCTTTTTAACTTTCCATTCGGAGTTTTTGTATCTTGTTTCATAGTTTGTCCCAGGATTAGAATGATTAGGCATCGAACCTTCAAGAATTTGATTGAATAAAACATCTTCATTCTTAAGATATGAAGATAATCTTCAAAAGGCCCCTGGGAATCTGAAAGAAGAGAAGCAGGCCCATTTATATGGGAAAGAACAATAGTATTTCTTATATAGGCCCATTTAAGTTGAAAACAATCTTCAAAAGTCCCACATCGCTTAGATAAGAAAACGAAGCTGAGTTTATATACAGCTAGAGTCGAAGTAGTGATTGCGATAACGACCCTTTCCACTGTTTTAGAGCTAGAAATAGCAAGTTAAAATAAGGCTAGTCCGTTATCAACTTGAAAAAGTGGCACCGAGTCGGTGCTTTTTTTCAAGAGCTTGGAGTGGATGGAAACTAGTGCTGTCGTCTCACACCCAC

**Four sgRNA expression cassettes:** AtU3b:ACS1-AtU3b:ACS2- AtU3d:ACS3-AtU626:ACS4

TATCCTGTCAAACACTGATAGTTTAATTCCCGAAAGCTTCTCGGGTACCCCGTCTAGAGGCTCGAGCCTTTGGAATCGGCAGCAAAGGATTTACTTTAAATTTTTTCTTATGCAGCCTGTGATGGATAACTGAATCAAACAAATGGCGTCTGGGTTTAAGAAGATCTGTTTTGGCTATGTTGGACGAAACAAGTGAACTTTTAGGATCAACTTCAGTTTATATATGGAGCTTATATCGAGCAATAAGATAAGTGGGCTTTTTATGTAATTTAATGGGCTATCGTCCATAGATTCACTAATACCCATGCCCAGTACCCATGTATGCGTTTCATATAAGCTCCTAATTTCTCCCACATCGCTCAAATCTAAACAAATCTTGTTGTATATATAACACTGAGGGAGCAACATTGGTCAGGTCTTGCTGAAAATCAGGTGTTTTAGAGCTAGAAATAGCAAGTTAAAATAAGGCTAGTCCGTTATCAACTTGAAAAAGTGGCACCGAGTCGGTGCTTTTTTTCAAGAGCTTGGAGTGGATGGAAAATGCCTTTGGAATCGGCAGCAAAGGATTTACTTTAAATTTTTTCTTATGCAGCCTGTGATGGATAACTGAATCAAACAAATGGCGTCTGGGTTTAAGAAGATCTGTTTTGGCTATGTTGGACGAAACAAGTGAACTTTTAGGATCAACTTCAGTTTATATATGGAGCTTATATCGAGCAATAAGATAAGTGGGCTTTTTATGTAATTTAATGGGCTATCGTCCATAGATTCACTAATACCCATGCCCAGTACCCATGTATGCGTTTCATATAAGCTCCTAATTTCTCCCACATCGCTCAAATCTAAACAAATCTTGTTGTATATATAACACTGAGGGAGCAACATTGGTCACACCCTCTAAAAAACCCCAAGTTTTAGAGCTAGAAATAGCAAGTTAAAATAAGGCTAGTCCGTTATCAACTTGAAAAAGTGGCACCGAGTCGGTGCTTTTTTTCAAGAGCTTGGAGTGGATGGAAATAGCCTTTGGAATCGGCAGCAAAGGAATAAGCTTATGATTTCTTTTTTCTTACGAATTTTGCGTCCCACATCGGTAAGCGAGTGAAGAAATAACTGCTTTATATATGGCTACAAAGCACCATTGGTCACCTCAAATGGAGAACTGGGGGTTTTAGAGCTAGAAATAGCAAGTTAAAATAAGGCTAGTCCGTTATCAACTTGAAAAAGTGGCACCGAGTCGGTGCTTTTTTTCAAGAGCTTGGAGTGGATGGAAAGGGCCTTTGGAATCGGCAGCAAAGGAAGCTTTCGTTTTCTTCTTTTTAACTTTCCATTCGGAGTTTTTGTATCTTGTTTCATAGTTTGTCCCAGGATTAGAATGATTAGGCATCGAACCTTCAAGAATTTGATTGAATAAAACATCTTCATTCTTAAGATATGAAGATAATCTTCAAAAGGCCCCTGGGAATCTGAAAGAAGAGAAGCAGGCCCATTTATATGGGAAAGAACAATAGTATTTCTTATATAGGCCCATTTAAGTTGAAAACAATCTTCAAAAGTCCCACATCGCTTAGATAAGAAAACGAAGCTGAGTTTATATACAGCTAGAGTCGAAGTAGTGATTGCGATAACGACCCTTTCCACTGTTTTAGAGCTAGAAATAGCAAGTTAAAATAAGGCTAGTCCGTTATCAACTTGAAAAAGTGGCACCGAGTCGGTGCTTTTTTTCAAGAGCTTGGAGTGGATGGAAACTAGTGCTGTCGTCTCACCATCAC

**Five sgRNA expression cassettes:** AtU3b:ACS1-AtU3b:ACS2-AtU3d:ACS3-AtU3d:ACS5-AtU626:ACS4

AGGATTTACTTTAAATTTTTTCTTATGCAGCCTGTGATGGATAACTGAATCAAACAAATGGCGTCTGGGTTTAAGAAGATCTGTTTTGGCTATGTTGGACGAAACAAGTGAACTTTTAGGATCAACTTCAGTTTATATATGGAGCTTATATCGAGCAATAAGATAAGTGGGCTTTTTATGTAATTTAATGGGCTATCGTCCATAGATTCACTAATACCCATGCCCAGTACCCATGTATGCGTTTCATATAAGCTCCTAATTTCTCCCACATCGCTCAAATCTAAACAAATCTTGTTGTATATATAACACTGAGGGAGCAACATTGGTCAGGTCTTGCTGAAAATCAGGTGTTTTAGAGCTAGAAATAGCAAGTTAAAATAAGGCTAGTCCGTTATCAACTTGAAAAAGTGGCACCGAGTCGGTGCTTTTTTTCAAGAGCTTGGAGTGGATGGAAAATGCCTTTGGAATCGGCAGCAAAGGATTTACTTTAAATTTTTTCTTATGCAGCCTGTGATGGATAACTGAATCAAACAAATGGCGTCTGGGTTTAAGAAGATCTGTTTTGGCTATGTTGGACGAAACAAGTGAACTTTTAGGATCAACTTCAGTTTATATATGGAGCTTATATCGAGCAATAAGATAAGTGGGCTTTTTATGTAATTTAATGGGCTATCGTCCATAGATTCACTAATACCCATGCCCAGTACCCATGTATGCGTTTCATATAAGCTCCTAATTTCTCCCACATCGCTCAAATCTAAACAAATCTTGTTGTATATATAACACTGAGGGAGCAACATTGGTCACACCCTCTAAAAAACCCCAAGTTTTAGAGCTAGAAATAGCAAGTTAAAATAAGGCTAGTCCGTTATCAACTTGAAAAAGTGGCACCGAGTCGGTGCTTTTTTTCAAGAGCTTGGAGTGGATGGAAATAGCCTTTGGAATCGGCAGCAAAGGAATAAGCTTATGATTTCTTTTTCTTACGAATTTTGCGTCCCACATCGGTAAGCGAGTGAAGAAATAACTGCTTTATATATGGCTACAAAGCACCATTGGTCACCTCAAATGGAGAACTGGGGGTTTTAGAGCTAGAAATAGCAAGTTAAAATAAGGCTAGTCCGTTATCAACTTGAAAAAGTGGCACCGAGTCGGTGCTTTTTTTCAAGAGCTTGGAGTGGATGGAAAGGGCCTTTGGAATCGGCAGCAAAGGAATAAGCTTATGATTTCTTTTTTCTTACGAATTTTGCGTCCCACATCGGTAAGCGAGTGAAGAAATAACTGCTTTATATATGGCTACAAAGCACCATTGGTCAATAAGAAGAATCTTGCCCATGTTTTAGAGCTAGAAATAGCAAGTTAAAATAAGGCTAGTCCGTTATCAACTTGAAAAAGTGGCACCGAGTCGGTGCTTTTTTTCAAGAGCTTGGAGTGGATGGAATATCCCTTTGGAATCGGCAGCAAAGGAAGCTTTCGTTTTCTTCTTTTTAACTTTCCATTCGGAGTTTTTGTATCTTGTTTCATAGTTTGTCCCAGGATTAGAATGATTAGGCATCGAACCTTCAAGAATTTGATTGAATAAAACATCTTCATTCTTAAGATATGAAGATAATCTTCAAAAGGCCCCTGGGAATCTGAAAGAAGAGAAGCAGGCCCATTTATATGGGAAAGAACAATAGTATTTCTTATATAGGCCCATTTAAGTTGAAAACAATCTTCAAAAGTCCCACATCGCTTAGATAAGAAAACGAAGCTGAGTTTATATACAGCTAGAGTCGAAGTAGTGATTGCGATAACGACCCTTTCCACTGTTTTAGAGCTAGAAATAGCAAGTTAAAATAAGGCTAGTCCGTTATCAACTTGAAAAAGTGGCACCGAGTCGGTGCTTTTTTTCAAGAGCTTGGAGTGGATGGAAACTAGTGCTGTCGTCTCACCCCCCC

**Six sgRNA expression cassettes:** AtU3b:ACS1-AtU3b:ACS2-AtU3d:ACS3-AtU3d:ACS5-AtU626:ACS4-AtU626:ACS6

CTTAGGTTTACCCGCCAATATATCCTGTCAAACACTGATAGTTTAATTCCCGAAAGCTTCTCGGGTACCCCGTCTAGAGGCTCGAGCCTTTGGAATCGGCAGCAAAGGATTTACTTTAAATTTTTTCTTATGCAGCCTGTGATGGATAACTGAATCAAACAAATGGCGTCTGGGTTTAAGAAGATCTGTTTTGGCTATGTTGGACGAAACAAGTGAACTTTTAGGATCAACTTCAGTTTATATATGGAGCTTATATCGAGCAATAAGATAAGTGGGCTTTTTATGTAATTTAATGGGCTATCGTCCATAGATTCACTAATACCCATGCCCAGTACCCATGTATGCGTTTCATATAAGCTCCTAATTTCTCCCACATCGCTCAAATCTAAACAAATCTTGTTGTATATATAACACTGAGGGAGCAACATTGGTCAGGTCTTGCTGAAAATCAGGTGTTTTAGAGCTAGAAATAGCAAGTTAAAATAAGGCTAGTCCGTTATCAACTTGAAAAAGTGGCACCGAGTCGGTGCTTTTTTTCAAGAGCTTGGAGTGGATGGAAAATGCCTTTGGAATCGGCAGCAAAGGATTTACTTTAAATTTTTTCTTATGCAGCCTGTGATGGATAACTGAATCAAACAAATGGCGTCTGGGTTTAAGAAGATCTGTTTTGGCTATGTTGGACGAAACAAGTGAACTTTTAGGATCAACTTCAGTTTATATATGGAGCTTATATCGAGCAATAAGATAAGTGGGCTTTTTATGTAATTTAATGGGCTATCGTCCATAGATTCACTAATACCCATGCCCAGTACCCATGTATGCGTTTCATATAAGCTCCTAATTTCTCCCACATCGCTCAAATCTAAACAAATCTTGTTGTATATATAACACTGAGGGAGCAACATTGGTCACACCCTCTAAAAAACCCCAAGTTTTAGAGCTAGAAATAGCAAGTTAAAATAAGGCTAGTCCGTTATCAACTTGAAAAAGTGGCACCGAGTCGGTGCTTTTTTTCAAGAGCTTGGAGTGGATGGAAATAGCCTTTGGAATCGGCAGCAAAGGAATAAGCTTATGATTTCTTTTTCTTACGAATTTTGCGTCCCACATCGGTAAGCGAGTGAAGAAATAACTGCTTTATATATGGCTACAAAGCACCATTGGTCACCTCAAATGGAGAACTGGGGGTTTTAGAGCTAGAAATAGCAAGTTAAAATAAGGCTAGTCCGTTATCAACTTGAAAAAGTGGCACCGAGTCGGTGCTTTTTTTCAAGAGCTTGGAGTGGATGGAAAGGGCCTTTGGAATCGGCAGCAAAGGAATAAGCTTATGATTTCTTTTTTCTTACGAATTTTGCGTCCCACATCGGTAAGCGAGTGAAGAAATAACTGCTTTATATATGGCTACAAAGCACCATTGGTCAATAAGAAGAATCTTGCCCATGTTTTAGAGCTAGAAATAGCAAGTTAAAATAAGGCTAGTCCGTTATCAACTTGAAAAAGTGGCACCGAGTCGGTGCTTTTTTTCAAGAGCTTGGAGTGGATGGAATATCCCTTTGGAATCGGCAGCAAAGGAAGCTTTCGTTTTCTTCTTTTTAACTTTCCATTCGGAGTTTTTGTATCTTGTTTCATAGTTTGTCCCAGGATTAGAATGATTAGGCATCGAACCTTCAAGAATTTGATTGAATAAAACATCTTCATTCTTAAGATATGAAGATAATCTTCAAAAGGCCCCTGGGAATCTGAAAGAAGAGAAGCAGGCCCATTTATATGGGAAAGAACAATAGTATTTCTTATATAGGCCCATTTAAGTTGAAAACAATCTTCAAAAGTCCCACATCGCTTAGATAAGAAAACGAAGCTGAGTTTATATACAGCTAGAGTCGAAGTAGTGATTGCGATAACGACCCTTTCCACTGTTTTAGAGCTAGAAATAGCAAGTTAAAATAAGGCTAGTCCGTTATCAACTTGAAAAAGTGGCACCGAGTCGGTGCTTTTTTTCAAGAGCTTGGAGTGGATGGAATTACCCTTTGGAATCGGCAGCAAAGGAAGCTTTCGTTTTCTTCTTTTTAACTTTCCATTCGGAGTTTTTGTATCTTGTTTCATAGTTTGTCCCAGGATTAGAATGATTAGGCATCGAACCTTCAAGAATTTGATTGAATAAAACATCTTCATTCTTAAGATATGAAGATAATCTTCAAAAGGCCCCTGGGAATCTGAAAGAAGAGAAGCAGGCCCATTTATATGGGAAAGAACAATAGTATTTCTTATATAGGCCCATTTAAGTTGAAAACAATCTTCAAAAGTCCCACATCGCTTAGATAAGAAAACGAAGCTGAGTTTATATACAGCTAGAGTCGAAGTAGTGATTGTTAGAACTCTCACAAACAACGTTTTAGAGCTAGAAATAGCAAGTTAAAATAAGGCTAGTCCGTTATCAACTTGAAAAAGTGGCACCGAGTCGGTGCTTTTTTTCAAGAGCTTGGAGTGGATGGAAACTAGTGCTGTCGTCTCACTCCC

**Seven sgRNA expression cassettes:** AtU3b:ACS1-AtU3b:ACS2-AtU3d:ACS3-AtU3d:ACS5-AtU3d:ACS7-AtU626:ACS4-AtU626:ACS6

CGGCAGCAAAGGATTTACTTTAAATTTTTTCTTATGCAGCCTGTGATGGATAACTGAATCAAACAAATGGCGTCTGGGTTTAAGAAGATCTGTTTTGGCTATGTGGACGAAACAAGTGAACTTTTAGGATCAACTTCAGTTTATATATGGAGCTTATATCGAGCAATAAGATAAGTGGGCTTTTTATGTAATTTAATGGGCTATCGTCCATAGATTCACTAATACCCATGCCCAGTACCCATGTATGCGTTTCATATAAGCTCCTAATTTCTCCCACATCGCTCAAATCTAAACAAATCTTGTTGTATATATAACACTGAGGGAGCAACATTGGTCAGGTCTTGCTGAAAATCAGGTGTTTTAGAGCTAGAAATAGCAAGTTAAAATAAGGCTAGTCCGTTATCAACTTGAAAAAGTGGCACCGAGTCGGTGCTTTTTTTCAAGAGCTTGGAGTGGATGGAAAATGCCTTTGGAATCGGCAGCAAAGGATTTACTTTAAATTTTTTCTTATGCAGCCTGTGATGGATAACTGAATCAAACAAATGGCGTCTGGGTTTAAGAAGATCTGTTTTGGCTATGTTGGACGAAACAAGTGAACTTTTAGGATCAACTTCAGTTTATATATGGAGCTTATATCGAGCAATAAGATAAGTGGGCTTTTTATGTAATTTAATGGGCTATCGTCCATAGATTCACTAATACCCATGCCCAGTACCCATGTATGCGTTTCATATAAGCTCCTAATTTCTCCCACATCGCTCAAATCTAAACAAATCTTGTTGTATATATAACACTGAGGGAGCAACATTGGTCACACCCTCTAAAAAACCCCAAGTTTTAGAGCTAGAAATAGCAAGTTAAAATAAGGCTAGTCCGTTATCAACTTGAAAAAGTGGCACCGAGTCGGTGCTTTTTTTCAAGAGCTTGGAGTGGATGGAAATAGCCTTTGGAATCGGCAGCAAAGGAATAAGCTTATGATTTCTTTTTCTTACGAATTTTGCGTCCCACATCGGTAAGCGAGTGAAGAAATAACTGCTTTATATATGGCTACAAAGCACCATTGGTCACCTCAAATGGAGAACTGGGGGTTTTAGAGCTAGAAATAGCAAGTTAAAATAAGGCTAGTCCGTTATCAACTTGAAAAAGTGGCACCGAGTCGGTGCTTTTTTTCAAGAGCTTGGAGTGGATGGAAAGGGCCTTTGGAATCGGCAGCAAAGGAATAAGCTTATGATTTCTTTTTTCTTACGAATTTTGCGTCCCACATCGGTAAGCGAGTGAAGAAATAACTGCTTTATATATGGCTACAAAGCACCATTGGTCAATAAGAAGAATCTTGCCCATGTTTTAGAGCTAGAAATAGCAAGTTAAAATAAGGCTAGTCCGTTATCAACTTGAAAAAGTGGCACCGAGTCGGTGCTTTTTTTCAAGAGCTTGGAGTGGATGGAATATCCCTTTGGAATCGGCAGCAAAGGAATAAGCTTATGATTTCTTTTTTCTTACGAATTTTGCGTCCCACATCGGTAAGCGAGTGAAGAAATAACTGCTTTATATATGGCTACAAAGCACCATTGGTCAAGCATGGCCAGGATCAGCGGTTTTAGAGCTAGAAATAGCAAGTTAAAATAAGGCTAGTCCGTTATCAACTTGAAAAAGTGGCACCGAGTCGGTGCTTTTTTTCAAGAGCTTGGAGTGGATGGAATTACCCTTTGGAATCGGCAGCAAAGGAAGCTTTCGTTTTCTTCTTTTTAACTTTCCATTCGGAGTTTTTGTATCTTGTTTCATAGTTTGTCCCAGGATTAGAATGATTAGGCATCGAACCTTCAAGAATTTGATTGAATAAAACATCTTCATTCTTAAGATATGAAGATAATCTTCAAAAGGCCCCTGGGAATCTGAAAGAAGAGAAGCAGGCCCATTTATATGGGAAAGAACAATAGTATTTCTTATATAGGCCCATTTAAGTTGAAAACAATCTTCAAAAGTCCCACATCGCTTAGATAAGAAAACGAAGCTGAGTTTATATACAGCTAGAGTCGAAGTAGTGATTGCGATAACGACCCTTTCCACTGTTTTAGAGCTAGAAATAGCAAGTTAAAATAAGGCTAGTCCGTTATCAACTTGAAAAAGTGGCACCGAGTCGGTGCTTTTTTTCAAGAGCTTGGAGTGGATGGAATCGCCCTTTGGAATCGGCAGCAAAGGAAGCTTTCGTTTTCTTCTTTTTAACTTTCCATTCGGAGTTTTTGTATCTTGTTTCATAGTTTGTCCCAGGATTAGAATGATTAGGCATCGAACCTTCAAGAATTTGATTGAATAAAACATCTTCATTCTTAAGATATGAAGATAATCTTCAAAAGGCCCCTGGGAATCTGAAAGAAGAGAAGCAGGCCCATTTATATGGGAAAGAACAATAGTATTTCTTATATAGGCCCATTTAAGTTGAAAACAATCTTCAAAAGTCCCACATCGCTTAGATAAGAAAACGAAGCTGAGTTTATATACAGCTAGAGTCGAAGTAGTGATTGTTAGAACTCTCACAAACAACGTTTTAGACTAGAAATAGCAAGTTAAAATAAGGCTAGTCCGTTATCAACTTGAAAAAGTGGCACCGAGTCGGTGCTTTTTTTCAAGAGCTTGGAGTGGATGGAACTAGTGCTGTCGTCTCACCACCCCC

**Eight sgRNA expression cassettes:** AtU3b:ACS1-AtU3b:ACS2-AtU3d:ACS3-AtU3d:ACS5-AtU3d:ACS7-AtU626:ACS4-AtU626:ACS6--AtU626:ACS8

TAATAAACGCTCTTTTCTCTTAGGTTTACCCGCCAATATATCCTGTCAAACACTGATAGTTTAATTCCCGAAAGCTTCTCGGGTACCCCGTCTAGAGGCTCGAGCCTTTGGAATCGGCAGCAAAGGATTTACTTTAAATTTTTTCTTATGCAGCCTGTGATGGATAACTGAATCAAACAAATGGCGTCTGGGTTTAAGAAGATCTGTTTTGGCTATGTTGGACGAAACAAGTGAACTTTTAGGATCAACTTCAGTTTATATATGGAGCTTATATCGAGCAATAAGATAAGTGGGCTTTTTATGTAATTTAATGGGCTATCGTCCATAGATTCACTAATACCCATGCCCAGTACCCATGTATGCGTTTCATATAAGCTCCTAATTTCTCCCACATCGCTCAAATCTAAACAAATCTTGTTGTATATATAACACTGAGGGAGCAACATTGGTCAGGTCTTGCTGAAAATCAGGTGTTTTAGAGCTAGAAATAGCAAGTTAAAATAAGGCTAGTCCGTTATCAACTTGAAAAAGTGGCACCGAGTCGGTGCTTTTTTTCAAGAGCTTGGAGTGGATGGAAAATGCCTTTGGAATCGGCAGCAAAGGATTTACTTTAAATTTTTTCTTATGCAGCCTGTGATGGATAACTGAATCAAACAAATGGCGTCTGGGTTTAAGAAGATCTGTTTTGGCTATGTTGGACGAAACAAGTGAACTTTTAGGATCAACTTCAGTTTATATATGGAGCTTATATCGAGCAATAAGATAAGTGGGCTTTTTATGTAATTTAATGGGCTATCGTCCATAGATTCACTAATACCCATGCCCAGTACCCATGTATGCGTTTCATATAAGCTCCTAATTTCTCCCACATCGCTCAAATCTAAACAAATCTTGTTGTATATATAACACTGAGGGAGCAACATTGGTCACACCCTCTAAAAAACCCCAAGTTTTAGAGCTAGAAATAGCAAGTTAAAATAAGGCTAGTCCGTTATCAACTTGAAAAAGTGGCACCGAGTCGGTGCTTTTTTTCAAGAGCTTGGAGTGGATGGAAATAGCCTTTGGAATCGGCAGCAAAGGAATAAGCTTATGATTTCTTTTTTCTTACGAATTTTGCGTCCCACATCGGTAAGCGAGTGAAGAAATAACTGCTTTATATATGGCTACAAAGCACCATTGGTCACCTCAAATGGAGAACTGGGGGTTTTAGAGCTAGAAATAGCAAGTTAAAATAAGGCTAGTCCGTTATCAACTTGAAAAAGTGGCACCGAGTCGGTGCTTTTTTTCAAGAGCTTGGAGTGGATGGAAAGGGCCTTTGGAATCGGCAGCAAAGGAATAAGCTTATGATTTCTTTTTTCTTACGAATTTTGCGTCCCACATCGGTAAGCGAGTGAAGAAATAACTGCTTTATATATGGCTACAAAGCACCATTGGTCAATAAGAAGAATCTTGCCCATGTTTTAGAGCTAGAAATAGCAAGTTAAAATAAGGCTAGTCCGTTATCAACTTGAAAAAGTGGCACCGAGTCGGTGCTTTTTTTCAAGAGCTTGGAGTGGATGGAATATCCCTTTGGAATCGGCAGCAAAGGAATAAGCTTATGATTTCTTTTTTCTTACGAATTTTGCGTCCCACATCGGTAAGCGAGTGAAGAAATAACTGCTTTATATATGGCTACAAAGCACCATTGGTCAAGCATGGCCAGGATCAGCGGTTTTAGAGCTAGAAATAGCAAGTTAAAATAAGGCTAGTCCGTTATCAACTTGAAAAAGTGGCACCGAGTCGGTGCTTTTTTTCAAGAGCTTGGAGTGGATGGAATTACCCTTTGGAATCGGCAGCAAAGGAAGCTTTCGTTTTCTTCTTTTTAACTTTCCATTCGGAGTTTTTGTATCTTGTTTCATAGTTTGTCCCAGGATTAGAATGATTAGGCATCGAACCTTCAAGAATTTGATTGAATAAAACATCTTCATTCTTAAGATATGAAGATAATCTTCAAAAGGCCCCTGGGAATCTGAAAGAAGAGAAGCAGGCCCATTTATATGGGAAAGAACAATAGTATTTCTTATATAGGCCCATTTAAGTTGAAAACAATCTTCAAAAGTCCCACATCGCTTAGATAAGAAAACGAAGCTGAGTTTATATACAGCTAGAGTCGAAGTAGTGATTGCGATAACGACCCTTTCCACTGTTTTAGAGCTAGAAATAGCAAGTTAAAATAAGGCTAGTCCGTTATCAACTTGAAAAAGTGGCACCGAGTCGGTGCTTTTTTTCAAGAGCTTGGAGTGGATGGAATCGCCCTTTGGAATCGGCAGCAAAGGAAGCTTTCGTTTTCTTCTTTTTAACTTTCCATTCGGAGTTTTTGTATCTTGTTTCATAGTTTGTCCCAGGATTAGAATGATTAGGCATCGAACCTTCAAGAATTTGATTGAATAAAACATCTTCATTCTTAAGATATGAAGATAATCTTCAAAAGGCCCCTGGGAATCTGAAAGAAGAGAAGCAGGCCCATTTATATGGGAAAGAACAATAGTATTTCTTATATAGGCCCATTTAAGTTGAAAACAATCTTCAAAAGTCCCACATCGCTTAGATAAGAAAACGAAGCTGAGTTTATATACAGCTAGAGTCGAAGTAGTGATTGTTAGAACTCTCACAAACAACGTTTTAGAGCTAGAAATAGCAAGTTAAAATAAGGCTAGTCCGTTATCAACTTGAAAAAGTGGCACCGAGTCGGTGCTTTTTTTCAAGAGCTTGGAGTGGATGGAATGCCCCTTTGGAATCGGCAGCAAAGGAAGCTTTCGTTTTCTTCTTTTTAACTTTCCATTCGGAGTTTTTGTATCTTGTTTCATAGTTTGTCCCAGGATTAGAATGATTAGGCATCGAACCTTCAAGAATTTGATTGAATAAAACATCTTCATTCTTAAGATATGAAGATAATCTTCAAAAGGCCCCTGGGAATCTGAAAGAAGAGAAGCAGGCCCATTTATATGGGAAAGAACAATAGTATTTCTTATATAGGCCCATTTAAGTTGAAAACAATCTTCAAAAGTCCCACATCGCTTAGATAAGAAAACGAAGCTGAGTTTATATACAGCTAGAGTCGAAGTAGTGATTGTTCCTACCCCTTACTACCCGGTTTTAGAGCTAGAAATAGCAAGTTAAAATAAGGCTAGTCCGTTATCAACTTGAAAAAGTGGCACCGAGTCGGTGCTTTTTTTCAAGAGCTTGGAGTGGATGGAAACTAGTGCTGTCGTCGACACCAC

##
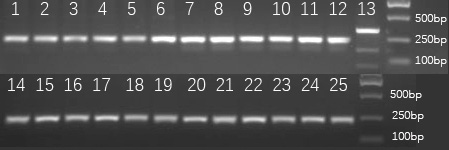
Figure S1. Off-target detection by T7EI assay in T1 plant

**1-6:** T7E1 digestion of the off-target 1 PCR fragments come from T1 plant of L1, L2, L3, L4, L5 and WT plant. The off-sorce=0.468. No off-target events were found.

**7-12:** T7E1 digestion of the off-target 2 PCR fragments come from T1 plant of L1, L2, L3, L4, L5 and WT plant. The off-sorce=0.323. No off-target events were found.

**13:** T7E1 digestion of the Positive Control.

**14-19:** T7E1 digestion of the off-target 3 PCR fragments come from T1 plant of L1, L2, L3, L4, L5 and WT plant. The off-sorce=0.240. No off-target events were found.

**20-25:** T7E1 digestion of the off-target 4 PCR fragments come from T1 plant of L1, L2, L3, L4, L5 and WT plant. The off-sorce=0.233. No off-target events were found.

##
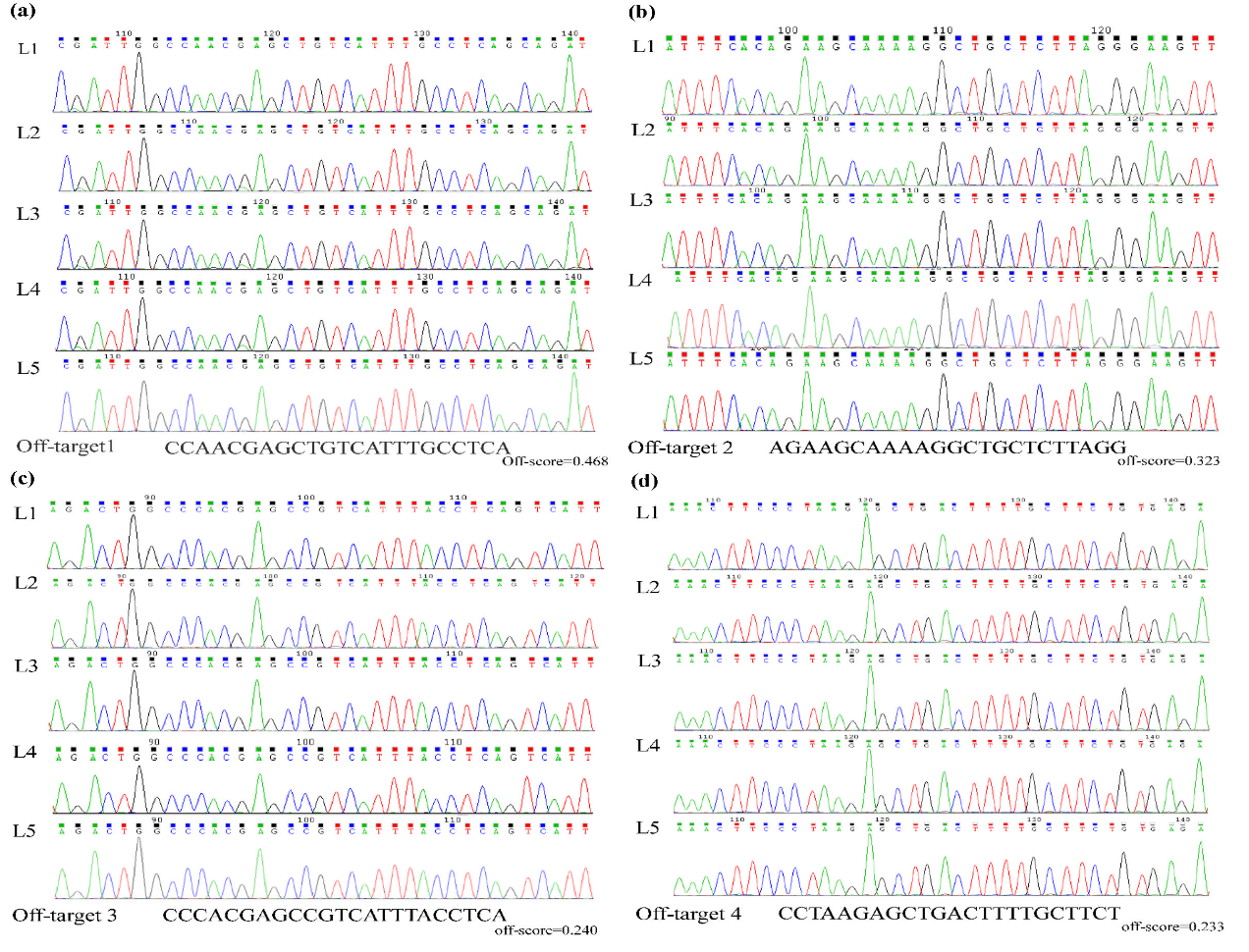
Figure S2. Off-target detection by sequencing in T1 plant

1. Analysis of potential off-target site 1 sequencing in five independent plants, no off-target events were found.
2. Analysis of potential off-target site 2 sequencing in five independent plants, no off-target events were found.
3. Analysis of potential off-target site 3 sequencing in five independent plants, no off-target events were found.
4. Analysis of potential off-target site 4 sequencing in five independent plants, no off-target events were found.

The sequencing results were analyzed by Chromas.

##
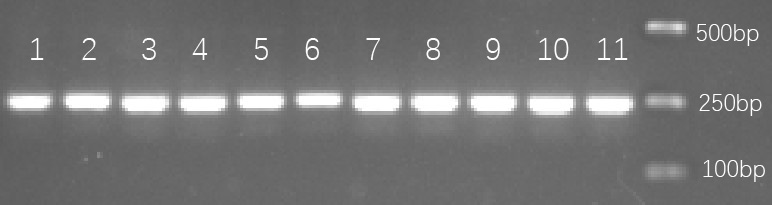
Figure S3. Off-target detection by T7EI assay in T4 plant

**Lane 1-10:** T7E1 digestion of the off-target 1 PCR fragments come from ten T4 plant. The off-sorce=0.468. No off-target events were found.

**Lane 11:** T7E1 digestion of the off-target 1 PCR fragments come from WT (negative control).

## Figure S4. Off-target detection by sequencing in T4 plant


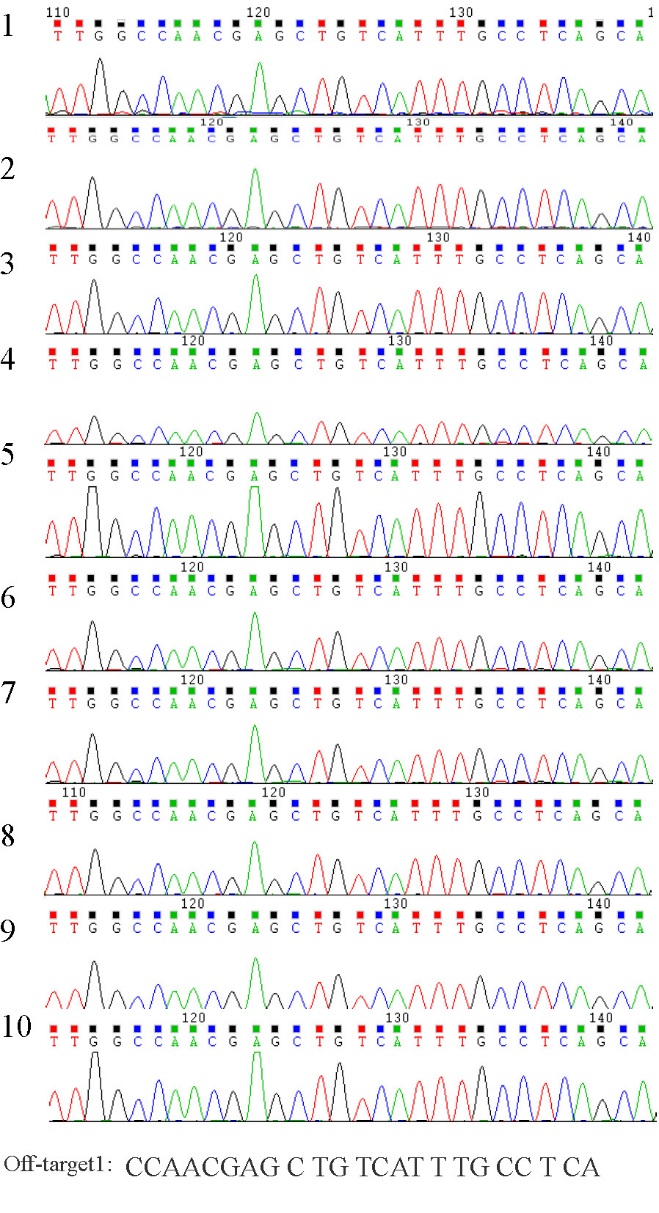


**Sequence 1-10:** Sequecing analysis of potential off-target site 1sequencing in ten independent T4 plants,The off-sorce=0.468.

No off-target events were found.

The sequencing results were analyzed by Chromas.

##
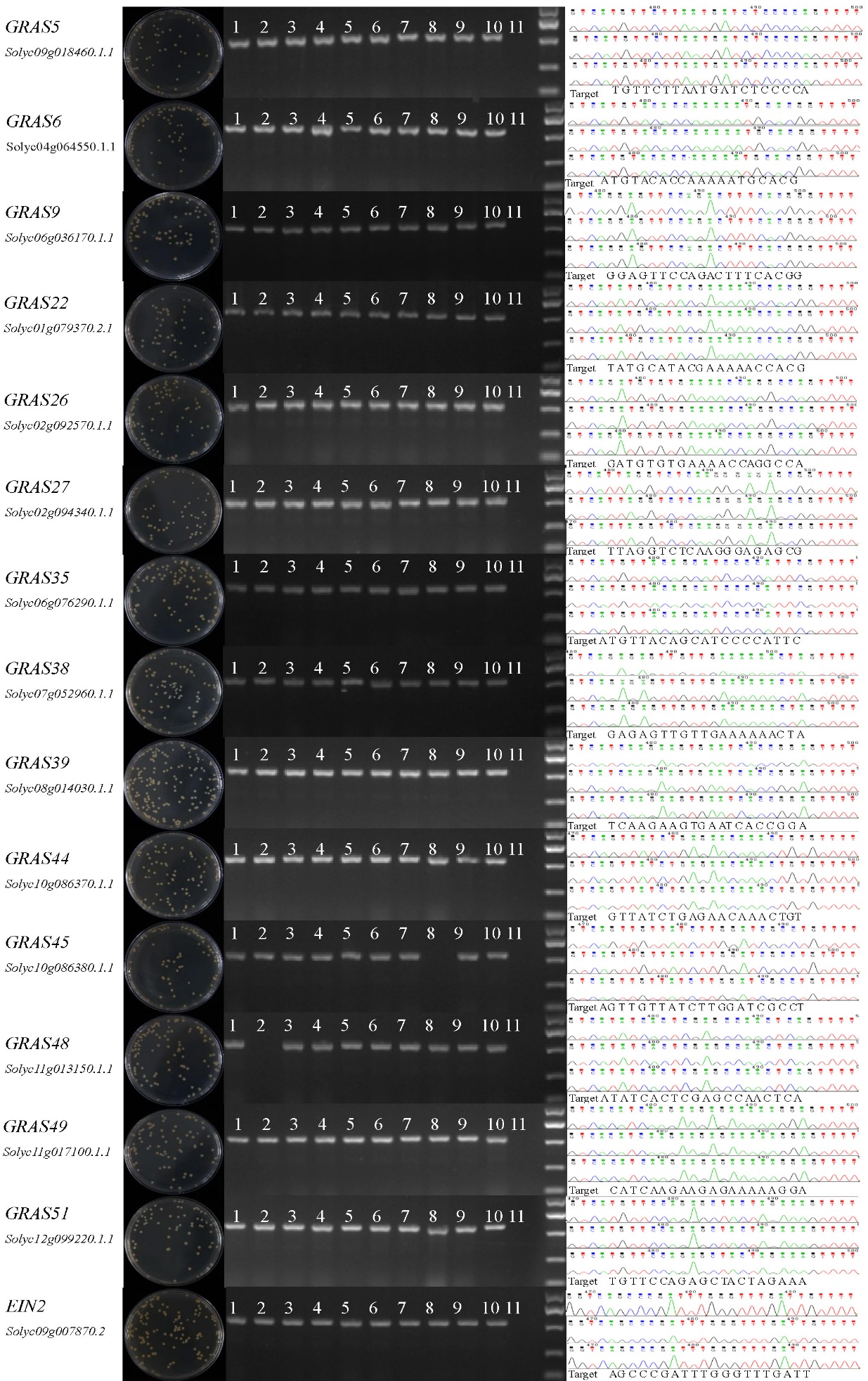
Figure S5. Transforming ligation reactions directly into *A. tumefaciens*

**Lane 1-10:** Positive colones were detected by colony PCR. **Lane 11:** negative control. Three positive clone sequencing result and the corresponding target sequence are located to the far right of the picture.

## Figure S6. Mutation efficiency analysis by direct sequencing of PCR products

Identification of mutation effectiveness by direct sequencing of PCR products containing targeted sites in tomato T0 plants. Black bars indicate PAM sequences. Red dot indicate the positions from where the mutations occurred.

*SlGRAS8*-target1

WT


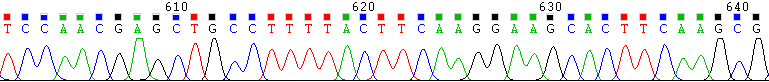


*SlGRAS8*-L2


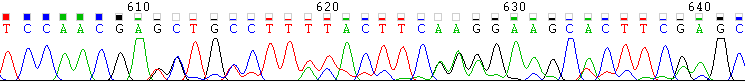


*SlGRAS8*-target2

WT


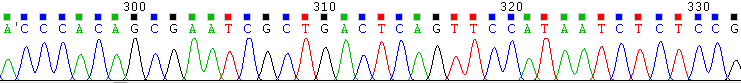

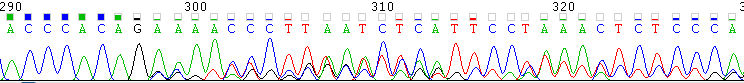

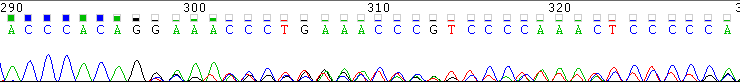


*SlGRAS8*-L1

*SlGRAS8*-L3

***SlGRAS8* mutant line**

**(a)**


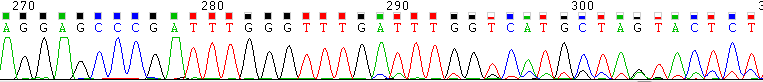

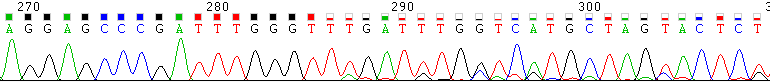

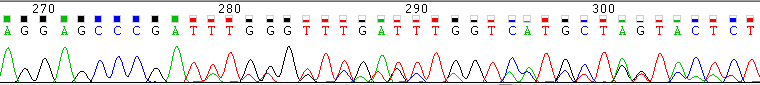

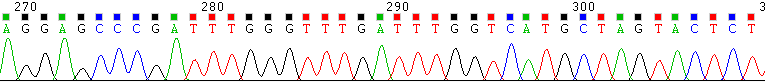


WT

*SlEIN2*-L3

*SlEIN2*-L5

*SlEIN2*-L8

***SlEIN2* mutant line**

**(b)**


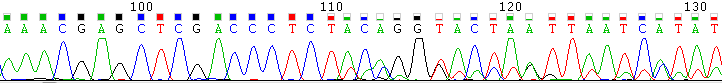

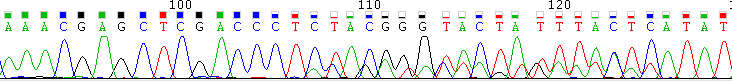

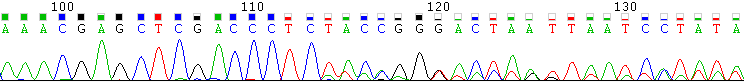

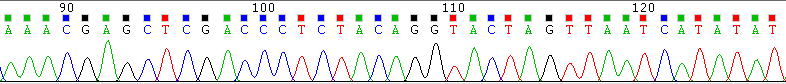


WT

*SlERFE1*-L1

*SlERFE1*-L4

*SlERFE1*-L7

***SlERFE1* mutant line**

**(c)**


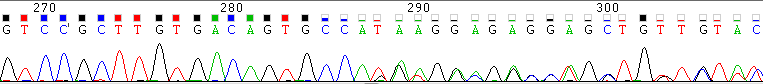

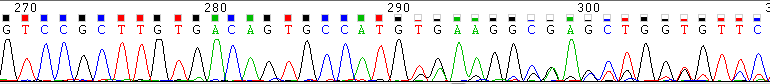

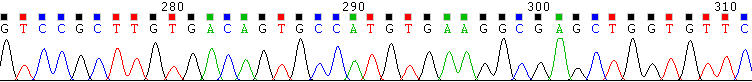


***SlARF2B* mutant line**

*SlARF2B*-L2

*SlARF2B*-L5

WT

**(d)**


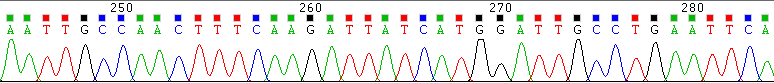

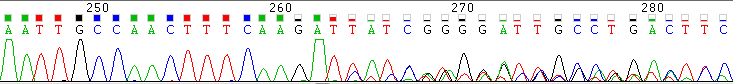

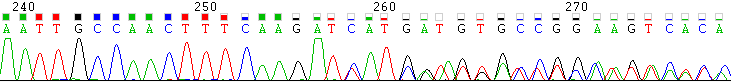

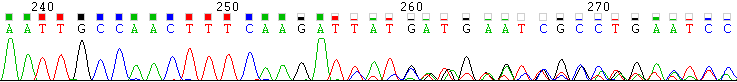

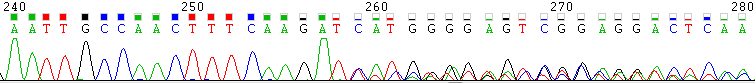


*SlACS2&4*-L2

*SlACS2&4*-L4

*SlACS2&4*-L6

*SlACS2&4*-L9

***SlACS4* mutant line**

*SlACS4*-WT

**(e)**


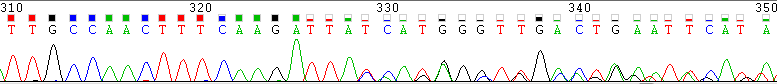

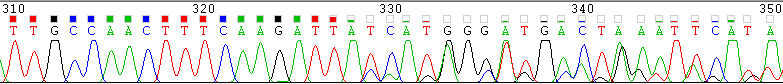

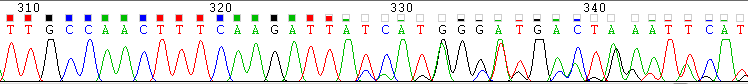

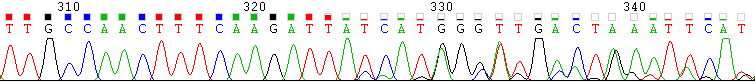

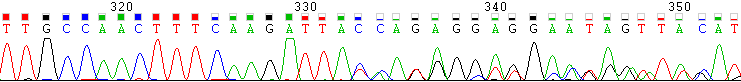

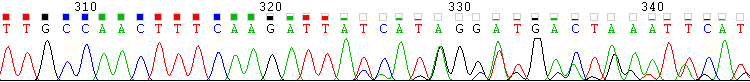


*SlACS2&4*-L1

*SlACS2&4*-L2

*SlACS2&4*-L4

*SlACS2&4*-L6

*SlACS2&4*-L7

*SlACS2&4*-L9

***SlACS2* mutant line**


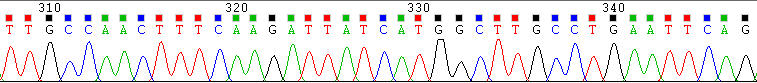


*SlACS2*-WT

**(f)**

## Figure S7. Assembly of Multiple sgRNA expression cassette

Ethidium bromide-stained gel with products obtained by restriction-ligation of the multiple sgRNA expression cassettes without acceptor vector (linearized pHNCas9S). M: Trans2Kplus DNA marker form Transgene of china. TheDNA bands from top to bottom are 5000bp, 3000bp, 2000bp, 1000bp, 750bp, 500bp, 250bp, 100bp. Red arrows indicate linear assembled DNA product. Restriction-ligation was performed at 22℃ for 0h, 1h, 3h, 6h, 9h, 12h, 24h.

(a) Assembly of two sgRNA expression cassettes, linear assembled product is about 900bp.

(b) Assembly of three sgRNA expression cassettes, linear assembled product is about 1150bp.

(c) Assembly of four sgRNA expression cassettes, linear assembled product is about 1600bp.

(d) Assembly of five sgRNA expression cassettes, linear assembled product is about 1850bp.

(e) Assembly of six sgRNA expression cassettes, linear assembled product is about 2100bp.

(f) Assembly of seven sgRNA expression cassettes, linear assembled product is about 2550bp.

(g) Assembly of eight sgRNA expression cassettes, linear assembled product is about 3000bp.


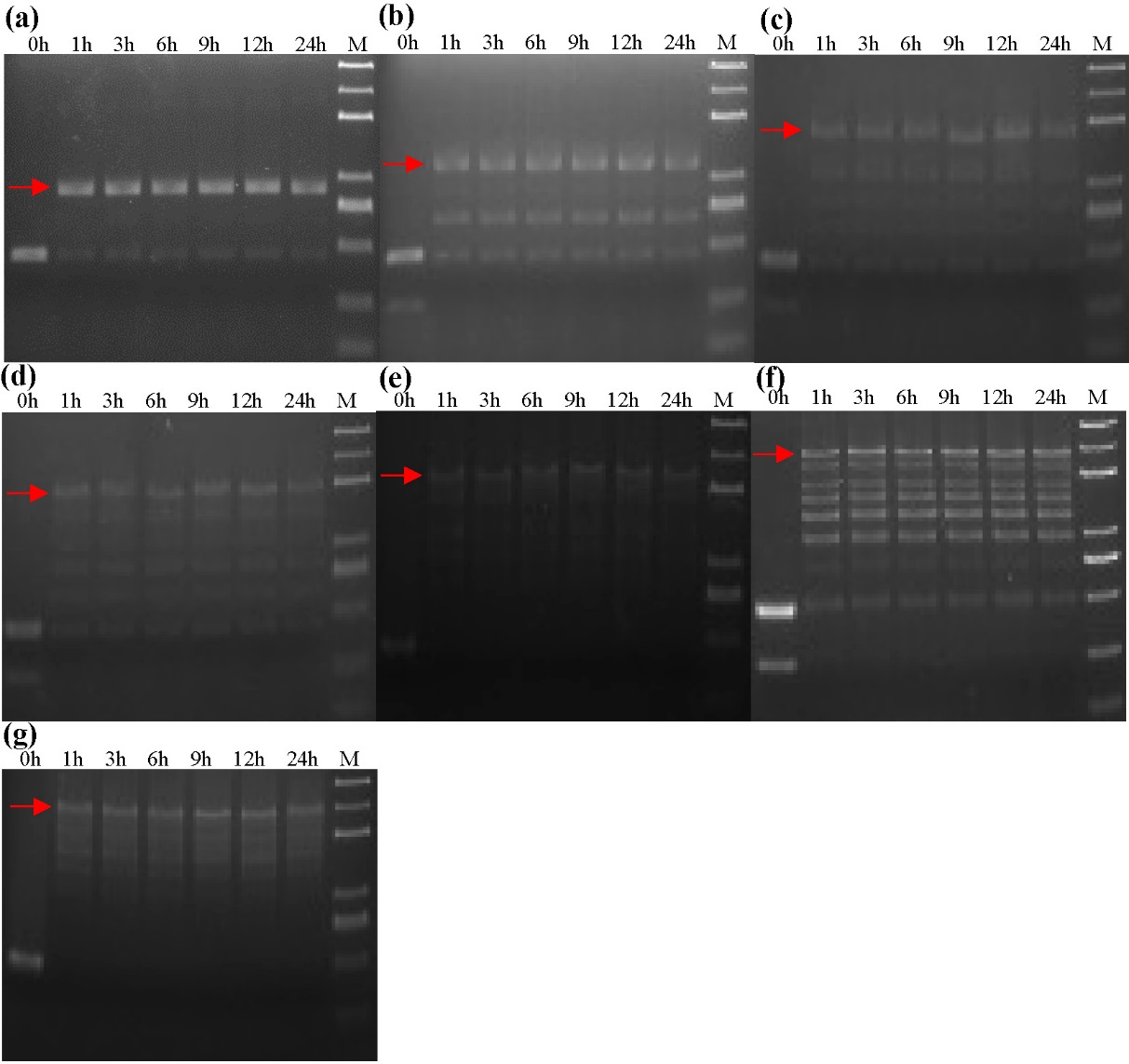


## Figure S8. Colony PCR for assembly of multiple sgRNA expression cassette with linearized pHNCas9S.


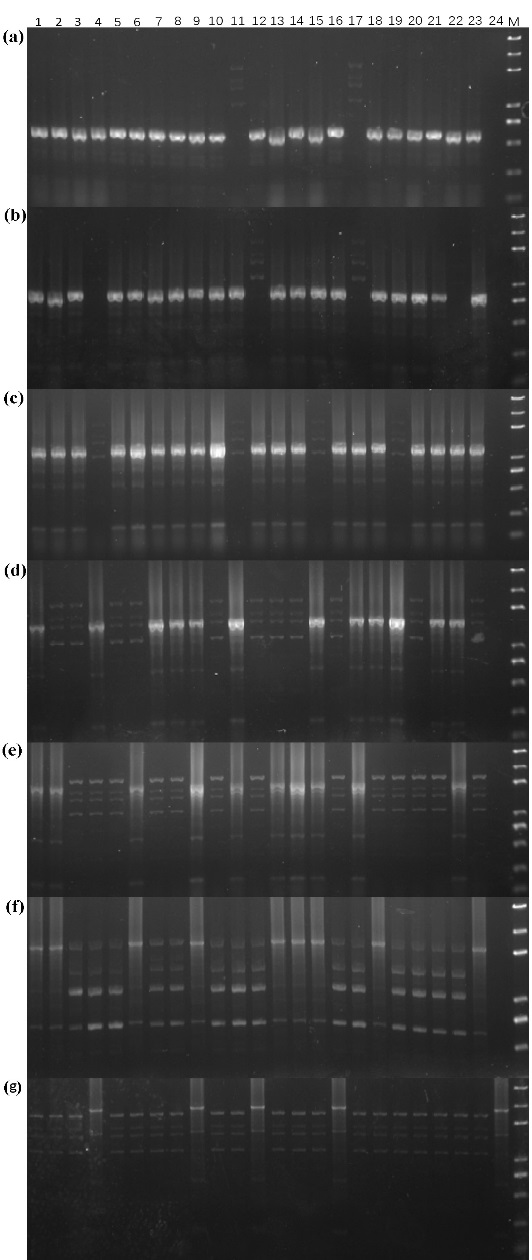
Assembly of multiple sgRNA expression cassettes into acceptor vector (linearized pHNCas9S) and transformation of TransT1 E. coli competent cell.

1. Assembly of two sgRNA expression cassettes,

target product is about 600bp.

The positive rate is 21/24.

1. Assembly of three sgRNA expression cassettes

target product is about 850bp.

The positive rate is 19/24.

1. Assembly of four sgRNA expression cassettes,

target product is about 1300bp.

The positive rate is 19/24.

1. Assembly of five sgRNA expression cassettes,

target product is about 1550bp.

The positive rate is 12/24.

1. Assembly of six sgRNA expression cassettes,

target product is about1900bp.

The positive rate is 10/24.

1. Assembly of seven sgRNA expression cassettes,

target product is about 2250bp.

The positive rate is 9/24.

1. Assembly of eight sgRNA expression cassettes,

target product is about 2700bp.

The positive rate is 5/24.

| **Assembly of multiple sgRNA expression cassettes in the Cas9VL** | | | | | | | |
| --- | --- | --- | --- | --- | --- | --- | --- |
| No.sgRNAs | Two | Three | Four | Five | Six | Seven | Eight |
| No. colonies | >4000 | >2000 | >1000 | >500 | ≈200 | ≈100 | ≈50 |
| Positive rate | 21/23 | 19/23 | 19/23 | 12/23 | 10/23 | 9/23 | 5/23 |
| Correct Sequencing | 3/3 | 3/3 | 3/3 | 3/3 | 3/3 | 3/3 | 3/3 |
| 5 U of T4 DNA ligase (Thermo, USA), 1 × T4 DNA ligase buffer, 50 ng of Cas9VL and 20 molar ratios of each digested sgRNA expression cassette were mixed in one-pot, total volume 10μl . Reactions were incubated at 22°C for 1 hour. | | | | | | | |

## Figure S9. Partial phenotypes and gene sequences of T4 generation *SlGRAS8* mutants

Target 1 of *SlGRAS8* was writen in red and **bold**. Target 2 of *SlGRAS8* was writen in blue and **bold**. Target 3 of *SlGRAS8* was writen in green and **bold**. Inverted sequence colored in purple.


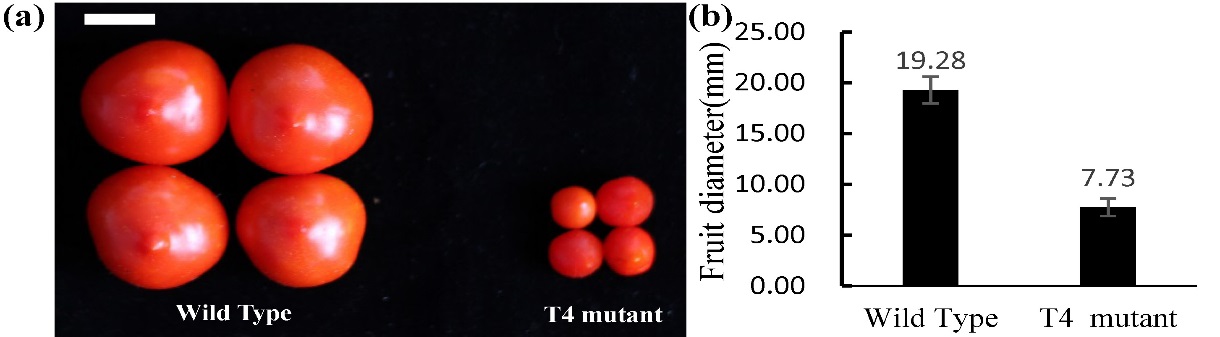


**Genotype of Wild Type:**

TTAACACCTCCAAGCTGACGTGGCTACTAGGGCCCTGTCATGCCAGCAAAGCAACATCTCTGCTTGTCTCTTTGCCACGTGGAATCCTCTTACCTGTACCCTACCCAATAAACAATCTGCTTGAAAATCGGCAAACTGGCTTAGTCCAACCGGCCGGAATCCGGCATCCACCATGGCATCCCTCCACGATCCTCCACCTCCACGCCGCCCTACTGCTCCGATCATATCAACTATCTTAGGAAACAGCACGAAATTCTCTATTTTTCTCATCCAATCCCCGCAATTTCCACCACCGATATTTGCTGCCTCCAGTGATTCCAGTAGTGTAGAGTAAAATTCCAGCCCGTCGATTACTGTCTGCCGGAAAGACATTGCACCGTAACCCATAAGTCCTTCGCTGTCCACGTGTACCACCACGTTAGGGGATATTCGACGGAGTTCATTTACAAACCCTGACCCGACCCGTCGGAAGATAGCGGGAGATAAAAGCACCGCTGTCTTCTCTCCCTCCATGAACTTTATCGCTTTGAAGGATAACAATTCGAATGTACGAATTAGGACAAAATCAATTTCGAAaCCGATAtTGAGTTCACGAGCAAATTGGGTTAAATTTtCTCTAATTaACCTTGATTCCACAGCGTACTCTTCAGGAACTAGAGCCGTAATTCTAAGGATCGGCGCGTTGGCTTTACGGCACTCGGCTTTGTCGGCTAACTCTTTCATGAAGGAAGCCCAGTGACCACCAAGTCCGATGTCGAAATCGATGACGTGGACTAGCATGGAGCCGTCAACTGCCTCAAGCACGGCCTGATTCGCCGTGAAGCTAGAGAACATAGGGATCGGAGAAATATTTGACAATATTTTATAAGATTTAATCGTTTGAATTACGTCGGAGGAACTTGAAGAACGAGTTTGCCGAGCTGATCCGGCGAGCTGCGCTTGAAGTGCTTCCTTGAAGTAAAAGGCAGCTCGTTGGAGAGGTTTTCCTGCTGCGGATCTGAGCCGTTGATTGAGGCGTGCCAGTATCACATGTGCGAGTTGGAAAGCGTTTGTTTCGAAACACTCAGCAAAACGAATGAGTTCATCCACATAATCAAATCCTACGCTCCAATTCTGGTGGATATCCCCGGAGAGATCAAGGGCGTTGATGAAACTTGCCTGATTCACCGTCGGAAACTGCTGAGGGAAATTAGTGTCTGAGAAAGAAAAATCAGGACTAGGAAACTGATTCGATTCAGCTGAAAACTCGGAGAGATTATGGA**ACTGAGTCAGCGATTCGCTGTGG**GTGAGAGGATTCGTTTTTGAAAGCGAAGCAGAATCGTCGTGCAAGCCAAGTTCTCTCATCAAAGAATCCCAATCTTCAAAGTTACTCAGAACATGATCTCCTAATTGAAGAGGATCTTCACCACCACAAAGAGCAGACAAATCAGGATTCGTTGTTATAATCTGCTTATCAACTATAGGACTTGGGCTACGACGGAGCTCGAGCACTGACTTAGGTTCGTAGCA**CAAATTAGAACCGTTGGTAGCGG**CTGGAAAAGTAAAGCTGTTGTTGCTGTTAACCAATGGCTTAGAACTTACGTTATCATTAGTGGAAAAGGGCACTTTCAT

**Genotype of L1-2 offspring (Data from T2 and T4 generations):**

TTAACACCTCCAAGCTGACGTGGCTACTAGGGCCCTGTCATGCCAGCAAAGCAACATCTCTGCTTGTCTCTTTGCCACGTGGAATCCTCTTACCTGTACCCTACCCAATAAACAATCTGCTTGAAAATCGGCAAACTGGCTTAGTCCAACCGGCCGGAATCCGGCATCCACCATGGCATCCCTCCACGATCCTCCACCTCCACGCCGCCCTACTGCTCCGATCATATCAACTATCTTAGGAAACAGCACGAAATTCTCTATTTTTCTCATCCAATCCCCGCAATTTCCACCACCGATATTTGCTGCCTCCAGTGATTCCAGTAGTGTAGAGTAAAATTCCAGCCCGTCGATTACTGTCTGCCGGAAAGACATTGCACCGTAACCCATAAGTCCTTCGCTGTCCACGTGTACCACCACGTTAGGGGATATTCGACGGAGTTCATTTACAAACCCTGACCCGACCCGTCGGAAGATAGCGGGAGATAAAAGCACCGCTGTCTTCTCTCCCTCCATGAACTTTATCGCTTTGAAGGATAACAATTCGAATGTACGAATTAGGACAAAATCAATTTCGAAaCCGATAtTGAGTTCACGAGCAAATTGGGTTAAATTTtCTCTAATTaACCTTGATTCCACAGCGTACTCTTCAGGAACTAGAGCCGTAATTCTAAGGATCGGCGCGTTGGCTTTACGGCACTCGGCTTTGTCGGCTAACTCTTTCATGAAGGAAGCCCAGTGACCACCAAGTCCGATGTCGAAATCGATGACGTGGACTAGCATGGAGCCGTCAACTGCCTCAAGCACGGCCTGATTCGCCGTGAAGCTAGAGAACATAGGGATCGGAGAAATATTTGACAATATTTTATAAGATTTAATCGTTTGAATTACGTCGGAGGAACTTGAAGAACGAGTTTGCCGAGCTGATCCGGCGAGCTGCGCTTGAAGTGCTTCCTTGAAGTAAAAGGCAGCT**TAGCGG**CTGGAAAAGTAAAGCTGTTGTTGCTGTTAACCAATGGCTTAGAACTTACGTTATCATTAGTGGAAAAGGGCACTTTCAT

**Genotype of L2-1 offspring (Data from T1 and T4 generations):**

TTAACACCTCCAAGCTGACGTGGCTACTAGGGCCCTGTCATGCCAGCAAAGCAACATCTCTGCTTGTCTCTTTGCCACGTGGAATCCTCTTACCTGTACCCTACCCAATAAACAATCTGCTTGAAAATCGGCAAACTGGCTTAGTCCAACCGGCCGGAATCCGGCATCCACCATGGCATCCCTCCACGATCCTCCACCTCCACGCCGCCCTACTGCTCCGATCATATCAACTATCTTAGGAAACAGCACGAAATTCTCTATTTTTCTCATCCAATCCCCGCAATTTCCACCACCGATATTTGCTGCCTCCAGTGATTCCAGTAGTGTAGAGTAAAATTCCAGCCCGTCGATTACTGTCTGCCGGAAAGACATTGCACCGTAACCCATAAGTCCTTCGCTGTCCACGTGTACCACCACGTTAGGGGATATTCGACGGAGTTCATTTACAAACCCTGACCCGACCCGTCGGAAGATAGCGGGAGATAAAAGCACCGCTGTCTTCTCTCCCTCCATGAACTTTATCGCTTTGAAGGATAACAATTCGAATGTACGAATTAGGACAAAATCAATTTCGAAaCCGATAtTGAGTTCACGAGCAAATTGGGTTAAATTTtCTCTAATTaACCTTGATTCCACAGCGTACTCTTCAGGAACTAGAGCCGTAATTCTAAGGATCGGCGCGTTGGCTTTACGGCACTCGGCTTTGTCGGCTAACTCTTTCATGAAGGAAGCCCAGTGACCACCAAGTCCGATGTCGAAATCGATGACGTGGACTAGCATGGAGCCGTCAACTGCCTCAAGCACGGCCTGATTCGCCGTGAAGCTAGAGAACATAGGGATCGGAGAAATATTTGACAATATTTTATAAGATTTAATCGTTTGAATTACGTCGGAGGAACTTGAAGAACGAGTTTGCCGAGCTGATCCGGCGAGCTGCGCTTGAAGTGCTT**TAGCGG**CTGGAAAAGTAAAGCTGTTGTTGCTGTTAACCAATGGCTTAGAACTTACGTTATCATTAGTGGAAAAGGGCACTTTCAT

**Genotype of L3-3 offspring ((Data from T1 and T4 generations):**

TTAACACCTCCAAGCTGACGTGGCTACTAGGGCCCTGTCATGCCAGCAAAGCAACATCTCTGCTTGTCTCTTTGCCACGTGGAATCCTCTTACCTGTACCCTACCCAATAAACAATCTGCTTGAAAATCGGCAAACTGGCTTAGTCCAACCGGCCGGAATCCGGCATCCACCATGGCATCCCTCCACGATCCTCCACCTCCACGCCGCCCTACTGCTCCGATCATATCAACTATCTTAGGAAACAGCACGAAATTCTCTATTTTTCTCATCCAATCCCCGCAATTTCCACCACCGATATTTGCTGCCTCCAGTGATTCCAGTAGTGTAGAGTAAAATTCCAGCCCGTCGATTACTGTCTGCCGGAAAGACATTGCACCGTAACCCATAAGTCCTTCGCTGTCCACGTGTACCACCACGTTAGGGGATATTCGACGGAGTTCATTTACAAACCCTGACCCGACCCGTCGGAAGATAGCGGGAGATAAAAGCACCGCTGTCTTCTCTCCCTCCATGAACTTTATCGCTTTGAAGGATAACAATTCGAATGTACGAATTAGGACAAAATCAATTTCGAAaCCGATAtTGAGTTCACGAGCAAATTGGGTTAAATTTtCTCTAATTaACCTTGATTCCACAGCGTACTCTTCAGGAACTAGAGCCGTAATTCTAAGGATCGGCGCGTTGGCTTTACGGCACTCGGCTTTGTCGGCTAACTCTTTCATGAAGGAAGCCCAGTGACCACCAAGTCCGATGTCGAAATCGATGACGTGGACTAGCATGGAGCCGTCAACTGCCTCAAGCACGGCCTGATTCGCCGTGAAGCTAGAGAACATAGGGATCGGAGAAATATTTGACAATATTTTATAAGATTTAATCGTTTGAATTACGTCGGAGGAACTTGAAGAACGAGTTTGCCGAGCTGATCCGGCGAGCTGCGCTTGAAGTGCTTCCTTGAAGTAAAAGGCAGCTCCAACGGTTCTAATTTGTGCTACGAACCTAAGTCAGTGCTCGAGCTCCGTCGTAGCCCAAGTCCTATAGTTGATAAGCAGATTATAACAACGAATCCTGATTTGTCTGCTCTTTGTGGTGGTGAAGATCCTCTTCAATTAGGAGATCATGTTCTGAGTAACTTTGAAGATTGGGATTCTTTGATGAGAGAACTTGGCTTGCACGACGATTCTGCTTCGCTTTCAAAAACGAATCCTCTCACCCACAGGAATCGCTGACTCAGTTCCATAATCTCTCCGAGTTTTCAGCTGAATCGAATCAGTTTCCTAGTCCTGATTTTTCTTTCTCAGACACTAATTTCCCTCAGCAGTTTCCGACGGTGAATCAGGCAAGTTTCATCAACGCCCTTGATCTCTCCGGGGATATCCACCAGAATTGGAGCGTAGGATTTGATTATGTGGATGAACTCATTCGTTTTGCTGAGTGTTTCGAAACAAACGCTTTCCAACTCGCACATGTGATACTGGCACGCCTCAATCAACGGCTCAGATCCGCAGCAGGAAAACCTCTCCAACGA**TAGCGG**CTGGAAAAGTAAAGCTGTTGTTGCTGTTAACCAATGGCTTAGAACTTACGTTATCATTAGTGGAAAAGGGCACTTTCAT

## Table S1. Primers used for PCR of AtU3s/AtU6s and sgRNA

| Primer name | Primer sequences (5’-3’) | Purpose |
| --- | --- | --- |
| AtU-F | CCTTTGGAATCGGCAGCAAAGG | PCR of AtU3s/AtU6s promoter |
| AtU3b-R | TGACCAATGTTGCTCCCTC | PCR of AtU3b promoter |
| AtU3d-R | TGACCAATGGTGCTTTGTAG | PCR of AtU3d promoter |
| AtU6-1-R | CAATCACTACTTCGTCTCT | PCR of AtU6-1 promoter |
| AtU6-26-R | CAATCACTACTTCGACTCT | PCR of AtU6-26 promoter |
| AtU6-29-R | CAATCTCTTAGTCGACTCTAC | PCR of AtU6-29 promoter |
| sg-F | GTTTTAGAGCTAGAAATAGC | PCR of sgRNA |
| sg-R | CTTCCATCCACTCCAAGCTC | PCR of sgRNA |
| pHN-JC-F | GAACCCTGTGGTTGGCATGC | PCR detection for pHNCas9SHT |
| pHN-JC-R | CGTATATACCGGCATGTGG | For sequening of multisite cloning |
| ACS2-R | TTGGGGTTTTTTAGAGGGTG | For sequening of multisite cloning |
| ACS3-R | CCCCAGTTCTCCATTTGAGG | For sequening of multisite cloning |
| ACS4-R | CGAGACCCATCTGAATAAC | For sequening of multisite cloning |
| ACS6-R | GTTGTTTGTGAGAGTTCTAA | For sequening of multisite cloning |
| ACS7-R | CGCTGATCCTGGCCATGCT | For sequening of multisite cloning |
| ACS1-F | GGTCTTGCTGAAAATCAGGT | Colony PCR for multiple sgRNA expression cassettes assembly |
| ACS2-F | CACCCTCTAAAAAACCCCAA | Colony PCR for two sgRNA expression cassettes assembly |

## Table S2. *Bsa* I-site Primers used for one-step PCR of sgRNA expression cassettes

*bsaI* I site colored in red, *BsaI* restriction overhangs colored in blue. **GGTACC**, **TCTAGA**, and **CTCGAG** in the Ts’ are *Kpn* I, *Xba* I, *Xho* I, respectively. **GTCGAC** and **ACTAGT** in the TE are *Sal* I and *Spe* I, respectively.

| Position | Primer | Sequence (5’--3’) |
| --- | --- | --- |
| Site1(S’1) | TS’ | CGTGGGTCTCTCTCG**GGTACC**CCG**TCTAGA**GG**CTCG****AG**CCTTTGGAATCGGCAGCAAAG |
|  | T1 | CGTGGGTCTCTCATTTTCCATCCACTCCAAGCTCTTG |
| Site2(1’2) | T1’ | CGTGGGTCTCTAATGCCTTTGGAATCGGCAGCAAAGG |
|  | T2 | CGTGGGTCTCTCTATTTCCATCCACTCCAAGCTCTTG |
| Site3(2’3) | T2’ | CGTGGGTCTCTATAGCCTTTGGAATCGGCAGCAAAGG |
|  | T3 | CGTGGGTCTCTCCCTTTCCATCCACTCCAAGCTCTTG |
| Site4(3’4) | T3’ | CGTGGGTCTCTAGGGCCTTTGGAATCGGCAGCAAAGG |
|  | T4 | CGTGGGTCTCTGATATTCCATCCACTCCAAGCTCTTG |
| Site5(4’5) | T4’ | CGTGGGTCTCTTATCCCTTTGGAATCGGCAGCAAAGG |
|  | T5 | CGTGGGTCTCTGTAATTCCATCCACTCCAAGCTCTTG |
| Site6(5’6) | T5’ | CGTGGGTCTCTTTACCCTTTGGAATCGGCAGCAAAGG |
|  | T6 | CGTGGGTCTCTGCGATTCCATCCACTCCAAGCTCTTG |
| Site7(6’7) | T6’ | CGTGGGTCTCTTCGCCCTTTGGAATCGGCAGCAAAGG |
|  | T7 | CGTGGGTCTCTGGCATTCCATCCACTCCAAGCTCTTG |
| Site8(7’E) | T7’ | CGTGGGTCTCTTGCCCCTTTGGAATCGGCAGCAAAGG |
|  | TE | CGTGGGTCTCTACCG**GTCGAC**GACCGAACAAGC**ACTAGT**TTCCATCCACTCCAAGCTCT |
|  | **Bb-F** | CTCG**GGTACC**CCG**TCTAGA**GG**CTCG** |
|  | **Bb-R** | ACCG**GTCGAC**GACCGAACAAGC**ACT** |

**Note.**

**(1)** **The *Bsa* I-cutting non-palindromic ends with the X and X’ are compatible for ligation.**

(2) The first sgRNA cassette must start with **TS’** and the last sgRNA cassette must end with **TE’**.

(3) *Kpn* I, *Xba* I, *Xho* I in the TS and *Sal* I, *Spe* I in the TE are used for Biobricks Assembly. Using this Isocaudarner-dependent assembly strategy, multiple sgRNA expression cassettes groups can be assembled into the same binary vector.

(4) Bb-F and Bb-R were used to amplify sgRNA expression cassettes groups for Standard Assembly of Biobricks.

## ****Table S3. Rules of using *Bsa* I site primer****

1. **Multiple sgRNA expression cassettes are amplified with the primer pairs in this way:**

|  | **Target1** | **Target2** | **Target3** | **Target4** | **Target5** | **Target6** | **Target7** | **Target8** |
| --- | --- | --- | --- | --- | --- | --- | --- | --- |
| **Two sgRNA** | **Ts’&T1** | **T1’&TE** |  |  |  |  |  |  |
| **Three sgRNA** | **Ts’&T1** | **T1’&T2** | **T2’&TE** |  |  |  |  |  |
| **Four sgRNA** | **Ts’&T1** | **T1’&T2** | **T2’&T3** | **T3’&TE** |  |  |  |  |
| **Five sgRNA** | **Ts’&T1** | **T1’&T2** | **T2’&T3** | **T3’&T4** | **T4’&TE** |  |  |  |
| **Six sgRNA** | **Ts’&T1** | **T1’&T2** | **T2’&T3** | **T3’&T4** | **T4’&T5** | **T5’&TE** |  |  |
| **Seven sgRNA** | **Ts’&T1** | **T1’&T2** | **T2’&T3** | **T3’&T4** | **T4’&T5** | **T5’&T6** | **T6’&TE** |  |
| **Eight sgRNA** | **Ts’&T1** | **T1’&T2** | **T2’&T3** | **T3’&T4** | **T4’&T5** | **T5’&T6** | **T6’&T7** | **T7’&TE** |

## ****Table S4.**** ****Components of CRISPR kit****

| Name | Concentration | Purpose |
| --- | --- | --- |
| AtU3b-sgRNA | 1ng/µl-1ng/µl | Mix templates for amplifing sgRNA sexpression cassette by PCR |
| AtU3d-sgRNA | 1ng/µl-1ng/µl | Mix templates for amplifing sgRNA sexpression cassette by PCR |
| AtU6-1-sgRNA | 1ng/µl-1ng/µl | Mix templates for amplifing sgRNA sexpression cassette by PCR |
| AtU6-26-sgRNA | 1ng/µl-1ng/µl | Mix templates for amplifing sgRNA sexpression cassette by PCR |
| AtU6-29-sgRNA | 1ng/µl-1ng/µl | Mix templates for amplifing sgRNA sexpression cassette by PCR |
| Cas9VL | 25ng/µl | Linearized pHNCas9 for assembling multiple sgRNA expression cassettes |
| Cas9VLHT | 25ng/µl | Linearized pHNCas9HT for assembling single sgRNA target site |
| *Bsa* I-site Primers | 10µM | Primer for amplifing sgRNA sexpression cassette by PCR |
| Excel S1 |  | For designing primers and Generation of sgRNA Sequences for Secondary Structure Prediction |

## ****Table S5 Primers used for introduction of target sequences into sgRNA expression cassettes by one-step PCR****

Target sequence written in underlined and **bold.**

| Primer | Sequence(5’--3’) | Promoter |
| --- | --- | --- |
| *SlGRAS8*-AtU3b-F | GAGGGAGCAACATTGGTC**ATGAAGTAAAAGGCAGCTCGT**GTTTTAGAGCTAGAAATAG | AtU3b |
| *SlGRAS8*-AtU3d-F | CTACAAAGCACCATTGGTC**ACTGAGTCAGCGATTCGCTG**GTTTTAGAGCTAGAAATAG | AtU3d |
| *SlGRAS8*-AtU6-29-F | TAGAGTCGACTAAGAGATT**GCAAATTAGAACCGTTGGTAG**GTTTTAGAGCTAGAAATAG | AtU6-29 |
| *SlACS2*&4-AtU3b-F | GAGGGAGCAACATTGGTCA**GCTTTCCACCCATCAAAATA**GTTTTAGAGCTAGAAATAG | AtU3b |
| *SlACS2*&4-AtU3d-F | CTACAAAGCACCATTGGTCA**GCCAACTTTCAAGATTATCA**GTTTTAGAGCTAGAAATAG | AtU3d |

## ****Table S6**** ****Primer for amplifing and sequencing**** of genomic fragment that contain editing sites

| Primer name | Primer sequences (5’-3’) | Purpose |
| --- | --- | --- |
| SlGRAS8-1F | ATGAAAGTGCCCTTTTCC | PCR and sequence for target genomic fragment of *SlGRAS8* CRISPR lilne |
| SlGRAS8-3R | AGGAACTAGAGCCGTAATTC | PCR for target genomic fragment of *SlGRAS8* CRISPR lilne |
| EIN2-F | CTACAGTCTACAGTTGGAGC | PCR and sequence for target genomic fragment of *SlEIN2* CRISPR lilne |
| EIN2-R | AAGAGCAGGAGAATCAACTT | PCR for target genomic fragment of *SlEIN2* CRISPR lilne |
| ERFE1-F | GTGTGGTGGTGCAATTCTTGC | PCR and sequence for target genomic fragment of *SlERF.E1* CRISPR lilne |
| ERFE1-R | ATTGGGGAAATTAACTTTAGC | PCR for target genomic fragment of *SlERF.E1* CRISPR lilne |
| ARF2B-F | TGTACTGGAAGTGTTTGGAG | PCR and sequence for target genomic fragment of *SlARF2B* CRISPR lilne |
| ARF2B-R | TAGGCATGACTGATGAATGG | PCR for target genomic fragment of *SlARF2B* CRISPR lilne |
| ACS2-F | GATTGCAAAGACCAACTCAATC | PCR and sequence for *SlACS2* target genomic fragment of *SlACS2&4* CRISPR lilne |
| ACS2-R | AGCCATAACAACTCTTTCTGG | PCR for *SlACS2* target genomic fragment of *SlACS2&4* CRISPR lilne |
| ACS4-F | ATGGATTTGGAGACGAGTGAG | PCR and sequence for *SlACS4* target genomic fragment of *SlACS2&4* CRISPR lilne |
| ACS4-R | CAGCCATTACTACACGTTTAGC | PCR for *SlACS2* target genomic fragment of *SlACS2&4* CRISPR lilne |

## ****Table S7. Primers for**** ****amplification of potential off target sites****

| Primer | Sequences (5’-3’) | Purpose |
| --- | --- | --- |
| Off1-F | CAACGACTTGGCCACACACG | PCR of off-target 1 |
| Off1-R | CAAGGACAAGCCAAGATTCC | PCR of off-target 1 |
| Off2-F | GGCTATTATCAATTTGAGTG | PCR of off-target 2 |
| Off2-R | GGTGCTTGGTCAAACGCTTG | PCR of off-target 2 |
| Off3-F | CTGTTTGTTCATAATTGGAC | PCR of off-target 3 |
| Off3-R | AAACGTCCAGGCAAACATCC | PCR of off-target 3 |
| Off4-F | AGCATGTAAGGTAGCTAGTC | PCR of off-target 4 |
| Off4-R | GTTACTGAATTTTGTGGATG | PCR of off-target 4 |

## ****Table S8. Primers used for expression analysis****

| Primer | Sequences (5’-3’) | Purpose |
| --- | --- | --- |
| *SlActin*-RT-R | TCTCGGTGAGGATCTTCATC | RT of *SlActin* |
| *SlActin*-qPCR-F | GTATGCTAGTGGTCGTACAAC | RT-qPCR of *SlActin*(Control) |
| *SlActin*-qPCR-R | AGTTAAATCACGACCAGCAAG | RT-qPCR of *SlActin*(Control) |
| *Cas9S*-RT | ACTTTATAGTCACCGTACAC | RT of *Cas9S* |
| *SlGRAS8*-U3b-qPCR-F | GTCATGAAGTAAAAGGCAGCTCGT | RT-qPCR of *SlGRAS8*-sgRNA1 |
| *SlGRAS8*-U3d-qPCR-F | GTCACTGAGTCAGCGATTCGCTG | RT-qPCR of *SlGRAS8*-sgRNA2 |
| *SlGRAS8*-U629-qPCR-F | ATTGCAAATTAGAACCGTTGGTAG | RT-qPCR of *SlGRAS8*-sgRNA3 |
| gRNA-RT&qPCR-R | CGACTCGGTGCCACTTTTTCAAGTTG | RT of sgRNA & qPCR of sgRNAs together with target-specific primers |
| ARF2B-U3b | GAGAATTGCCGGAACGCTGC | RT-qPCR of *SlARF2B*-sgRNA |
| EIN2-U3b | AGCCCGATTTGGGTTTGATTG | RT-qPCR of *SlEIN2*-sgRNA |
| ERFE1-U3b | AAACGAGCTCGACCCTCTAC | RT-qPCR of *SlERFE1*-sgRNA |
| ACS24-U3b | GctttccacccatcaaaataG | RT-qPCR of *SlACS2&4*-sgRNA1 |
| ACS24-U3d | aaatttatggagaaaacaagG | RT-qPCR of *SlACS2&4*-sgRNA2 |

## ****Table S9. Primers for transforming ligation reactions product directly into *A. tumefaciens*****

| Primer | Sequences (5’-3’) |
| --- | --- |
| GRAS5-Cas9-F | GTCATGTTCTTAATGATCTCCCCA |
| GRAS5-Cas9-R | AAACTGGGGAGATCATTAAGAACA |
| GRAS6-Cas9-F | GTCATGTACACCAAAAATGCACG |
| GRAS6-Cas9-R | AAACCGTGCATTTTTGGTGTACA |
| GRAS9-Cas9-F | GTCAGGAGTTCCAGACTTTCACGG |
| GRAS9-Cas9-R | AAACCCGTGAAAGTCTGGAACTCC |
| GRAS22-Cas9-F | GTCATATGCATACGAAAAACCACG |
| GRAS22-Cas9-R | AAACCGTGGTTTTTCGTATGCATA |
| GRAS26-Cas9-F | GTCAGATGTGTGAAAACCAGGCCA |
| GRAS26-Cas9-R | AAACTGGCCTGGTTTTCACACATC |
| GRAS27-Cas9-F | GTCATTAGGTCTCAAGGGAGAGCG |
| GRAS27-Cas9-R | AAACCGCTCTCCCTTGAGACCTAA |
| GRAS35-Cas9-F | GTCATGTTACAGCATCCCCATTC |
| GRAS35-Cas9-R | AAACGAATGGGGATGCTGTAACA |
| GRAS38-Cas9-F | GTCAGAGAGTTGTTGAAAAAACTA |
| GRAS38-Cas9-R | AAACTAGTTTTTTCAACAACTCTC |
| GRAS39-Cas9-F | GTCATCAAGAAGTGAATCACCGGA |
| GRAS39-Cas9-R | AAACTCCGGTGATTCACTTCTTGA |
| GRAS44-Cas9-F | GTCAGTTATCTGAGAACAAACTGT |
| GRAS44-Cas9-R | AAACACAGTTTGTTCTCAGATAAC |
| GRAS45-Cas9-F | GTCAGTTGTTATCTTGGATCGCCT |
| GRAS45-Cas9-R | AAACAGGCGATCCAAGATAACAAC |
| GRAS48-Cas9-F | GTCATATCACTCGAGCCAACTCA |
| GRAS48-Cas9-R | AAACTGAGTTGGCTCGAGTGATA |
| GRAS49-Cas9-F | GTCACATCAAGAAGAGAAAAAGGA |
| GRAS49-Cas9-R | AAACTCCTTTTTCTCTTCTTGATG |
| GRAS51-Cas9-F | GTCATGTTCCAGAGCTACTAGAAA |
| GRAS51-Cas9-R | AAACTTTCTAGTAGCTCTGGAACA |
| EIN2-Cas9-F | GTCAGCCCGATTTGGGTTTGATT |
| EIN2-Cas9-R | AAACAATCAAACCCAAATCGGGC |
| ERFE1-Cas9-F | GTCAAACGAGCTCGACCCTCTAC |
| ERFE1-Cas9-R | AAACGTAGAGGGTCGAGCTCGTT |
| ARF2B-Cas9-F | GTCACTTGTGACAGTGCCATGTGA |
| ARF2B-Cas9-R | AAACTCACATGGCACTGTCACAAG |

## ****Table S10. Primers for multiple sgRNA expression cassette assembly****

The following primers were used to generate the expression cassette sgRNA by one-step PCR. The target site of the gene involved in the primer name is written in red.

| Gene name | Gene No. | Primer name | Sequences (5’-3’) |
| --- | --- | --- | --- |
| *SlACS1* | Solyc08g081540.2 | SlACS1-AtU3b-F | GAGGGAGCAACATTGGTCAGGTCTTGCTGAAAATCAGGTGTTTTAGAGCTAGAAATAG |
| *SlACS2* | Solyc01g095080.2 | SlACS2-AtU3b-F | GAGGGAGCAACATTGGTCACACCCTCTAAAAAACCCCAAGTTTTAGAGCTAGAAATAG |
| *SlACS3* | Solyc02g091990.2 | SlACS3-AtU3d-F | CTACAAAGCACCATTGGTCACCTCAAATGGAGAACTGGGGGTTTTAGAGCTAGAAATAG |
| *SlACS4* | Solyc05g050010.2 | SlACS4-AtU6-26-F | TAGAGTCGAAGTAGTGATTGCGATAACGACCCTTTCCACTGTTTTAGAGCTAGAAATAG |
| *SlACS5* | Solyc04g077410.2 | SlACS5-AtU3d-F | CTACAAAGCACCATTGGTCAATAAGAAGAATCTTGCCCATGTTTTAGAGCTAGAAATAG |
| *SlACS6* | Solyc08g008100.2 | SlACS6-AtU6-26-F | TAGAGTCGAAGTAGTGATTGTTAGAACTCTCACAAACAACGTTTTAGAGCTAGAAATAG |
| *SlACS7* | Solyc02g063540.1 | SlACS7-AtU3d-F | CTACAAAGCACCATTGGTCAAGCATGGCCAGGATCAGCGGTTTTAGAGCTAGAAATAG |
| *SlACS8* | Solyc03g043890.2 | SlACS8-AtU6-26-F | TAGAGTCGAAGTAGTGATTGTTCCTACCCCTTACTACCCGGTTTTAGAGCTAGAAATAG |

## ****Table S11. The sgRNA expression cassettes for multiple assembly in one-pot****

1. **Multiple sgRNA expression cassettes are amplified with the primer pairs in this way:**

|  | **Target1** | **Target2** | **Target3** | **Target4** | **Target5** | **Target6** | **Target7** | **Target8** |
| --- | --- | --- | --- | --- | --- | --- | --- | --- |
| **Two sgRNA** | *SlACS2*  **Ts’&T1** | *SlACS4*  **T1’&TE** |  |  |  |  |  |  |
| **Three sgRNA** | *SlACS1*  **Ts’&T1** | *SlACS3*  **T1’&T2** | *SlACS4*  **T2’&TE** |  |  |  |  |  |
| **Four sgRNA** | *SlACS1*  **Ts’&T1** | *SlACS2*  **T1’&T2** | *SlACS3*  **T2’&T3** | *SlACS4*  **T3’&TE** |  |  |  |  |
| **Five sgRNA** | *SlACS1*  **Ts’&T1** | *SlACS2*  **T1’&T2** | *SlACS3*  **T2’&T3** | *SlACS5*  **T3’&T4** | *SlACS4*  **T4’&TE** |  |  |  |
| **Six sgRNA** | *SlACS1*  **Ts’&T1** | *SlACS2*  **T1’&T2** | *SlACS3*  **T2’&T3** | *SlACS5*  **T3’&T4** | *SlACS4*  **T4’&T5** | *SlACS6*  **T5’&TE** |  |  |
| **Seven sgRNA** | *SlACS1*  **Ts’&T1** | *SlACS2*  **T1’&T2** | *SlACS3*  **T2’&T3** | *SlACS5*  **T3’&T4** | *SlACS7*  **T4’&T5** | *SlACS4*  **T5’&T6** | *SlACS6*  **T6’&TE** |  |
| **Eight sgRNA** | *SlACS1*  **Ts’&T1** | *SlACS2*  **T1’&T2** | *SlACS3*  **T2’&T3** | *SlACS5*  **T3’&T4** | *SlACS7*  **T4’&T5** | *SlACS4*  **T5’&T6** | *SlACS6*  **T6’&T7** | *SlACS8*  **T7’&TE** |
